# Supplementary material for: The ZmWAKL–ZmWIK–ZmBLK1–ZmRBOH4 module provides quantitative resistance to gray leaf spot in maize
Source: Nat Genet. 2024 Jan 18;56(2):315–26. doi: 10.1038/s41588-023-01644-z (PMC10864183; doi:10.1038/s41588-023-01644-z)

nature genetics

Article

https://doi.org/10.1038/s41588-023-01644-z

The ZmWAKL–ZmWIK–ZmBLK1–ZmRBOH4 module provides quantitative resistance to gray leaf spot in maize

In the format provided by the authors and unedited

Supplementary Figures 1-8

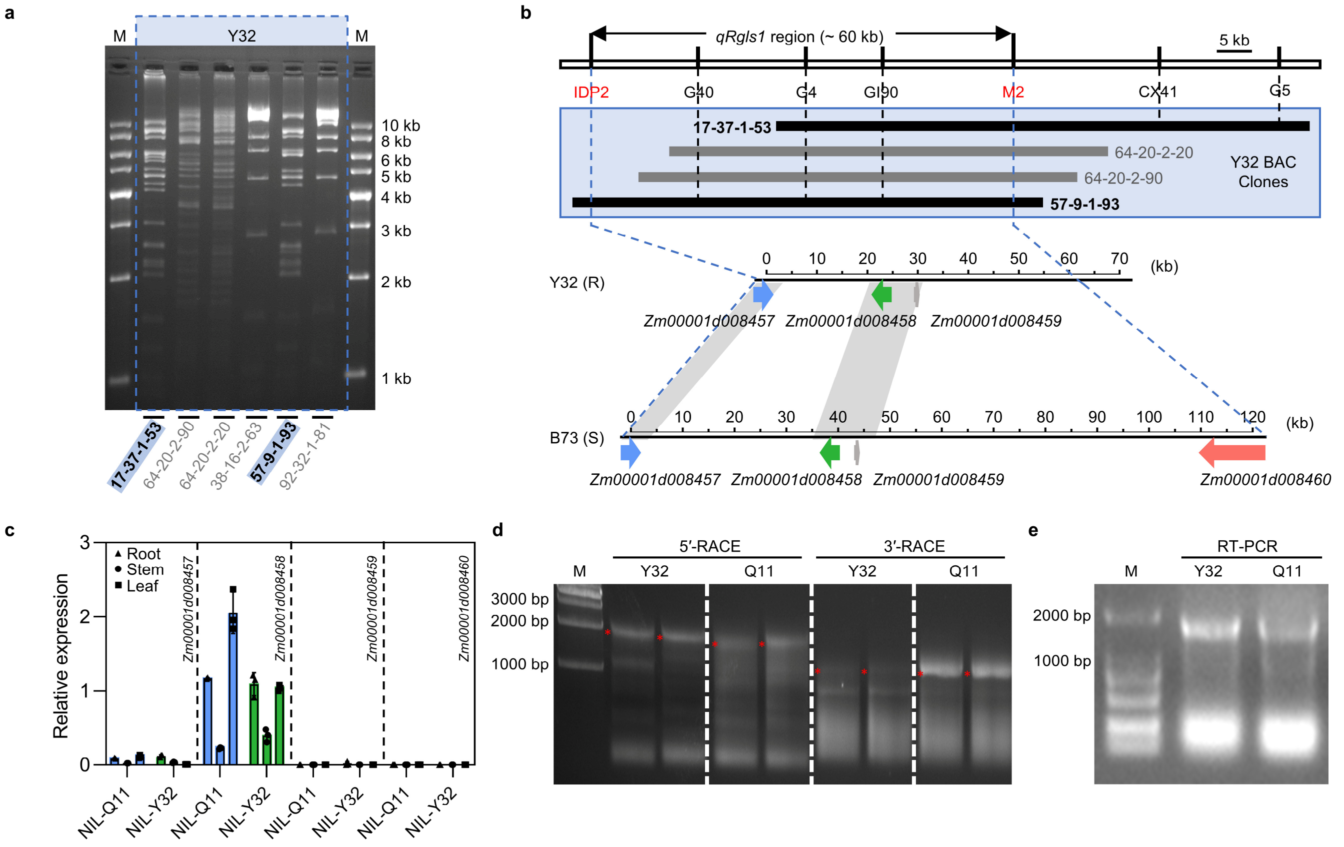

Supplementary Fig. 1: Physical map of the *qRgls1* region and gene structure.

(**a**) The *Bam*HI-digested DNA fingerprints of Y32 BAC clones in the *qRgls1* region. This experiment was repeated three times with similar results.

(**b**) Construction of BAC contig and alignment of predicted protein-coding genes. The arrows indicate predicted genes. Gray shading indicates the syntenic regions between the two inbred lines.

(**c**) Relative expression levels of candidate genes in the roots, stems, and leaves of NIL-Q11 and NIL-Y32 seedlings. Data are shown as means ± s.d. in (**c**) (n=3). Source data is in the Supplementary Data.

(**d**) The full-size transcripts of *ZmWAKL* were obtained by RACE.

(**e**) The full-size cDNA of *ZmPR5L* was generated by RT-PCR. This experiment was repeated two times with the same results.

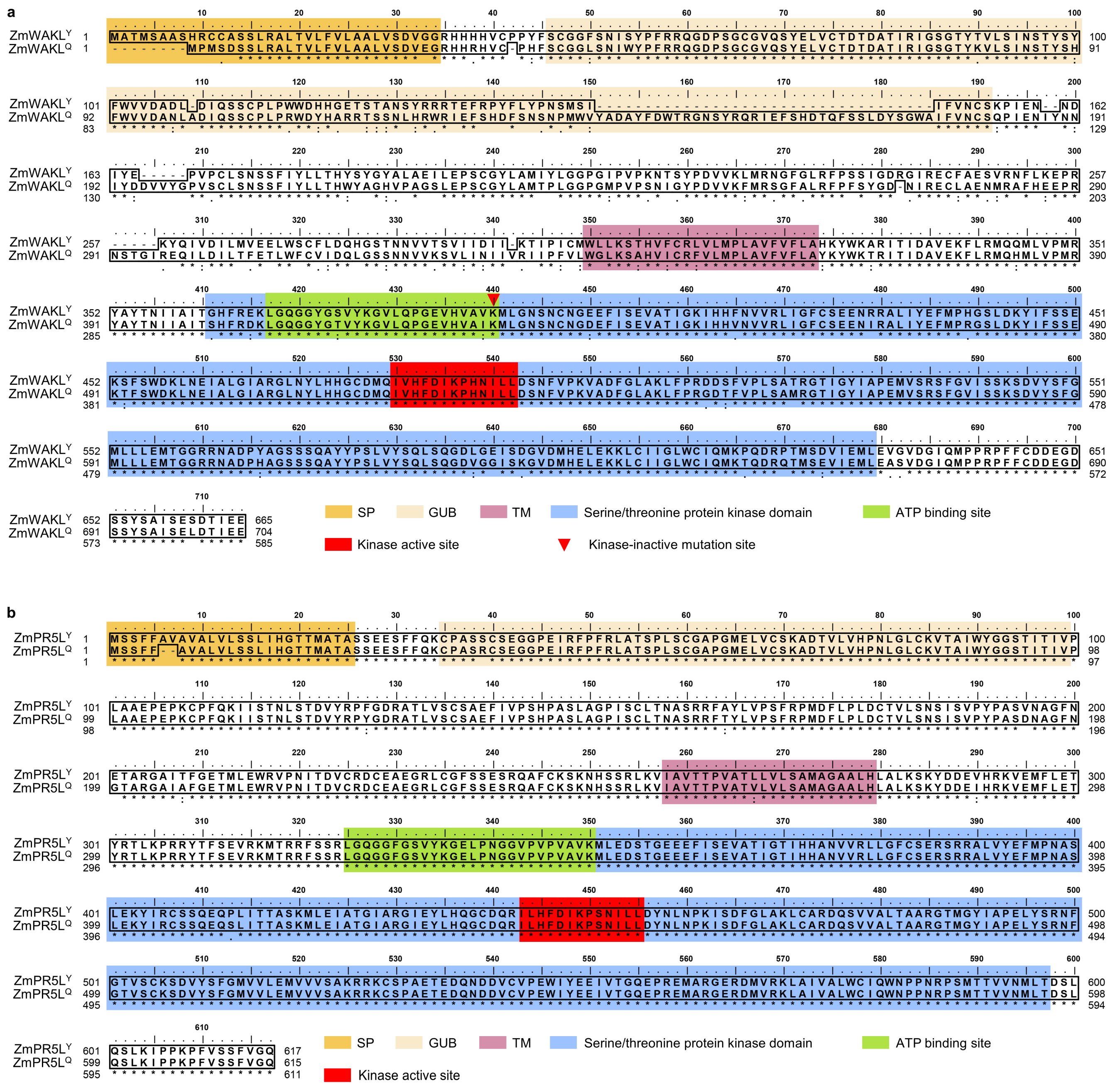

Supplementary Fig. 2: Alignment of amino acid sequences between the Y32 and Q11 alleles of ZmWAKL and ZmPR5L.

(**a**,**b**) Alignment of amino acid sequences between ZmWAKL^{Y} and ZmWAKL^{Q} (**a**) and between ZmPR5L^{Y} and ZmPR5L^{Q} (**b**). The numbers to the left and right indicate the amino acid positions. The red triangle marks the amino-acid site mutated to generate the kinase-inactive ZmWAKL (**a**). SP, signal peptide; GUB, galacturonan-binding domain of wall-associated receptor kinase; TM, transmembrane region.

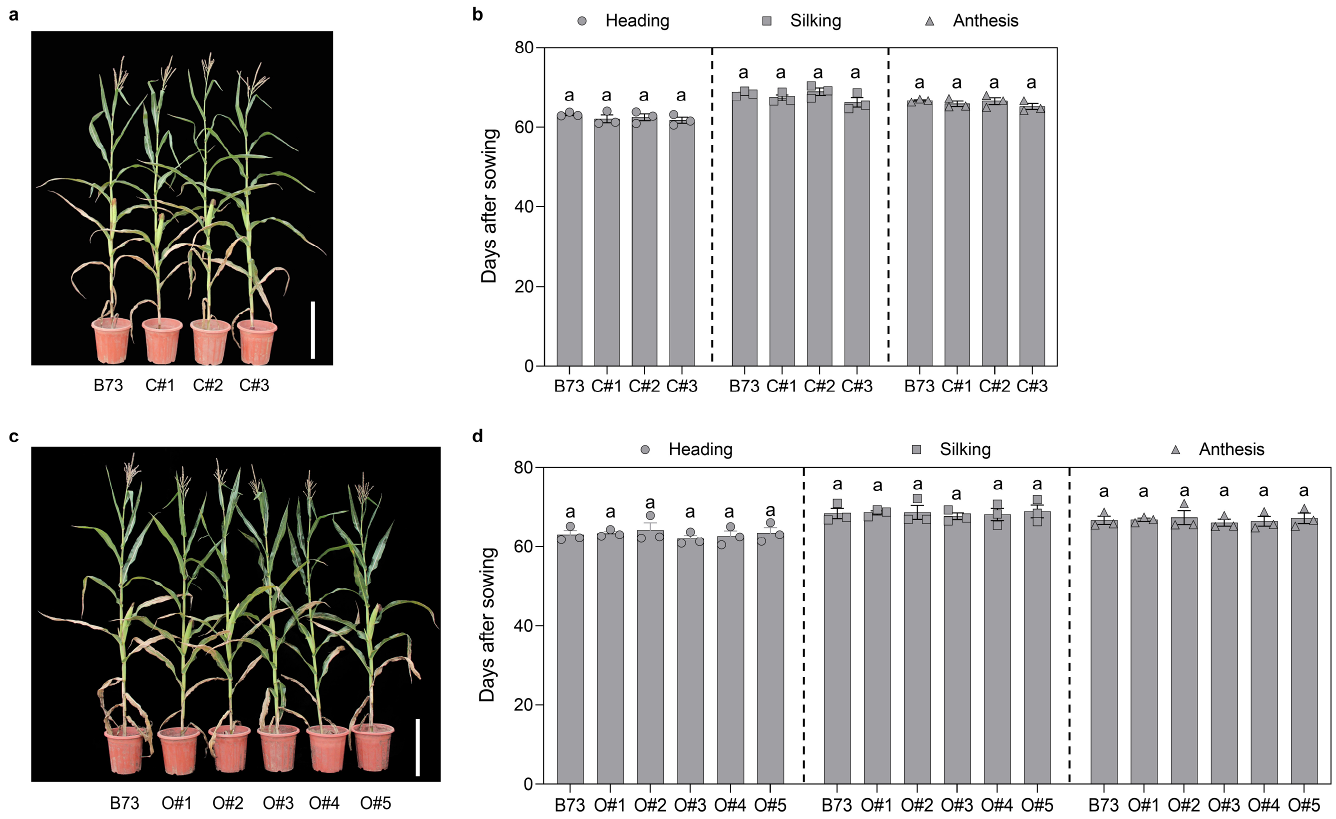

Supplementary Fig. 3: Plant morphology and flowering time of homozygous *ZmWAKL*^{Y} complementation or overexpression transgenic plants.

(**a**,**c**) Plant morphology of B73 and homozygous *ZmWAKL*^{Y} complementation (**a**) and overexpression (**c**) transgenic plants. No significant morphological differences were observed between the transgenic plants and non-transgenic B73. Scale bars, 30 cm.

(**b**,**d**) Flowering-related traits in B73 and homozygous *ZmWAKL*^{Y} complementation (**b**) (n=3) and overexpression (**d**) (n=3) transgenic plants. There were no differences in the three flowering-related traits, days to heading, days to silking, and days to anthesis, between B73 and the transgenic plants grown under long-day conditions in Beijing.

Data in (**b**) and (**d**) are means ± s.e. Different lowercase letters indicate a significant difference ($P < 0.05$), as determined by one-way ANOVA with Tukey's test. Source data is in the Supplementary Data.

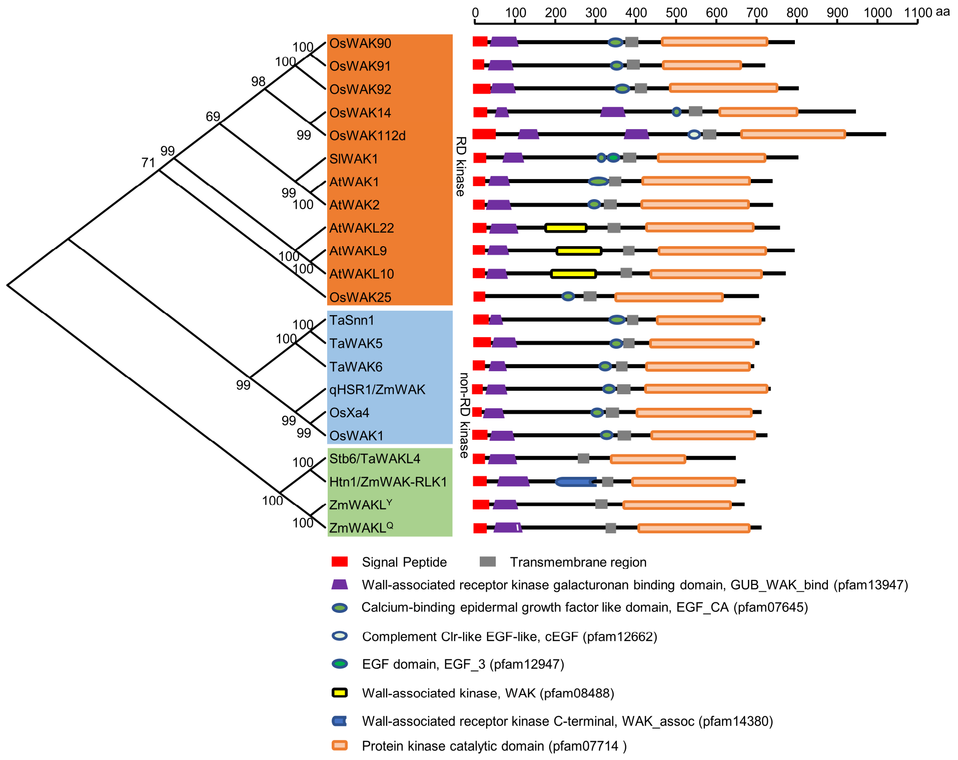

Supplementary Fig. 4: Phylogenetic analysis of ZmWAKL and other WAKs/WAK-like proteins involved in pathogen defense.

The amino acid sequences of reported immune-related WAKs/WAKLs were downloaded from the NCBI database (https://www.ncbi.nlm.nih.gov/). The conserved domains were predicted using the NCBI Conserved Domains Database (https://www.ncbi.nlm.nih.gov/Structure/cdd/wrpsb.cgi). Os, *Oryza sativa*; Sl, *Solanum lycopersicum*; At, *Arabidopsis thaliana*; Ta, *Triticum aestivum*; Zm, *Zea mays*.

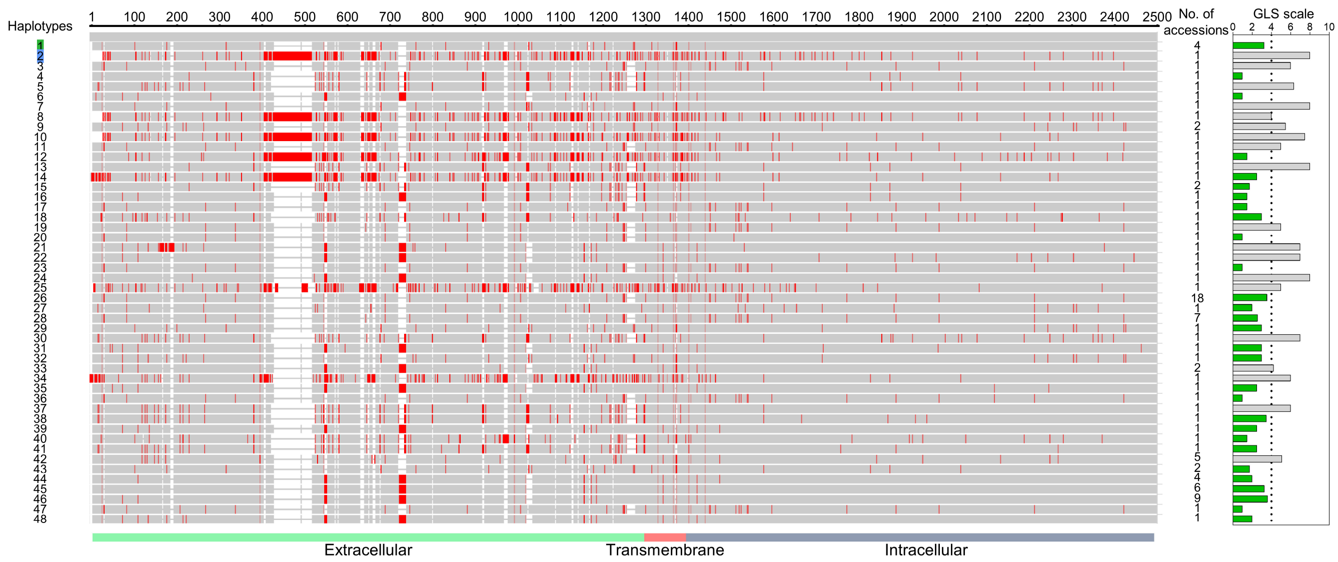

Supplementary Fig. 5: Graphical representation of the alignment of 48 *ZmWAKL* haplotypes.

Single nucleotide polymorphisms and InDels are indicated by red vertical lines and white blocks, respectively. The GLS scale was investigated in the field under natural inoculation and shown to the right. Data are shown as mean. Source data is in the Supplementary Data.

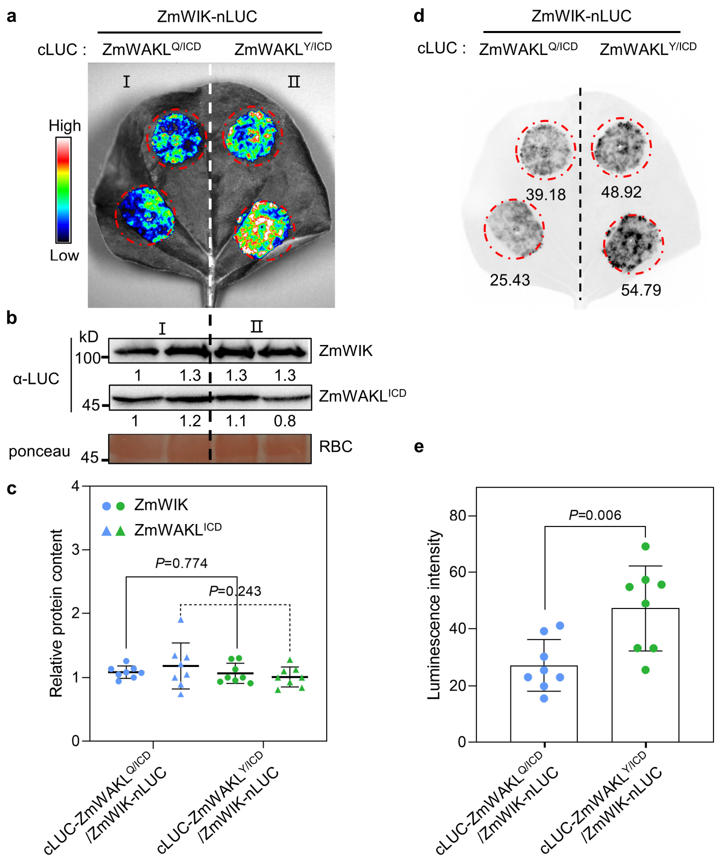

Supplementary Fig. 6: ZmWIK binds more tightly to ZmWAKL$^{Y/ICD}$ than to ZmWAKL$^{Q/ICD}$.

(**a**) Interaction of ZmWIK with ZmWAKL$^{Y/ICD}$ or ZmWAKL$^{Q/ICD}$ in SLC assay.

(**b**,**c**) Comparison of encoded proteins ZmWIK and ZmWAKL^{ICD} between ZmWIK/ZmWAKL$^{Y/ICD}$ and ZmWIK/ZmWAKL$^{Q/ICD}$ (n= 8). The WB assay was repeated two times.

(**d**,**e**) Determination of protein interaction strength based on luminescence intensity (n= 8 independent experiments).

Data are shown as means ± s.d. in (**c**) and (**e**), statistical significance was determined by a two-sided Student's *t*-test. Source data is in the Supplementary Data.

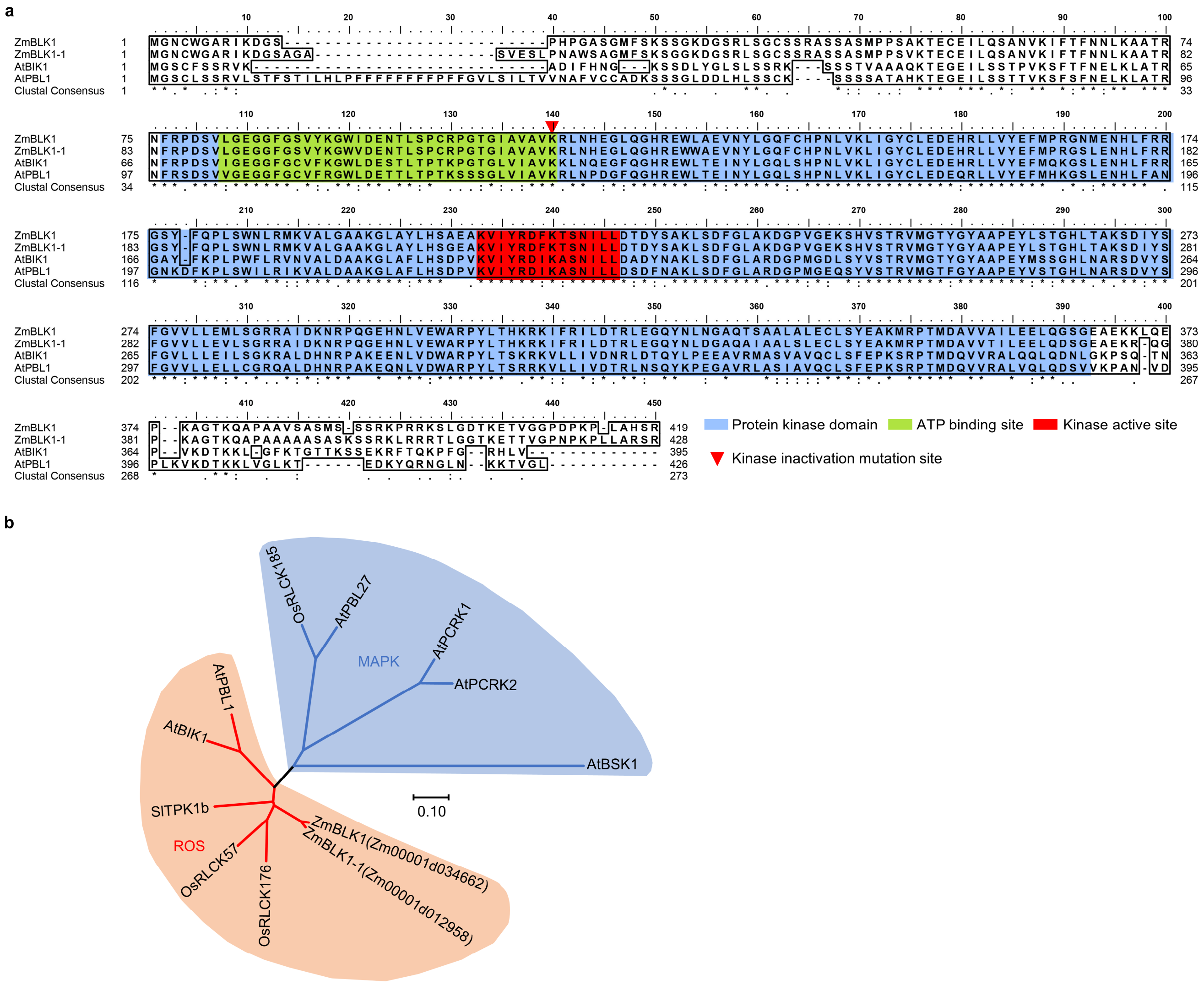

Supplementary Fig. 7: Clustering of receptor-like cytoplasmic kinases (RLCKs) across plant species.

(**a**) Alignment of amino acid sequences among AtBIK1, AtPBL1, and the two homologues of AtBIK1 in the maize B73 genome, ZmBLK1 and ZmBLK1-1. The red triangle marks the mutation site for kinase activity assay.

(**b**) Phylogenetic analysis of RLCKs across plant species. This study included maize RLCKs ZmBLK1 and ZmBLK1-1, as well as other RLCKs collected from literature, including AtPBL1, AtBIK1, SlTPK1b, OsRLCK57, OsRLCK176, OsRLCK185, AtPBL27, AtPCRK1/2 and AtBSK1. Os, *Oryza sativa*; Sl, *Solanum lycopersicum*; At, *Arabidopsis thaliana*; Zm, *Zea mays*.

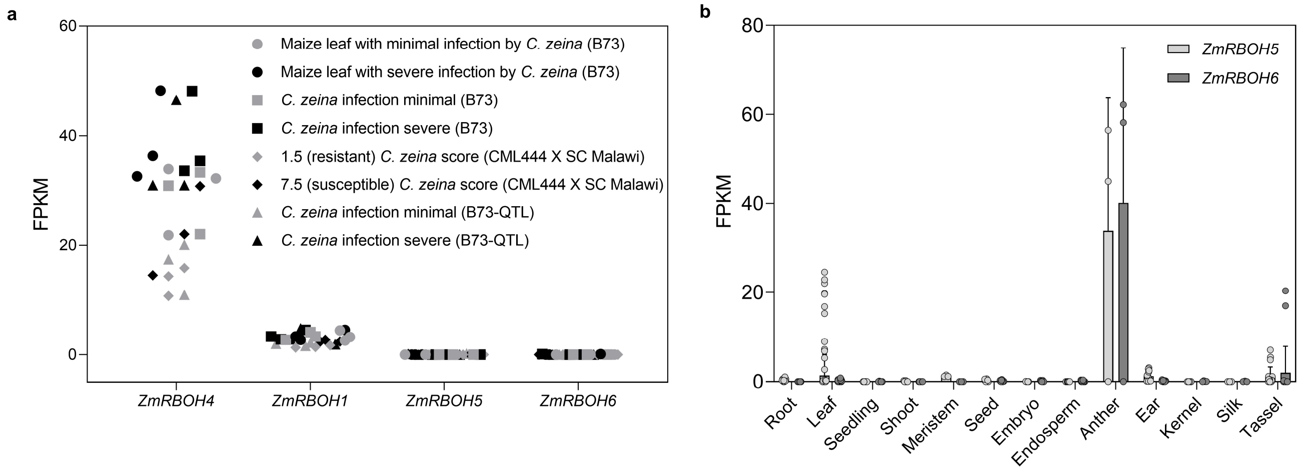

Supplementary Fig. 8: The expression pattern of *ZmRBOHs*.

(**a**) *ZmRBOH4* was expressed at higher levels after *C. zenia* infection compared to *ZmRBOH1*, *ZmRBOH5* and *ZmRBOH6*. The data is obtained from the publicly available RNA-seq database (http://ipf.sustech.edu.cn/pub/plantrna/).

(**b**) *ZmRBOH5* and *ZmRBOH6* are mainly expressed in the anther (n=3). The data comes from the publicly available RNA-seq dataset (http://ipf.sustech.edu.cn/pub/plantrna/).

Data are shown as means ± s.d.. Source data is in the Supplementary Data.

Supplementary Methods

Plant culture and pathogen inoculation in laboratory

Seeds of NILs were sown in 5-cm pots filled with a standard substrate composed of a 1:2 peat-lite/gravel mixture and grown in the chamber under daytime 16h/28°C and dark night 8h/25°C conditions at 60% relative humidity.

The *C. zeina* pathogen was cultured on the maize leaf medium as described previously[1]. The *C. zeina* inoculum was prepared by suspending *C. zeina* spores at a concentration of 5×10^4 spores/ml in a solution containing 0.05% Tween-20. Tween-20 solution alone was used for a mock inoculation. The spray inoculation was carried out at the third fully expanded leaf in each experiment. Approximately 0.5 ml inoculum was evenly sprayed on the leaves. Samples were collected at different time points after inoculation for gene expression analysis or kinase activity assay.

BAC sequencing and gene annotation

The markers in the final mapped *qRgls1* region were used to screen the BAC library constructed from the resistant parental line Y32 (Supplementary Table 1). The Y32 BAC contigs were assembled, and the two overlapping BAC clones were selected for sequencing, gene prediction (http://linux1.softberry.com/berry.phtml), and annotation (https://www.blast2go.com/). The predicted genes were aligned between the Y32 and B73 inbred lines, using the B73 reference sequence as a comparison.

RNA extraction and RT-PCR or RT-qPCR

Total RNAs were isolated from maize leaf samples using the EasyPure Plant RNA kit (TransGen Biotech, China), following the manufacturer's protocol. Complementary DNA (cDNA) was synthesized from 2 μg of total RNA using M-MLV reverse transcriptase (Invitrogen, USA), according to the manufacturer's protocol. cDNA amplification was performed for *ZmPR5L*, *ZmWIK*, *ZmBLK1*, *ZmBLK1-1* and *ZmRBOH4* using Y32, Q11 or NILs. RT-qPCR was carried out on a Rotor-Gene Q 6000 cycler (Corbett Research, UK) with the SYBR Green RT-qPCR kit (Takara Bio, Japan) according to the manufacturer's instructions, with three biological replicates, each with three technical replicates. The maize housekeeping gene *ZmGAPDH* was used as the internal control. The relative expression of the gene was calculated using the $2^{-\Delta\Delta Ct}$ method, as described in the previous study[2].

RACE of $ZmWAKL^{Y}$ and $ZmWAKL^{Q}$

Total RNAs isolated from mature leaves of Y32 and Q11 were reversely transcribed using the SMART RACE cDNA Amplification kit (Clontech, USA) according to the manufacturer's instructions. The 5′ and 3′ gene-specific primers were combined with the universal primer A mix (UPM) to amplify the 5′-cDNA and 3′-cDNA ends, respectively. The 5′-RACE and 3′-RACE products were cloned into the pEasy-T1 vector (TransGen Biotech, China) for sequencing. Sequences from 5′- and 3′-RACE products were assembled to obtain full-length cDNA sequence of *ZmWAKL*.

Sequence and Haplotype analysis

Thirty-day-old leaf samples of 98 inbred lines in the field were harvested or DNA extraction. The DNA was used for amplifying the full-length of *ZmWAKL* by using the TransTaq® DNA Polymerase High Fidelity (TransGen Biotech, # AP131-01). The gel-purified PCR products were quantified and then subjected to Sanger sequencing using an ABI 3730XL platform (ThermoFisher, Switzerland). Primers for PCR amplification are listed in Supplementary Table 1. ClustalX2 was used to produce a nucleotide alignment matrix. Sequence and haplotype analysis was conducted by using software DnaSP6 following the manual. Visualization of haplotypes utilized the online NCBI Multiple Sequence Alignment Viewer (https://www.ncbi.nlm.nih.gov/projects/msaviewer/).

Protein preparation

The intracellular domain of *ZmWAKL*^{Y}, *ZmWAKL*^{Q} and their kinase-inactive mutants, *ZmWAKL*$^{Y/ICD,K391E}$ and *ZmWAKL*$^{Q/ICD,K430E}$, along with the intracellular domain of *ZmWIK*, were amplified and cloned into the MBP-tagged recombinant protein expression vector pETM-40, respectively. The full-length CDS of *ZmBLK1* and its kinase-inactive variant *ZmBLK1*^{K114E}, and *N-ZmRBOH4*, were cloned into the GST-tagged recombinant protein expression vector pGEX-6P-1. The *ZmWAKL*$^{Y/ICD}$, *ZmWAKL*$^{Q/ICD}$, *ZmWIK*^{ICD} and the kinase-inactive variant *ZmWIK*^{ICD,K339E}, and *N-ZmRBOH4* were cloned into the His-tagged recombinant protein expression vectors pHAT2 or pCold-TF, respectively. The plasmids were transformed into *E. coli* BL21 (DE3) strain (TransGen Biotech, # CD601-02). The transformed bacterial preculture was inoculated with 200 mL of lysogeny broth (LB) medium supplemented with 100 µg/mL ampicillin or kanamycin at 37 °C, and incubated until the optical density measured at 600 nm reached 1. The recombinant proteins were then induced with 1 mM isopropyl-β-D-thiogalactoside (IPTG) for 16 hours at 18 °C with shanking at 160 rpm.

For the MBP-tagged recombinant proteins, the bacterial pellet was collected and homogenized in MBP splitting buffer (20 mM Tris-HCl pH 7.4, 0.2 M NaCl, 1 mM EDTA). After sonication and centrifugation at 13,000g at 4 °C, the supernatant was loaded onto a column equipped with MBPSep dextrin agarose resin (YEASEN, #20515ES08), washed with MBP splitting buffer, and eluted with MBP elution buffer (MBP splitting buffer with 10 mM maltose). For the GST-tagged recombinant proteins, the bacterial pellet was collected and homogenized in 1×PBS. After sonication and centrifugation at 13,000g at 4 °C, the supernatant was loaded onto a column equipped with GSTSep glutathione agarose resin (YEASEN, #20507ES10), washed with 1×PBS, and eluted with GST elution buffer (1×PBS with 10 mM glutathinone reduced, pH 8.0). For the His-tagged recombinant proteins, the bacterial pellet was collected and homogenized in His splitting buffer (50 mM Tris-HCl pH 7.5, 300 mM NaCl, 10 mM imidzaole). After sonication and centrifugation at 13,000g at 4 °C, the supernatant was loaded onto a column equipped with HisSep Ni-NTA agarose resin (YEASEN, #20502ES10), washed with washing buffer (50 mM Tris-HCl pH 7.5, 150 mM NaCl, 30 mM imidzaole), and eluted with elution buffer (50 mM Tris-HCl pH 7.5, 150 mM NaCl, 500 mM imidzaole). The purified proteins could be used directly or stored at −80 °C in small aliquots for further experiments.

References for Supplementary Method

1. Lv, X. *et al.* qGLS1.02, a novel major locus for resistance to gray leaf spot in maize. *Mol. Breeding* **40**, 59 (2020).

2. Yang, Q. *et al.* A gene encoding maize caffeoyl-CoA O-methyltransferase confers quantitative resistance to multiple pathogens. *Nat. Genet.* **49**, 1364-1372 (2017).

Supplementary Table 1. Primers used in fine-mapping and studies of molecular mechanism.

	name	sequence (5'-3')	anotation
Primers used in fine-mapping	GZ204	ACGAAGTGGGAAGGGAGA<br>GTGCCTGTGACAGCAACC	
	IDP2	ACCAGATGGCAGTTACCTTA<br>GTAGATGCAACCTCGCTTT	
	IDP11	GTGCCTTGGGTCGTACAATA<br>CCCATCATCACAAGCTCATAGT	dominant
	SSR2	TCTCGAACACACATGAGAGATG<br>TGTAGTTGGGTTTGGATCAGG	dominant
	IDP16	CATATCGCACCCGTGATGTAG<br>GGCTATGCTCTTGCTGAGATT	dominant
	GI90	GGTTGCTTTGCCATGAAGTT<br>CCCATGGACGGTGAAAGTAT	dominant
	M2	ATACACTAAGCCTTCGCACG<br>ACAAGCAGAGGATCATGGTC	
	18-5	GCAGGAGAAATGAGGACA<br>AAGTCAGAGGCATCACAA	
	35-5-3	AAGCACAGAGATGAGACGCT<br>ACGTGTTCCAGCTTCCAGTT	
	SNP2	ATGGGACTGATGCTACTATGT<br>GAGCCACTTCTGCTTTACCT	fragment include a SNP
	IDP5	GAGACAATGAAGGCAGAT<br>TTGTGGACCAACTATGAG	
BAC screen primers	IDP2	ACCAGATGGCAGTTACCTTA<br>GTAGATGCAACCTCGCTTT	
	G40	AGGTATCGCCGAAGGAAGAC<br>ACGTCCTCATGGAGAGCAAT	
	G4	AAGACCGCTCATGTAGAACG<br>CCATCAACATCTCCTTGGCT	
	GI90	GGTTGCTTTGCCATGAAGTT<br>CCCATGGACGGTGAAAGTAT	
	M2	ATACACTAAGCCTTCGCACG<br>ACAAGCAGAGGATCATGGTC	
	CX41	TATGATGTGCATCTGGAAGG<br>TAACGCCCATGCTAAAGACC	
	G5	GATCACCGGCGTCAAGTATT<br>ATTTCAGACCACCCGATCAA	
qRT-PCR primers	*ZmPR5L*	AAATACGACGACGAGATACACA<br>AGAATGTGTACCTTCTCGGTTT	*Zm00001d008457*
	ZmWAKL	CTACATCACGGGTGCGATATG<br>TCGTCTCTTGGGAACAGTTTG	*Zm00001d008458*
	Zm00001d008459	AGGATTCCCGATGTTAAGATCC<br>TCAGTAGGAATTCTCATGACCA	
	Zm00001d008460	TTTGGGATGGACAGTGGATATC<br>GCAGAGAAACATGATATCTGGC	
	ZmWIK	TTGGAAGGGTGTTAGATGTGATAG<br>TCCAATTTCGGGTGGTATGG	*Zm00001d028560*
	ZmGAPDH	ATCAACGGCTTCGGAAGGAT<br>CCGTGGACGGTGTCGTACTT	*Zm00001d049641*
	ZmRBOH4	GAACAAGCTTTCGAAGATCACG<br>CAGGTTGTACAGCTCGATGTAG	*Zm00001d052653*
	ZmRBOH1	CCAAACTGGAGGAAGGTATTCT<br>ATCCTTCAGTTGTTTTGTGAGC	*Zm00001d042961*
	ZmRBOH5	GTCTTCATAGATGGTCCCTACG<br>TAACCCAGTAGAAGTAGGCTCT	*Zm00001d009349*
	ZmRBOH6	CATCATAAAGGCGGCAATCTAC<br>CCAATCACCTAACGTACGGATA	*Zm00001d040974*
RACE primers	GSP5	ATCTCCTTGGCTTAGCTGGCTGTAC	5' RACE
	GSP3	GGCTATGCTCTTGCTGAGATTCTGG	3' RACE
ZmPR5L RT-PCR primers	PR5-L	CATGTCCAGTTTCTTTGCTGT	
	PR5-R	TTACTGGCCAACAAAGGATG	
ZmWAKL genome sequence amplification primers	WAKLFL	ATTATCTTGGTGCTAGCGCG<br>CAAGTCAGCGGCATCACAAT	
complementary transgenic plants screening primers	ComZmWAKL	TTCCTCCTAGCTCCCAACA<br>TGTAAAACGACGGCCAGT	
primers used for chimeric gene construct	$Y^{ECD/TM}$	GACGAGCTC**GGTACC** ATGGCGACGATGTCTGCAGC<br>TTTCCAGTATTTATA GGCTAGGAAGACAAATACTG	
	Q^{ICD}	TATAAATACTGGAAAACACG<br>GACTCTAGA**GGATCC** CTCTTCTATTGTATCCAATT	
	YQ	ATGGCGACGATGTCTGCAGC<br>CTACTCTTCTATTGTATCCA	
overexpression transgenic plants screening primers	Bar	GGTGGACGGCGAGGTCGCCG<br>TCGGTGACGGGCAGGACCGG	
primers used for identification of ZmWIK mutant plants	WIKMu	GCCCAGCTACCTCTTCATTT<br>TCCAATTTCGGGTGGTATGG	
ZmPR5L knock out plants screening primers	PR5CAS	TAGGGCAACCTTAGTGAGC<br>AATGCGATGGCTCCTCTT	
ZmRBOH4 knock out plants screening primers	RBOH4CAS	ATGCATAACCCCAGGCCGGGCGG<br>TCCTGCGACACCTGCTTGAT	
ZmWAKL-GFP construct	WAKLYGFP	TCTGCAGGGGCCCGG **GTCGAC** ATGGCGACGATGTCTGCAGC<br>GCCCTTGCTCACCAT **GGTACC** CTCTTCTATTGTATCCGATT	
	WAKLQGFP	TCTGCAGGGGCCCGG **GTCGAC** ATGCCGATGTCTGATTCTTC<br>GCCCTTGCTCACCAT **GGTACC** CTCTTCTATTGTATCCAATT	

ZmWIK-GFP construct	WIKGFP	TCTGCAGGGGCCCGG **GTCGAC** ATGGGCTCTTTCCTGTGGAAA<br>GCCCTTGCTCACCAT **GGTACC** CTCTGAATCATAGAAGTCGC	
primers used for split-luciferase complementation assay	cLUC-ZmWAKL^{Y}	TACGCGTCCCGGGGC **GGTACC** ATGGCGACGATGTCTGCAGC<br>CCTTGTAGTCCATTTGTT **GGATCC** CTACTCTTCTATTGTATCCG	
	ZmWAKL^{Y}-nLUC	ACGAGCTCGGTACCCG **GGATCC** ATGGCGACGATGTCTGCAGC<br>ACGCGTACGAGATCTG **GTCGAC** CTCTTCTATTGTATCCGATT	
	cLUC-ZmWAKL^{Q}	TACGCGTCCCGGGGC **GGTACC** ATGCCGATGTCTGATTCTTC<br>CCTTGTAGTCCATTTGTT **GGATCC** CTACTCTTCTATTGTATCCA	
	ZmWAKL^{Q}-nLUC	ACGAGCTCGGTACCCG **GGATCC** ATGCCGATGTCTGATTCTTC<br>ACGCGTACGAGATCTG **GTCGAC** CTCTTCTATTGTATCCAATT	
	cLUC-ZmWAKL$^{Y/GUB}$	TACGCGTCCCGGGGC **GGTACC** TCCTGCGGTGGTTTTAGCAA<br>CCTTGTAGTCCATTTGTT **GGATCC** CTA CGAGCAATTCACAAAGATAA	
	ZmWAKL$^{Y/GUB}$-nLUC	ACGAGCTCGGTACCCG **GGATCC** ATG TCCTGCGGTGGTTTTAGCAA<br>ACGCGTACGAGATCTG **GTCGAC** CGAGCAATTCACAAAGATAA	
	cLUC-ZmWAKL$^{Q/GUB}$	TACGCGTCCCGGGGC **GGTACC** TCCTGCGGTGGTTTAAGCAA<br>CCTTGTAGTCCATTTGTT **GGATCC** CTA AGAACAATTCACAAAGATAG	
	ZmWAKL$^{Q/GUB}$-nLUC	ACGAGCTCGGTACCCG **GGATCC** ATG TCCTGCGGTGGTTTAAGCAA<br>ACGCGTACGAGATCTG **GTCGAC** AGAACAATTCACAAAGATAG	
	cLUC-ZmWAKL$^{Y/STK}$	TACGCGTCCCGGGGC **GGTACC** AGAGAAAAGCTCGGACAAGG<br>CCTTGTAGTCCATTTGTT **GGATCC** CTA CTCTATGACGTCGCTCATCG	
	ZmWAKL$^{Y/STK}$-nLUC	ACGAGCTCGGTACCCG **GGATCC** ATG AGAGAAAAGCTCGGACAAGG<br>ACGCGTACGAGATCTG **GTCGAC** CTCTATGACGTCGCTCATCG	
	cLUC-ZmWAKL$^{Q/STK}$	TACGCGTCCCGGGGC **GGTACC** AGAGACAAGCTCGGACAAGG<br>CCTTGTAGTCCATTTGTT **GGATCC** CTA CTCTATGACCTCACTCATCG	
	ZmWAKL$^{Q/STK}$-nLUC	ACGAGCTCGGTACCCG **GGATCC** ATG AGAGACAAGCTCGGACAAGG<br>ACGCGTACGAGATCTG **GTCGAC** CTCTATGACCTCACTCATCG	
	cLUC-ZmWIK	TACGCGTCCCGGGGC **GGTACC** ATGGGCTCTTTCCTGTGGAAAC<br>CCTTGTAGTCCATTTGTT **GGATCC** CTACTCTGAATCATAGAAGT	
	ZmWIK-nLUC	ACGAGCTCGGTACCCG **GGATCC** ATGGGCTCTTTCCTGTGGAAAC<br>ACGCGTACGAGATCTG **GTCGAC** CTCTGAATCATAGAAGTCGC	
	cLUC-ZmWAKL$^{Y/ICD}$	TACGCGTCCCGGGGC **GGTACC** CATAAATACTGGAAAGCAAG<br>CCTTGTAGTCCATTTGTT **GGATCC** CTACTCTTCTATTGTATCCG	
	cLUC-ZmWAKL$^{Q/ICD}$	TACGCGTCCCGGGGC **GGTACC** TATAAATACTGGAAAACACG<br>CCTTGTAGTCCATTTGTT **GGATCC** CTACTCTTCTATTGTATCCA	
	ZmWIK^{ICD}-nLUC	ACGAGCTCGGTACCCG **GGATCC** ATG AAAAATTTTGGAAAAAAAGA<br>ACGCGTACGAGATCTG **GTCGAC** CTCTGAATCATAGAAGTCGC	
	cLUC-ZmWAKL$^{Y/ECD,TM}$	TACGCGTCCCGGGGC **GGTACC** ATGGCGACGATGTCTGCAGC<br>CCTTGTAGTCCATTTGTT **GGATCC** CTA GGCTAGGAAGACAAATACTG	
	cLUC-ZmWAKL$^{Q/ECD,TM}$	TACGCGTCCCGGGGC **GGTACC** ATGCCGATGTCTGATTCTTC<br>CCTTGTAGTCCATTTGTT **GGATCC** CTA GGCTAGGAAGACAAATACTG	
	ZmWIK^{ECD,TM}-nLUC	ACGAGCTCGGTACCCG **GGATCC** ATGGGCTCTTTCCTGTGGAAAC<br>ACGCGTACGAGATCTG **GTCGAC** GTAAAGAAAGCAACCCCAGA	
	cLUC-ZmBLK1	TACGCGTCCCGGGGC **GGTACC** ATGGGGAACTGCTGGGGTGC<br>CCTTGTAGTCCATTTGTT **GGATCC** CTAACGAGAATGGGCCAATG	
	cLUC-ZmBLK1-1	TACGCGTCCCGGGGC **GGTACC** ATGGGGAACTGCTGGGGCGC<br>CCTTGTAGTCCATTTGTT **GGATCC** CTAACGAGAACGGGCCAATA	
	ZmRBOH4-nLUC	ACGAGCTCGGTACCCG **GGATCC** ATGCATAACCCCAGGCCGGG<br>ACGCGTACGAGATCTG **GTCGAC** GAAGTTCTCCTTGTGGAACT	
	N-ZmRBOH4-nLUC	ACGAGCTCGGTACCCG **GGATCC** ATGCATAACCCCAGGCCGGG<br>ACGCGTACGAGATCTG **GTCGAC** GGTCGTGCCGATGCGCACCG	
	N-ZmRBOH1-nLUC	ACGAGCTCGGTACCCG **GGATCC** ATGGCGGGGTACGGGGACCG<br>ACGCGTACGAGATCTG **GTCGAC** GCTCGACGTCGTCCGCTTCA	
	N-ZmRBOH2-nLUC	ACGAGCTCGGTACCCG **GGATCC** ATGAGGGGCGCCGCCGCCCC<br>ACGCGTACGAGATCTG **GTCGAC** GCGTTTCCAATTATCCTCCAA	
	N-ZmRBOH3-nLUC	ACGAGCTCGGTACCCG **GGATCC** ATGCGAGGAGGAGGAGGACC<br>ACGCGTACGAGATCTG **GTCGAC** ACGTTTCCAGTTGTCCTCCA	
	N-ZmRBOH5-nLUC	ACGAGCTCGGTACCCG **GGATCC** ATGGCATCGTCGTCGGGGTA<br>ACGCGTACGAGATCTG **GTCGAC** CCTCTTCCAGTTCTCATGGA	
	N-ZmRBOH6-nLUC	ACGAGCTCGGTACCCG **GGATCC** ATGACATCATCGGCGGGGTA<br>ACGCGTACGAGATCTG **GTCGAC** CTTCCAGTTCTCGTGGACAA	
	ZmRLK1-nLUC	ACGAGCTCGGTACCCG **GGATCC** ATGTCTTCTGCCTTAGTTGC<br>ACGCGTACGAGATCTG **GTCGAC** GTGCACTGATTCTACTGCTG	
	ZmPR5L1-nLUC	ACGAGCTCGGTACCCG **GGATCC** ATGCGACGAGCCGGTGCCGG<br>ACGCGTACGAGATCTG **GTCGAC** ACATGCCACTGAAGCTTGTG	
	ZmPK1-nLUC	ACGAGCTCGGTACCCG **GGATCC** ATGTCGCCTCCTCCGCCCCC<br>ACGCGTACGAGATCTG **GTCGAC** CATGTCCTGCACTAGTCTGG	
	ZmPK2-nLUC	ACGAGCTCGGTACCCG **GGATCC** ATGGGTCGGTGCGGAGGAGG<br>ACGCGTACGAGATCTG **GTCGAC** TTCTGAATCAATTCTAAACC	
	ZmPK3-nLUC	ACGAGCTCGGTACCCG **GGATCC** ATGTGCTCCACTGCAGTGCT<br>ACGCGTACGAGATCTG **GTCGAC** GCGAATCATACTAGATGCTG	
primers used in yeast two hybrid assay	pPR3-N-Y	CAACGCAGAGTGGCCATTACC ATGGCGACGATGTCTGCAGC<br>CGAATTCTCGAGAGGCCGAGG CTACTCTTCTATTGTATCCG	
	pPR3-N-Q	CAACGCAGAGTGGCCATTACC ATGCCGATGTCTGATTCTTC<br>CGAATTCTCGAGAGGCCGAGG CTACTCTTCTATTGTATCCA	
	pBT3-SUC-Y	GGCCATTACGGCC CGACATCATCATCATGTTTG<br>GGCCGAGGCGGCCCC CTCTTCTATTGTATCCGATT	the N-terminal does not contain signal peptide
	pBT3-SUC-Q	GGCCATTACGGCC CGACATCATCGTCATGTCTG<br>GGCCGAGGCGGCCCC CTCTTCTATTGTATCCAATT	
	pGADT7-Y/GUB	GCCATGGAGGCCAGT **GAATTC** TCCTGCGGTGGTTTTAGCAA<br>CAGCTCGAGCTCGAT **GGATCC** CTA CGAGCAATTCACAAAGATAA	
	pGBKT7-Y/GUB	ATGGCCATGGAGGCC **GAATTC** TCCTGCGGTGGTTTTAGCAA<br>CCGCTGCAGGTCGAC **GGATCC** CTA CGAGCAATTCACAAAGATAA	
	pGADT7-Q/GUB	GCCATGGAGGCCAGT **GAATTC** TCCTGCGGTGGTTTAAGCAA<br>CAGCTCGAGCTCGAT **GGATCC** CTA AGAACAATTCACAAAGATAG	
	pGBKT7-Q/GUB	ATGGCCATGGAGGCC **GAATTC** TCCTGCGGTGGTTTAAGCAA<br>CCGCTGCAGGTCGAC **GGATCC** CTA AGAACAATTCACAAAGATAG	

	pGADT7-Y/STK	GCCATGGAGGCCAGT **GAATTC** AGAGAAAAGCTCGGACAAGG<br>CAGCTCGAGCTCGAT **GGATCC** CTA CTCTATGACGTCGCTCATCG
	pGBKT7-Y/STK	ATGGCCATGGAGGCC **GAATTC** AGAGAAAAGCTCGGACAAGG<br>CCGCTGCAGGTCGAC **GGATCC** CTA CTCTATGACGTCGCTCATCG
	pGADT7-Q/STK	GCCATGGAGGCCAGT **GAATTC** AGAGACAAGCTCGGACAAGG<br>CAGCTCGAGCTCGAT **GGATCC** CTA CTCTATGACCTCACTCATCG
	pGBKT7-Q/STK	ATGGCCATGGAGGCC **GAATTC** AGAGACAAGCTCGGACAAGG<br>CCGCTGCAGGTCGAC **GGATCC** CTA CTCTATGACCTCACTCATCG
primers used in Co-IP assay	WAKL^{Y}MYC	TCTGCAGGGGCCCGG **GTCGAC** ATGGCGACGATGTCTGCAGC<br>GAGCTTTTGCTCCAT **GGTACC** CTCTTCTATTGTATCCGATT
	WAKL^{Q}MYC	TCTGCAGGGGCCCGG **GTCGAC** ATGCCGATGTCTGATTCTTC<br>GAGCTTTTGCTCCAT **GGTACC** CTCTTCTATTGTATCCAATT
	WIKMYC	TCTGCAGGGGCCCGG **GTCGAC** ATGGGCTCTTTCCTGTGGAAA<br>GAGCTTTTGCTCCAT **GGTACC** CTCTGAATCATAGAAGTCGC
	BLK1MYC	TCTGCAGGGGCCCGG **GTCGAC** ATGGGGAACTGCTGGGGTGC<br>GAGCTTTTGCTCCAT **GGTACC** ACGAGAATGGGCCAATGGTT
	WIK^{ECD,TM}GFP	TCTGCAGGGGCCCGG **GTCGAC** ATGGGCTCTTTCCTGTGGAAA<br>GCCCTTGCTCACCAT **GGTACC** GTAAAGAAAGCAACCCCAGA
	WIK^{ICD}GFP	TCTGCAGGGGCCCGG **GTCGAC** ATG AAAAATTTTGGAAAAAAAGA<br>GCCCTTGCTCACCAT **GGTACC** CTCTGAATCATAGAAGTCGC
primers used in prokaryotic expression	ZmWAKL$^{Y/ICD}$-MBP	TTTATTTTCAGGGCG **CCATGG** CC CATAAATACTGGAAAGCAAG<br>TTGTCGACGGAGCTC **GAATTC** CTACTCTTCTATTGTATCCG
	ZmWAKL$^{Y/ICD,K391E}$-MBP	TGCCACTGAGCGCTA**t**GCGGGGAACG<br>**a**TAGCGCTCAGTGGCACGAAACTGT
	ZmWAKL$^{Q/ICD}$-MBP	TTTATTTTCAGGGCG **CCATGG** CC TATAAATACTGGAAAACACG<br>TTGTCGACGGAGCTC **GAATTC** CTACTCTTCTATTGTATCCA
	ZmWAKL$^{Q/ICD,K430E}$-MBP	TGCCACTGAGCGCTA**c**GCGGGGAACG<br>**g**TAGCGCTCAGTGGCACGAAAGTGT
	His-TF-ZmWAKL$^{Y/ICD}$	AGGCATATGGAGCTC **GGTACC** CATAAATACTGGAAAGCAAG<br>GACAAGCTTGAATTC **GGATCC** CTACTCTTCTATTGTATCCG
	His-TF-ZmWAKL$^{Q/ICD}$	AGGCATATGGAGCTC **GGTACC** TATAAATACTGGAAAACACG<br>GACAAGCTTGAATTC **GGATCC** CTACTCTTCTATTGTATCCA
	WIK^{ICD}-MBP	TTTATTTTCAGGGCG **CCATGG** CC AAAAATTTTGGAAAAAAAGA<br>TTGTCGACGGAGCTC **GAATTC** CTACTCTGAATCATAGAAGT
	His-ZmWAKL$^{Y/ICD}$	ACCATCACCATCACT **CCATGG** AT CATAAATACTGGAAAGCAAG<br>TAGAGGGCCCGGATCC **CTCGAG** CTACTCTTCTATTGTATCCG
	His-ZmWAKL$^{Q/ICD}$	ACCATCACCATCACT **CCATGG** AT TATAAATACTGGAAAACACG<br>TAGAGGGCCCGGATCC **CTCGAG** CTACTCTTCTATTGTATCCA
	His-WIK^{ICD}	ACCATCACCATCACT **CCATGG** AT AAAAATTTTGGAAAAAAAGA<br>TAGAGGGCCCGGATCC **CTCGAG** CTACTCTGAATCATAGAAGT
	His-WIK^{ICD,K339E}	AATGTCTTTGCTTTG**g**AAAGGATAGT<br>**c**CAAAGCAAAGACATTGCCATCATC
	BLK1-GST	TTCCAGGGGCCCCTG **GGATCC** ATGGGGAACTGCTGGGGTGC<br>CTCGAGTCGACCCGG **GAATTC** CTAACGAGAATGGGCCAATG
	BLK1^{K114E}-GST	ATCGCTGTTGCTGTG**g**AAAGACTCAA<br>**c**CACAGCAACAGCGATTCCAGTGCC
	N-RBOH4-MBP	TTTATTTTCAGGGCG **CCATGG** CC ATGCATAACCCCAGGCCGGG<br>TTGTCGACGGAGCTC **GAATTC** CTA GGTCGTGCCGATGCGCACCG
	His-N-RBOH4	ACCATCACCATCACT **CCATGG** AT ATGCATAACCCCAGGCCGGG<br>TAGAGGGCCCGGATCC **CTCGAG** TCA GGTCGTGCCGATGCGCACCG
	N-RBOH4-GST	TTCCAGGGGCCCCTG **GGATCC** ATGCATAACCCCAGGCCGGG<br>CTCGAGTCGACCCGG **GAATTC** CTA GGTCGTGCCGATGCGCACCG

Supplementary Table 2. Maize inbred lines used in this study.

No.	Accessions	GLS scale	*ZmWAKL* haplotypes
1	YML32	1.57	Hap-26
2	DTMA-199	4.14	Hap-45
3	DTMA-4	6.00	Hap-28
4	DTMA-15	2.00	Hap-28
5	DTMA-16	2.50	Hap-28
6	DTMA-23	6.00	Hap-3
7	DTMA-26	2.00	Hap-26
8	DTMA-27	1.00	Hap-4
9	B73	9.00	Hap-9
10	DTMA-46	6.33	Hap-5
11	DTMA-49	1.00	Hap-6
13	DTMA-76	8.00	Hap-7
14	DTMA-80	1.00	Hap-26
15	DTMA-83	4.00	Hap-8
16	Mo17	2.00	Hap-9
17	DTMA-95	9.00	Hap-26
18	DTMA-97	2.00	Hap-28
19	DTMA-98	3.50	Hap-28
22	DTMA-121	7.50	Hap-10
23	DTMA-122	5.00	Hap-11
24	DTMA-124	2.50	Hap-46
26	DTMA-132	1.50	Hap-12
27	DTMA-133	1.00	Hap-44
28	HZ4	9.00	Hap-46
29	DTMA-141	3.00	Hap-44
30	DTMA-151	2.50	Hap-45
32	DTMA-161	1.00	Hap-45
33	DTMA-162	8.00	Hap-13
34	DTMA-165	2.50	Hap-14
35	DTMA-173	2.50	Hap-15
36	DTMA-180	1.00	Hap-46
37	DTMA-189	2.00	Hap-43
38	DTMA-193	1.50	Hap-16
39	DTMA-194	1.50	Hap-17
40	DTMA-205	3.00	Hap-18
41	DTMA-216	2.50	Hap-26
42	1145	2.00	Hap-1
43	DTMA-219	5.00	Hap-19
44	DTMA-221	1.00	Hap-20
45	DTMA-224	1.00	Hap-28
46	DTMA-225	7.00	Hap-21
47	DTMA-226	1.00	Hap-1
49	DTMA-231	7.00	Hap-22
50	DTMA-237	5.00	Hap-45
51	DTMA-238	4.00	Hap-45
52	DTMA-239	1.00	Hap-26
53	DTMA-241	1.00	Hap-23
55	DTMA-244	8.00	Hap-24

56	DTMA-256	5.00	Hap-25
57	DTMA-259	4.00	Hap-26
58	DTMA-261	2.00	Hap-27
59	DTMA-273	1.00	Hap-28
60	DTMA-274	3.00	Hap-29
61	DTMA-281	5.00	Hap-26
62	DTMA-287	7.00	Hap-30
63	DTMA-289	3.00	Hap-26
64	DTMA-294	3.00	Hap-31
65	Zheng 58	3.00	Hap-32
66	DTMA-299	5.00	Hap-26
67	(P147-F2-102-S6/P33-C3-64-S4)-F2-B-24-1-1	5.50	Hap-26
68	(P147-F2-102-S6/P33-C3-64-S4)-F2-B-24-2-1	4.50	Hap-33
69	(CML323/CML226)F2-28-3-B-1	6.00	Hap-46
70	(CML329/CML20)F2-47-2-B-4	4.00	Hap-33
71	(CML226/(CATETO DC1276/7619))F2-5-1-B-1	6.00	Hap-34
72	(CML226/(CATETO DC1276/7619))F2-25-1-B-1	3.00	Hap-26
73	(CML226/(CATETO DC1276/7619))F2-25-1-B-3	2.00	Hap-26
74	(CML226/(CATETO DC1276/7619))F2-38-3-B-1	5.00	Hap-46
76	(CML226/(CATETO DC1276/7619))F2-38-3-B-3	5.00	Hap-46
78	(CML226/CATETO//CML226/CATETO)F2-B-1-2-B	2.50	Hap-35
79	P45-C6-FS40-1-1-1-B-1-1-3-2-1-1-2-B	1.00	Hap-36
80	(P147-F2-114-S7/P45-C8-76-S9)-F2-B-2-1-1	5.00	Hap-42
81	(P147-F2-136-S7/P45-C8-76-S9)-F2-B-15-1-3	7.00	Hap-42
82	(P147-F2-136-S7/P45-C8-76-S9)-F2-B-15-2-1	5.50	Hap-42
83	(P147-F2-152-S7/P45-C8-76-S9)-F2-B-13-1-1	3.00	Hap-42
84	GLSIY01HGA-B-8-1-1-B	6.00	Hap-37
85	(FS8BT-278-B-6-1-4-2-3-1-B/MD37)-8-B-32-1-B*3-3-B	3.50	Hap-38
86	CLA37 = SA5-C2-FS(26/21)-4-1-5-6-B*4	1.00	Hap-26
88	CLA155 = SA3-C4-FS(16/25)-2-4-3-1-4-5-2	5.00	Hap-26
89	CLA161 = SA4-C2-FS(21/26)-1-2-2-2-2-1-2	9.00	Hap-1
90	CL-RCY023 = (CL-02439/CML-286)-B-1-2-2-B*8	2.50	Hap-39
91	CL-02725 = P27(FRRS)C1-248-B-1-B*4	1.50	Hap-40
92	P33-C4-F2-15-4-2-2-B	2.50	Hap-41
93	P45-C8-164-1-1-2-8-B	5.00	Hap-42
94	Cel FSR/MBRYS9954-B-32-1-1-B	1.00	Hap-15
95	Q11	8.00	Hap-2
96	Y32	1.00	Hap-1
97	(CML329/MBRY-C2)-F7-3-B	4.00	Hap-26
98	(CML323/(CATETO DC1276/7619))-F2-42-2-B-1	1.00	Hap-46
99	(CML323/(CATETO DC1276/7619))-F2-42-2-B-2	3.00	Hap-26
100	P502-C3-F2-13-4-2-1-B	1.00	Hap-44
101	CML 199, MSR-76-1-B*3-3-3-B	1.50	Hap-43
102	CML256, (P21-F38/P21-F114)-2-1-2-1-B	3.00	Hap-44
103	CML306, SINTAMTSR-19-1-2-3-1-B	3.00	Hap-45
104	YML171	1.00	Hap-46
105	CML 165	2.00	Hap-46
106	CML 273	1.00	Hap-47
108	CML390	2.00	Hap-48
109	CML463	6.50	Hap-26

Supplementary Table 3. Statistical analysis of 98 *ZmWAKL* sequences.

Fragments	Fragment length (bp)[a]	No. of sites (bp)[b]	No. of haplotypes	*H*	*π*	Tajima's *D*	*D*	*F*
Extracellular sequence[c]	1457	1187	38	0.988	0.07349	-0.58574	-1.19113	-1.15176
Intracellular sequence	1026	1026	29	0.952	0.02119	-1.35149	-2.02063	-2.11687

Sequence analysis was conducted using software DnaSP6. [a] The fragment length indicates the length of $ZmWAKL^{Y}$. [b] Sites with gaps and missing data were omitted for analysis. [c] The extracellular sequence includes the sequence of the transmembrane domain. *H*, haplotype diversity; *π*, nucleotide diversity; *D*, Fu and Li's D test; *F*, Fu and Li's F test.

Supplementary Table 4. The IP-MS results of ZmWAKL^{Y}-GFP.

Receptor like kinase

**No.**	**Accession**	**Description**	**Name**	**TM (Yes/No)**	**Kinase (Yes/No)**	***Rep 1* (Yes/No)**	***Rep 2* (Yes/No)**	**Leaf expression (FPKM)[a]**	***C.zeina* (FPKM)[a]**
	Zm00001d008458_P001	Cell-wall-associated receptor kinase-like protein	ZmWAKL	Y	Y	Y	Y	11.55	28.33
1	Zm00001d039243_P001	Receptor-like serine/threonine-protein kinase	ZmRLK1	Y	Y	N	Y	1.47	1.14
2	Zm00001d028560_P001	LRR receptor-like serine/threonine-protein kinase FEI 1	ZmWIK	Y	Y	Y	Y	26.26	3.39
3	Zm00001d039921_P001	PR5-like receptor kinase	ZmPR5L1	Y	Y	N	Y	1.95	0.39
4	Zm00001d010178_P001	Protein kinase superfamily protein	ZmPK1	Y	Y	N	Y	24.59	0
5	Zm00001d011700_P014	Protein kinase superfamily protein	ZmPK2	Y	Y	N	Y	14.83	2.05
6	Zm00001d031351_P001	Protein kinase superfamily protein	ZmPK3	Y	Y	N	Y	6.37	9.48

[a]Transcriptome data get from public databases (http://ipf.sustech.edu.cn/pub/plantrna/).

Repetition 1

**Family**	**Member**	**Database**	**Accession**	**Score**	**Mass**	**Num. of matches**	**Num. of significant matches**	**Num. of sequences**	**Num. of significant sequences**	**Description**
1	1	Zea_mays_GDB	Zm00001d000279_P001	1147	54128	51	51	10	10	Ribulose bisphosphate carboxylase large chain
2	1	Zea_mays_GDB	Zm00001d027511_P001	1029	56705	46	46	8	8	Catalase isozyme 2
2	2	Zea_mays_GDB	Zm00001d014848_P001	834	57233	35	35	4	4	Catalase isozyme 1
3	1	Zea_mays_GDB	Zm00001d008458_P002	638	75685	33	33	10	10	Cell-wall-associated receptor-like protein kinase
4	1	Zea_mays_GDB	Zm00001d000302_P001	439	31189	10	10	3	3	Probable protein phosphatase 2C 59
4	2	Zea_mays_GDB	Zm00001d001988_P001	222	34798	8	8	3	3	Probable protein phosphatase 2C 59
5	1	Zea_mays_GDB	Zm00001d037875_P001	433	49492	17	17	8	8	elongation factor alpha2
6	1	Zea_mays_GDB	Zm00001d009504_P001	425	73189	23	23	8	8	Dynamin protein 1A
6	2	Zea_mays_GDB	Zm00001d030005_P002	274	45829	11	11	5	5	Dynamin-related protein 1E
6	3	Zea_mays_GDB	Zm00001d013426_P001	252	66313	16	16	6	6	Dynamin-related protein 1C
7	1	Zea_mays_GDB	Zm00001d041550_P004	393	71517	15	15	8	8	Heat shock 70 kDa protein 3
7	2	Zea_mays_GDB	Zm00001d010529_P001	301	71311	12	12	7	7	Probable mediator of RNA polymerase II transcription subunit 37c
7	3	Zea_mays_GDB	Zm00001d012420_P001	286	71180	12	12	7	7	Heat shock 70 kDa protein
7	4	Zea_mays_GDB	Zm00001d014993_P001	87	73211	5	5	4	4	Luminal-binding protein 2
8	1	Zea_mays_GDB	Zm00001d000277_P001	386	61515	16	16	5	5	ATP synthase subunit beta chloroplastic
8	2	Zea_mays_GDB	Zm00001d009488_P001	105	59085	5	5	2	2	ATP synthase subunit beta
9	1	Zea_mays_GDB	Zm00001d018810_P001	309	45430	13	13	3	3	glycolate oxidase1
10	1	Zea_mays_GDB	Zm00001d000270_P009	253	55713	9	9	6	6	DNA-directed RNA polymerase subunit beta
10	2	Zea_mays_GDB	ZeamMp163	104	55431	8	8	5	5	atp1-a1
11	1	Zea_mays_GDB	ZemaCp008	247	52038	8	8	5	5	psbC
12	1	Zea_mays_GDB	Zm00001d003429_P001	233	43172	10	10	5	5	glyceraldehyde-3-phosphate dehydrogenase1
13	1	Zea_mays_GDB	Zm00001d023559_P001	183	41896	5	5	2	2	Fructose-bisphosphate aldolase
14	1	Zea_mays_GDB	Zm00001d032187_P001	179	41373	3	3	1	1	Malate dehydrogenase
15	1	Zea_mays_GDB	Zm00001d000260_P001	153	39762	5	5	2	2	Photosystem II D2 protein
16	1	Zea_mays_GDB	Zm00001d013410_P001	147	41998	8	8	6	6	Actin-1
17	1	Zea_mays_GDB	Zm00001d053765_P001	141	68689	8	8	5	5	vacuolar proton pump3
18	1	Zea_mays_GDB	Zm00001d013777_P001	127	74573	3	3	2	2	MYB-related-transcription factor 40
19	1	Zea_mays_GDB	Zm00001d037279_P001	117	98261	9	9	5	5	Dynamin-2A
20	1	Zea_mays_GDB	Zm00001d039658_P001	110	25720	4	4	1	1	bZIP-transcription factor 6
21	1	Zea_mays_GDB	Zm00001d005334_P001	109	14977	5	5	3	3	Ubiquitin-60S ribosomal protein L40-1
22	1	Zea_mays_GDB	Zm00001d020256_P001	94	63289	5	5	1	1	P-loop containing nucleoside triphosphate hydrolases superfamily protein
23	1	Zea_mays_GDB	Zm00001d045451_P001	93	79444	3	3	3	3	transketolase 1
24	1	Zea_mays_GDB	Zm00001d049239_P001	89	53884	4	4	2	2	adenosyl homocysteine hydrolase1
25	1	Zea_mays_GDB	Zm00001d005090_P008	85	195605	5	5	5	5	Clathrin heavy chain 2
26	1	Zea_mays_GDB	Zm00001d012846_P001	85	48658	5	5	2	2	Elongation factor Tu mitochondrial
27	1	Zea_mays_GDB	Zm00001d036961_P003	83	36924	2	2	2	2	Putative ATPase%2C V1 complex%2C subunit B protein isoform 1%3B
28	1	Zea_mays_GDB	Zm00001d005789_P001	83	16353	3	3	2	2	histone 2B3
29	1	Zea_mays_GDB	Zm00001d005485_P001	81	32059	2	2	2	2	prohibitin1
30	1	Zea_mays_GDB	Zm00001d014564_P001	80	39337	1	1	1	1	oxygen-evolving complex 33 kda protein b
31	1	Zea_mays_GDB	Zm00001d044903_P005	77	141911	5	5	3	3	Kinesin-like protein KCA2
32	1	Zea_mays_GDB	Zm00001d052698_P001	73	28687	4	4	3	3	14-3-3-like protein GF14 nu
33	1	Zea_mays_GDB	Zm00001d017857_P001	73	42593	5	5	3	3	adenine nucleotide translocator1
34	1	Zea_mays_GDB	Zm00001d017121_P001	72	55925	4	4	3	3	glyceraldehyde-3-phosphate dehydrogenase4
34	2	Zea_mays_GDB	Zm00001d015383_P001	42	44660	2	2	2	2	Glyceraldehyde-3-phosphate dehydrogenase%2C cytosolic
35	1	Zea_mays_GDB	Zm00001d028560_P001	72	46728	4	4	2	2	LRR receptor-like serine/threonine-protein kinase FEI1
36	1	Zea_mays_GDB	Zm00001d002450_P001	71	17821	3	3	1	1	60S ribosomal protein L12-3
37	1	Zea_mays_GDB	Zm00001d022111_P001	70	26026	1	1	1	1	40S ribosomal protein S3-1
38	1	Zea_mays_GDB	Zm00001d041819_P001	70	15704	3	3	1	1	photosystem I N subunit1
39	1	Zea_mays_GDB	Zm00001d005944_P001	70	21107	7	7	1	1	
40	1	Zea_mays_GDB	Zm00001d015886_P001	68	46865	6	6	3	3	26S protease regulatory subunit 6B homolog
41	1	Zea_mays_GDB	Zm00001d014899_P001	67	67829	3	3	1	1	
42	1	Zea_mays_GDB	Zm00001d053630_P001	64	25155	2	2	2	2	ribosomal protein S8 homolog
43	1	Zea_mays_GDB	Zm00001d015094_P001	64	60090	2	2	1	1	Alkaline/neutral invertase CINV2
44	1	Zea_mays_GDB	Zm00001d003083_P001	61	54928	1	1	1	1	Isocitrate dehydrogenase
45	1	Zea_mays_GDB	Zm00001d006540_P001	60	23037	3	3	1	1	Oxygen-evolving enhancer protein 3-1
46	1	Zea_mays_GDB	Zm00001d008387_P001	60	22467	2	2	2	2	ribosomal protein S5
47	1	Zea_mays_GDB	Zm00001d006008_P001	60	80407	2	2	2	2	Heat shock protein 90-2
48	1	Zea_mays_GDB	Zm00001d007900_P002	59	36379	3	3	1	1	Eukaryotic translation initiation factor 3 subunit I
49	1	Zea_mays_GDB	Zm00001d039038_P001	59	65308	4	4	1	1	Eukaryotic translation initiation factor 3 subunit D
50	1	Zea_mays_GDB	Zm00001d010056_P001	59	78287	2	2	2	2	proline responding1 description
51	1	Zea_mays_GDB	Zm00001d042697_P001	58	28434	1	1	1	1	photosystem II subunit PsbS1
52	1	Zea_mays_GDB	Zm00001d035925_P001	57	55248	2	2	2	2	6-phosphogluconate dehydrogenase1
53	1	Zea_mays_GDB	Zm00001d003680_P001	55	44892	3	3	2	2	60S ribosomal protein L3-1
54	1	Zea_mays_GDB	Zm00001d017092_P001	55	50790	1	1	1	1	Elongation factor Tu
55	1	Zea_mays_GDB	Zm00001d033132_P001	54	27948	3	3	2	2	photosystem II light harvesting complex gene 2.1
56	1	Zea_mays_GDB	Zm00001d006871_P001	54	33179	4	4	1	1	40S ribosomal protein Sa-1
57	1	Zea_mays_GDB	Zm00001d052923_P001	50	57805	1	1	1	1	Patatin-like protein 3
58	1	Zea_mays_GDB	Zm00001d006923_P002	50	22560	6	6	3	3	40S ribosomal protein S9-2
58	2	Zea_mays_GDB	Zm00001d029340_P001	25	9734	2	2	2	2	40S ribosomal protein S9-2
59	1	Zea_mays_GDB	Zm00001d040294_P001	49	15789	2	2	1	1	Sm-like protein LSM4
60	1	Zea_mays_GDB	Zm00001d012770_P001	49	94872	4	4	4	4	Putative translation elongation factor family protein
61	1	Zea_mays_GDB	Zm00001d035212_P001	48	35994	3	3	2	2	Chloroplast stem-loop binding protein of 41 kDa b chloroplastic
62	1	Zea_mays_GDB	Zm00001d003127_P001	47	16930	1	1	1	1	60S ribosomal protein L23a-1
63	1	Zea_mays_GDB	Zm00001d031349_P001	44	44363	1	1	1	1	Serine--glyoxylate aminotransferase
64	1	Zea_mays_GDB	Zm00001d002735_P002	44	26234	1	1	1	1	40S ribosomal protein S7-2
65	1	Zea_mays_GDB	Zm00001d000104_P001	43	12089	1	1	1	1	40S ribosomal protein S14-3
66	1	Zea_mays_GDB	Zm00001d039922_P001	42	20690	3	3	1	1	
67	1	Zea_mays_GDB	Zm00001d010016_P001	42	20893	4	4	3	3	60S ribosomal protein L11-1
68	1	Zea_mays_GDB	Zm00001d015259_P001	42	97439	1	1	1	1	26S proteasome non-ATPase regulatory subunit 2 homolog A
69	1	Zea_mays_GDB	Zm00001d036371_P001	41	72889	3	3	2	2	ATP-dependent zinc metalloprotease FTSH 5 chloroplastic
70	1	Zea_mays_GDB	Zm00001d010112_P001	40	65044	1	1	1	1	O-fucosyltransferase family protein
71	1	Zea_mays_GDB	Zm00001d009640_P001	40	38842	2	2	1	1	Malate dehydrogenase 2 mitochondrial
72	1	Zea_mays_GDB	Zm00001d000399_P001	39	61939	1	1	1	1	chloroplast protein synthesis2
73	1	Zea_mays_GDB	Zm00001d004804_P001	39	63430	1	1	1	1	Alkaline/neutral invertase CINV2
74	1	Zea_mays_GDB	Zm00001d025015_P001	38	67511	2	2	2	2	Glucose-6-phosphate 1-dehydrogenase
75	1	Zea_mays_GDB	Zm00001d032695_P001	38	35909	1	1	1	1	malate dehydrogenase4
76	1	Zea_mays_GDB	Zm00001d050496_P001	37	48523	2	2	1	1	Ribosomal protein L2 family
77	1	Zea_mays_GDB	Zm00001d014919_P001	37	44822	1	1	1	1	Glycerate dehydrogenase HPR peroxisomal
78	1	Zea_mays_GDB	Zm00001d014673_P001	37	47208	2	2	1	1	eucaryotic initiation factor4
79	1	Zea_mays_GDB	Zm00001d033480_P001	37	68642	1	1	1	1	5-methyltetrahydropteroyltriglutamate--homocysteine methyltransferase

80	1	Zea_mays_GDB	Zm00001d012878_P001	36	30112	2	2	2	2	ribosomal protein S4
81	1	Zea_mays_GDB	Zm00001d036765_P001	36	122535	1	1	1	1	Probable E3 ubiquitin ligase SUD1
82	1	Zea_mays_GDB	Zm00001d006388_P001	36	24503	1	1	1	1	Ribosomal protein L15
83	1	Zea_mays_GDB	Zm00001d030011_P001	36	83062	3	3	2	2	Eukaryotic translation initiation factor 3 subunit B
84	1	Zea_mays_GDB	Zm00001d008293_P001	35	88146	1	1	1	1	Probable splicing factor 3A subunit 1
85	1	Zea_mays_GDB	Zm00001d017772_P001	35	26468	1	1	1	1	Cation cation antiporter
86	1	Zea_mays_GDB	Zm00001d005367_P001	35	14996	1	1	1	1	Cell division control protein 48 homolog E
87	1	Zea_mays_GDB	Zm00001d003457_P001	35	21998	1	1	1	1	PLAT domain-containing protein 3
88	1	Zea_mays_GDB	Zm00001d025073_P001	34	43343	2	2	2	2	Protein argonaute 1
88	2	Zea_mays_GDB	Zm00001d002650_P001	26	120521	2	2	2	2	argonaute1c
89	1	Zea_mays_GDB	Zm00001d036959_P003	34	47375	2	2	2	2	elongation factor gamma1
90	1	Zea_mays_GDB	Zm00001d003923_P001	33	48860	1	1	1	1	Dihydrolipoyllysine-residue succinyltransferase component of 2-oxoglutar
91	1	Zea_mays_GDB	Zm00001d002711_P001	33	36021	2	2	1	1	Serine racemase
92	1	Zea_mays_GDB	Zm00001d007234_P001	33	27325	1	1	1	1	ascorbate peroxidase2
93	1	Zea_mays_GDB	Zm00001d004894_P001	32	19364	2	2	1	1	ribulose bisphosphate carboxylase small subunit2
94	1	Zea_mays_GDB	Zm00001d003048_P001	32	114963	1	1	1	1	Disease resistance gene analog PIC17
95	1	Zea_mays_GDB	ZemaCp052	32	26279	1	1	1	1	petB
96	1	Zea_mays_GDB	Zm00001d017711_P001	32	45121	1	1	1	1	Phosphoribulokinase
97	1	Zea_mays_GDB	Zm00001d006725_P001	32	49329	1	1	1	1	Eukaryotic peptide chain release factor subunit 1-2
98	1	Zea_mays_GDB	Zm00001d006899_P001	31	28755	2	2	1	1	40S ribosomal protein S6
99	1	Zea_mays_GDB	Zm00001d003427_P001	31	82960	1	1	1	1	DExH-box ATP-dependent RNA helicase DExH18 mitochondrial
100	1	Zea_mays_GDB	Zm00001d009013_P001	31	66651	2	2	1	1	bHLH-transcription factor 99
101	1	Zea_mays_GDB	Zm00001d042840_P001	31	41651	1	1	1	1	sedoheptulose bisphosphatase1
102	1	Zea_mays_GDB	Zm00001d015889_P001	31	87657	1	1	1	1	
103	1	Zea_mays_GDB	Zm00001d012641_P001	31	57772	2	2	1	1	cytokinin oxidase4b
104	1	Zea_mays_GDB	Zm00001d010431_P001	30	27466	3	3	2	2	Elongation factor 1-beta
105	1	Zea_mays_GDB	Zm00001d002325_P002	30	209006	2	2	1	1	
106	1	Zea_mays_GDB	Zm00001d003354_P001	30	160745	1	1	1	1	ABC transporter G family member 31
107	1	Zea_mays_GDB	Zm00001d031168_P001	30	15475	1	1	1	1	glycine-rich protein1
108	1	Zea_mays_GDB	Zm00001d046786_P001	30	28966	2	2	1	1	Photosystem I chlorophyll a/b-binding protein 3-1 chloroplastic
109	1	Zea_mays_GDB	Zm00001d022161_P001	30	88545	1	1	1	1	Putative paramyosin
110	1	Zea_mays_GDB	Zm00001d011992_P001	29	23936	2	2	1	1	60S ribosomal protein L13
111	1	Zea_mays_GDB	Zm00001d016248_P001	29	55332	2	2	2	2	Citrate synthase 3 peroxisomal
112	1	Zea_mays_GDB	Zm00001d028497_P001	29	69896	3	3	1	1	Putative dynamin-related protein 4A
113	1	Zea_mays_GDB	Zm00001d002261_P001	29	31372	2	2	2	2	Peroxisomal (S)-2-hydroxy-acid oxidase GLO1
114	1	Zea_mays_GDB	Zm00001d003947_P002	28	116872	2	2	2	2	2-oxoglutarate dehydrogenase E1 component
115	1	Zea_mays_GDB	Zm00001d004551_P001	28	21525	1	1	1	1	Peptidase S24/S26A/S26B/S26C family protein
116	1	Zea_mays_GDB	Zm00001d008970_P001	28	26696	1	1	1	1	Plastid division protein PDV1
117	1	Zea_mays_GDB	Zm00001d003110_P001	28	36925	1	1	1	1	Calmodulin-binding receptor-like cytoplasmic kinase 3
118	1	Zea_mays_GDB	Zm00001d016326_P001	27	70113	1	1	1	1	KH domain-containing protein
119	1	Zea_mays_GDB	Zm00001d020450_P001	27	13886	1	1	1	1	60S ribosomal protein L34-3
120	1	Zea_mays_GDB	Zm00001d003015_P001	27	77413	1	1	1	1	phenylalanine ammonia lyase6
121	1	Zea_mays_GDB	Zm00001d023379_P001	27	57758	1	1	1	1	pyruvate kinase2
122	1	Zea_mays_GDB	Zm00001d014792_P003	27	92874	1	1	1	1	shepherd-like1
123	1	Zea_mays_GDB	Zm00001d007069_P001	27	18702	2	2	1	1	NudC domain-containing protein 2
124	1	Zea_mays_GDB	Zm00001d006592_P001	27	13322	1	1	1	1	Chlorophyll a-b binding protein chloroplastic
125	1	Zea_mays_GDB	Zm00001d013910_P001	26	39699	1	1	1	1	DEAD-box ATP-dependent RNA helicase 8
126	1	Zea_mays_GDB	Zm00001d003657_P001	26	53928	1	1	1	1	GRAS-transcription factor 25
127	1	Zea_mays_GDB	Zm00001d042794_P001	26	49892	1	1	1	1	Transducin/WD40 repeat-like superfamily protein
128	1	Zea_mays_GDB	Zm00001d026421_P002	26	140682	1	1	1	1	DNA-directed RNA polymerases IV and V subunit 2
129	1	Zea_mays_GDB	Zm00001d008269_P001	25	60835	2	2	1	1	Rab escort protein 1
130	1	Zea_mays_GDB	Zm00001d008996_P001	25	61353	1	1	1	1	RNA polymerase I-associated factor PAF67
131	1	Zea_mays_GDB	Zm00001d023702_P001	25	12030	1	1	1	1	
132	1	Zea_mays_GDB	Zm00001d047497_P002	25	24284	1	1	1	1	Protein LAZ1 homolog 1
133	1	Zea_mays_GDB	Zm00001d028125_P001	24	42917	1	1	1	1	UPF0496 protein At3g28300
134	1	Zea_mays_GDB	Zm00001d003373_P001	24	38454	1	1	1	1	
135	1	Zea_mays_GDB	Zm00001d013245_P001	24	53531	1	1	1	1	UDP-glucose 6-dehydrogenase
136	1	Zea_mays_GDB	Zm00001d007960_P001	23	61109	2	2	2	2	T-complex protein 1 subunit epsilon
137	1	Zea_mays_GDB	Zm00001d037521_P001	23	47034	1	1	1	1	Acyl-coenzyme A oxidase 4 peroxisomal
138	1	Zea_mays_GDB	Zm00001d002292_P006	23	45506	1	1	1	1	NAD(P)-binding Rossmann-fold superfamily protein
139	1	Zea_mays_GDB	Zm00001d018034_P001	23	50406	1	1	1	1	geranylgeranyl hydrogenase1
140	1	Zea_mays_GDB	Zm00001d007937_P001	23	53934	1	1	1	1	alanine amino transferase8
141	1	Zea_mays_GDB	Zm00001d041593_P001	22	46948	1	1	1	1	ATP-citrate synthase
142	1	Zea_mays_GDB	Zm00001d005370_P002	22	51235	1	1	1	1	GPI mannosyltransferase 1
143	1	Zea_mays_GDB	Zm00001d006433_P001	22	23043	1	1	1	1	Ras-related protein RABH1b
144	1	Zea_mays_GDB	Zm00001d034066_P001	22	51170	1	1	1	1	UDP-glycosyltransferase 83A1
145	1	Zea_mays_GDB	Zm00001d002896_P001	22	50041	1	1	1	1	TUB-transcription factor 4
146	1	Zea_mays_GDB	Zm00001d049348_P003	22	19956	1	1	1	1	
147	1	Zea_mays_GDB	Zm00001d048592_P001	22	51235	1	1	1	1	RUBISCO activase3
148	1	Zea_mays_GDB	Zm00001d002543_P001	22	11402	1	1	1	1	H4C7 description
149	1	Zea_mays_GDB	Zm00001d012161_P001	22	34414	1	1	1	1	60S ribosomal protein L5-1 homolog b
150	1	Zea_mays_GDB	Zm00001d013192_P001	22	103248	1	1	1	1	Pyridoxal phosphate (PLP)-dependent transferases superfamily protein
151	1	Zea_mays_GDB	Zm00001d043192_P001	22	56313	1	1	1	1	
152	1	Zea_mays_GDB	Zm00001d034789_P001	22	104126	2	2	1	1	Replication protein-like
153	1	Zea_mays_GDB	Zm00001d053864_P001	21	26199	1	1	1	1	60S ribosomal protein L19-2
154	1	Zea_mays_GDB	Zm00001d012004_P003	21	30103	1	1	1	1	
155	1	Zea_mays_GDB	Zm00001d029173_P001	21	22416	1	1	1	1	Nascent polypeptide-associated complex alpha subunit-like protein
156	1	Zea_mays_GDB	Zm00001d012838_P001	21	45730	1	1	1	1	DCD (Development and Cell Death) domain protein
157	1	Zea_mays_GDB	Zm00001d038579_P001	21	49980	1	1	1	1	Phosphoglycerate kinase
158	1	Zea_mays_GDB	Zm00001d008975_P002	21	61229	1	1	1	1	Aspartate--tRNA ligase 2 cytoplasmic
159	1	Zea_mays_GDB	Zm00001d011474_P001	20	25500	1	1	1	1	Ran GTP binding protein
160	1	Zea_mays_GDB	Zm00001d029366_P001	20	18486	1	1	1	1	Transducin/WD40 repeat-like superfamily protein
161	1	Zea_mays_GDB	Zm00001d002989_P001	20	57909	1	1	1	1	cytokinin oxidase12
162	1	Zea_mays_GDB	Zm00001d007267_P001	20	23677	1	1	1	1	light harvesting chlorophyll a/b binding protein5
163	1	Zea_mays_GDB	Zm00001d003193_P004	20	10151	1	1	1	1	basic helix-loop-helix (bHLH) DNA-binding superfamily protein
164	1	Zea_mays_GDB	Zm00001d047044_P001	20	82014	1	1	1	1	RING/FYVE/PHD zinc finger superfamily protein
165	1	Zea_mays_GDB	Zm00001d002395_P001	20	28525	1	1	1	1	60S ribosomal protein L7-2
166	1	Zea_mays_GDB	Zm00001d027701_P001	20	64313	1	1	1	1	Appr-1-p processing enzyme family protein
167	1	Zea_mays_GDB	Zm00001d045312_P001	20	28928	1	1	1	1	
168	1	Zea_mays_GDB	Zm00001d023437_P001	20	112957	1	1	1	1	glycine decarboxylase1
169	1	Zea_mays_GDB	Zm00001d042115_P001	20	7808	1	1	1	1	
170	1	Zea_mays_GDB	Zm00001d006003_P001	20	9564	1	1	1	1	
171	1	Zea_mays_GDB	Zm00001d042427_P001	19	46045	1	1	1	1	Ribosomal protein S24/S35 mitochondrial
172	1	Zea_mays_GDB	Zm00001d007155_P001	19	84922	1	1	1	1	Pentatricopeptide repeat-containing protein
173	1	Zea_mays_GDB	Zm00001d016228_P001	19	55621	1	1	1	1	NHL domain-containing protein
174	1	Zea_mays_GDB	Zm00001d049620_P001	19	17141	1	1	1	1	
175	1	Zea_mays_GDB	Zm00001d012992_P002	19	15181	1	1	1	1	40S ribosomal protein S26-3
176	1	Zea_mays_GDB	Zm00001d005893_P001	19	85945	1	1	1	1	Protein WEAK CHLOROPLAST MOVEMENT UNDER BLUE LIGHT 1
177	1	Zea_mays_GDB	Zm00001d030223_P001	19	30214	1	1	1	1	ATP binding protein
178	1	Zea_mays_GDB	Zm00001d002810_P001	19	41167	1	1	1	1	SH3 domain-containing protein 2
179	1	Zea_mays_GDB	Zm00001d053620_P001	19	83751	2	2	1	1	Peptidyl-prolyl cis-trans isomerase CYP95
180	1	Zea_mays_GDB	Zm00001d037356_P001	19	109484	1	1	1	1	NF-X1-type zinc finger protein NFXL1
181	1	Zea_mays_GDB	Zm00001d037325_P001	19	13733	1	1	1	1	Phototropic-responsive NPH3 family protein
182	1	Zea_mays_GDB	Zm00001d032197_P001	18	26998	1	1	1	1	Chlorophyll a-b binding protein 4 chloroplastic
183	1	Zea_mays_GDB	Zm00001d051785_P001	18	16624	1	1	1	1	Protein CURVATURE THYLAKOID 1A chloroplastic
184	1	Zea_mays_GDB	Zm00001d018719_P001	18	64559	1	1	1	1	Electron transporter
185	1	Zea_mays_GDB	Zm00001d040704_P001	18	195821	1	1	1	1	
186	1	Zea_mays_GDB	Zm00001d035028_P001	18	27052	1	1	1	1	Putative glycolipid transfer protein (GLTP) family protein
187	1	Zea_mays_GDB	Zm00001d031690_P002	18	9088	1	1	1	1	
188	1	Zea_mays_GDB	Zm00001d021036_P001	17	42389	1	1	1	1	Calcium-dependent protein kinase 14
189	1	Zea_mays_GDB	Zm00001d051018_P002	17	28015	1	1	1	1	double B-box zinc finger protein4
190	1	Zea_mays_GDB	Zm00001d022046_P004	17	137445	1	1	1	1	SNF2 domain-containing protein / helicase domain-containing protein / H
191	1	Zea_mays_GDB	Zm00001d004674_P001	17	44032	1	1	1	1	Expressed protein; protein
192	1	Zea_mays_GDB	Zm00001d000417_P001	17	39100	1	1	1	1	psbA
193	1	Zea_mays_GDB	Zm00001d008727_P001	17	92797	1	1	1	1	phospholipase D2
194	1	Zea_mays_GDB	Zm00001d038465_P001	17	58196	1	1	1	1	Major facilitator superfamily protein
195	1	Zea_mays_GDB	Zm00001d024225_P001	16	256697	1	1	1	1	Histone-lysine N-methyltransferase ATXR3
196	1	Zea_mays_GDB	Zm00001d021272_P001	16	19683	1	1	1	1	
197	1	Zea_mays_GDB	Zm00001d002441_P001	16	317204	1	1	1	1	P-loop containing nucleoside triphosphate hydrolases superfamily protein
198	1	Zea_mays_GDB	Zm00001d000220_P001	16	61050	1	1	1	1	Pentatricopeptide repeat protein PPR868-14
199	1	Zea_mays_GDB	Zm00001d008531_P001	16	63748	1	1	1	1	
200	1	Zea_mays_GDB	ZemaCp022	15	82719	1	1	1	1	psaB
201	1	Zea_mays_GDB	Zm00001d019976_P001	15	61894	1	1	1	1	phosphoglucosamine mutase family protein
202	1	Zea_mays_GDB	Zm00001d014732_P001	15	16309	1	1	1	1	Peptidyl-prolyl cis-trans isomerase%2C microsomal
203	1	Zea_mays_GDB	Zm00001d052930_P003	15	42775	1	1	1	1	E3 ubiquitin-protein ligase RHF2A
204	1	Zea_mays_GDB	Zm00001d053746_P001	15	34862	1	1	1	1	Probable WRKY transcription factor 40

205	1	Zea_mays_GDB	Zm00001d012702_P002	15	109040	1	1	1	1	Phosphoenolpyruvate carboxylase 3
206	1	Zea_mays_GDB	Zm00001d037772_P001	15	109630	1	1	1	1	catalytics
207	1	Zea_mays_GDB	Zm00001d035937_P001	15	62041	1	1	1	1	Chaperonin 60 subunit beta 2 chloroplastic
208	1	Zea_mays_GDB	Zm00001d009146_P001	15	22614	1	1	1	1	S-adenosylmethionine synthase
209	1	Zea_mays_GDB	Zm00001d033805_P001	15	55640	1	1	1	1	Glutamate decarboxylase 1
210	1	Zea_mays_GDB	Zm00001d047449_P001	14	157172	1	1	1	1	Protein HUA2-LIKE 3
211	1	Zea_mays_GDB	Zm00001d015856_P001	14	45550	1	1	1	1	citrate synthase1
212	1	Zea_mays_GDB	Zm00001d051608_P014	14	16601	1	1	1	1	Fanconi-associated nuclease 1 homolog
213	1	Zea_mays_GDB	Zm00001d034160_P001	14	36010	1	1	1	1	G2-like-transcription factor 44
214	1	Zea_mays_GDB	Zm00001d011138_P001	14	63367	1	1	1	1	Protein IQ-DOMAIN 31
215	1	Zea_mays_GDB	Zm00001d004564_P001	14	62214	1	1	1	1	Aconitate hydratase 3 mitochondrial

Significance threshold $P<0.05$; Minimum number of significant unique sequences=1.

Repetition 2

Family	Member	Database	Accession	Score	Mass	Num. of matches	Num. of significant matches	Num. of sequences	Num. of significant sequences	Description
1	1	Zea_mays_GDB	Zm00001d042530_P003	5220	35103	131	131	23	23	IM30 protein homolog1 description
1	2	Zea_mays_GDB	Zm00001d012168_P001	4120	35042	104	104	25	25	Membrane-associated protein VIPP1 chloroplastic
1	3	Zea_mays_GDB	Zm00001d007452_P001	719	5996	12	12	2	2	Membrane-associated protein VIPP1 chloroplastic
2	1	Zea_mays_GDB	Zm00001d038955_P002	2975	69501	102	102	44	44	Dynamin-related protein 1A
2	2	Zea_mays_GDB	Zm00001d009504_P001	2756	73189	92	92	40	40	Dynamin protein 1A
2	3	Zea_mays_GDB	Zm00001d030005_P002	1301	45829	43	43	23	23	Dynamin-related protein 1E
2	4	Zea_mays_GDB	Zm00001d013426_P002	1282	68228	50	50	35	35	Dynamin-related protein 1C
2	5	Zea_mays_GDB	Zm00001d033751_P001	1263	68242	51	51	34	34	Dynamin-related protein 1C
2	6	Zea_mays_GDB	Zm00001d005306_P002	950	69758	27	27	18	18	Dynamin-related protein 1E
3	1	Zea_mays_GDB	Zm00001d046170_P001	2887	109843	97	97	53	53	phosphoenolpyruvate carboxylase1 description
3	2	Zea_mays_GDB	Zm00001d024980_P001	703	26811	17	17	8	8	Phosphoenolpyruvate carboxylase 3
3	3	Zea_mays_GDB	Zm00001d016166_P001	155	109987	7	7	7	7	phosphoenolpyruvate carboxylase2 description
3	4	Zea_mays_GDB	Zm00001d053453_P002	154	102279	6	6	6	6	phosphoenolpyruvate carboxylase3 description
3	5	Zea_mays_GDB	Zm00001d020057_P003	91	110593	5	5	4	4	phosphoenolpyruvate carboxylase4 description
3	6	Zea_mays_GDB	Zm00001d012702_P003	40	110661	3	3	3	3	Phosphoenolpyruvate carboxylase 3
3	7	Zea_mays_GDB	Zm00001d004885_P001	28	11468	1	1	1	1	aminopeptidases
4	1	Zea_mays_GDB	Zm00001d037279_P005	2690	99875	91	91	45	45	Dynamin-2A
4	2	Zea_mays_GDB	Zm00001d044757_P006	1969	99834	77	77	42	42	Dynamin-2A
4	3	Zea_mays_GDB	Zm00001d017979_P002	1121	100531	54	54	32	32	Dynamin-2A
5	1	Zea_mays_GDB	Zm00001d023218_P016	2437	194434	83	83	54	54	Putative clathrin heavy chain family protein
5	2	Zea_mays_GDB	Zm00001d005090_P018	2396	194872	78	78	52	52	Clathrin heavy chain 2
6	1	Zea_mays_GDB	Zm00001d031749_P002	2321	56378	67	67	26	26	Pyridoxal phosphate (PLP)-dependent transferases superfamily protein
7	1	Zea_mays_GDB	Zm00001d006403_P001	2314	54064	65	65	24	24	atpB description
7	2	Zea_mays_GDB	Zm00001d038929_P001	221	59181	9	9	7	7	ATP synthase2 description
7	3	Zea_mays_GDB	Zm00001d009488_P001	211	59085	9	9	7	7	csu849(atpb) description
8	1	Zea_mays_GDB	Zm00001d000279_P001	2093	54128	64	64	21	21	Ribulose bisphosphate carboxylase large chain
8	2	Zea_mays_GDB	Zm00001d006402_P004	1127	69751	37	37	15	15	Ribulose bisphosphate carboxylase large chain
8	3	Zea_mays_GDB	Zm00001d006402_P001	961	67712	28	28	6	6	Ribulose bisphosphate carboxylase large chain
8	4	Zea_mays_GDB	Zm00001d051485_P001	498	20524	16	16	2	2	Putative uncharacterized mitochondrial protein
8	5	Zea_mays_GDB	Zm00001d045698_P001	35	11396	1	1	1	1	Ribulose bisphosphate carboxylase large chain
9	1	Zea_mays_GDB	Zm00001d027511_P001	1689	56705	66	66	20	20	catalase2 description
9	2	Zea_mays_GDB	Zm00001d014848_P001	1276	57233	52	52	12	12	catalase1 description
9	3	Zea_mays_GDB	Zm00001d054044_P001	1083	56941	46	46	17	17	catalase3 description
10	1	Zea_mays_GDB	Zm00001d000270_P009	1619	55713	55	55	22	22	DNA-directed RNA polymerase subunit beta
10	2	Zea_mays_GDB	Zm00001d000270_P001	1276	266184	48	48	20	20	DNA-directed RNA polymerase subunit beta
11	1	Zea_mays_GDB	Zm00001d038065_P001	1599	36462	50	50	22	22	Annexin D4
12	1	Zea_mays_GDB	Zm00001d038163_P013	1536	87235	45	45	25	25	pyruvate, orthophosphate dikinase1 description
12	2	Zea_mays_GDB	Zm00001d010321_P015	138	44303	5	5	2	2	pyruvate, orthophosphate dikinase2 description
13	1	Zea_mays_GDB	Zm00001d013751_P001	1330	41741	48	48	20	20	UDP-arabinopyranose mutase 3
13	2	Zea_mays_GDB	Zm00001d033174_P001	1109	41717	45	45	19	19	Golgi associated protein homolog description
13	3	Zea_mays_GDB	Zm00001d022085_P001	701	41316	29	29	11	11	UDP-arabinopyranose mutase 3
13	4	Zea_mays_GDB	Zm00001d006820_P001	601	42246	22	22	8	8	UDP-arabinopyranose mutase 3
14	1	Zea_mays_GDB	Zm00001d006084_P007	1050	55009	34	34	18	18	Uridine kinase
14	2	Zea_mays_GDB	Zm00001d031602_P001	681	53892	23	23	18	18	Uridine kinase-like protein 2 chloroplastic
14	3	Zea_mays_GDB	Zm00001d054105_P003	670	54097	17	17	12	12	Uridine kinase
14	4	Zea_mays_GDB	Zm00001d014912_P008	561	54983	17	17	12	12	protein disulfide isomerase4 description
15	1	Zea_mays_GDB	Zm00001d008458_P001	1048	96830	38	38	14	14	**Cell-wall-associated receptor-like protein kinase**
16	1	Zea_mays_GDB	Zm00001d005485_P001	968	32059	28	28	10	10	prohibitin1 description
16	2	Zea_mays_GDB	Zm00001d034795_P001	760	31896	17	17	8	8	prohibitin4 description
17	1	Zea_mays_GDB	Zm00001d041550_P004	868	71517	26	26	19	19	heat shock protein70-4 description
17	2	Zea_mays_GDB	Zm00001d028630_P004	654	71493	19	19	12	12	Heat shock cognate 70 kDa protein 2
17	3	Zea_mays_GDB	Zm00001d010529_P001	631	71311	18	18	12	12	Probable mediator of RNA polymerase II transcription subunit 37c
17	4	Zea_mays_GDB	Zm00001d012420_P001	602	71180	19	19	13	13	heat shock protein1 description
17	5	Zea_mays_GDB	Zm00001d014993_P001	397	73211	11	11	6	6	Binding protein homolog1 description
18	1	Zea_mays_GDB	Zm00001d002543_P001	800	11402	25	25	11	11	H4C7 description
19	1	Zea_mays_GDB	Zm00001d025619_P001	724	30702	19	19	11	11	prohibitin2 description
20	1	Zea_mays_GDB	Zm00001d053726_P001	676	87747	22	22	17	17	Myosin heavy chain-related protein
21	1	Zea_mays_GDB	Zm00001d024037_P002	653	64308	28	28	18	18	beta-glucosidase2 description
21	2	Zea_mays_GDB	Zm00001d041777_P001	200	64866	11	11	9	9	Beta-glucosidase 17
21	3	Zea_mays_GDB	Zm00001d041776_P001	134	64576	6	6	6	6	dhurrinase2 description
21	4	Zea_mays_GDB	Zm00001d024000_P002	102	65343	6	6	5	5	beta-glucosidase3
21	5	Zea_mays_GDB	Zm00001d003045_P001	21	51924	1	1	1	1	Beta-glucosidase 47
22	1	Zea_mays_GDB	Zm00001d017092_P001	651	50790	16	16	12	12	Elongation factor Tu
22	2	Zea_mays_GDB	Zm00001d050969_P001	637	50972	16	16	12	12	Elongation factor Tu
22	3	Zea_mays_GDB	Zm00001d012846_P001	71	48658	5	5	2	2	Elongation factor Tu mitochondrial
22	4	Zea_mays_GDB	Zm00001d005228_P001	38	34267	3	3	1	1	Ribosomal protein L22p/L17e family protein
23	1	Zea_mays_GDB	Zm00001d003429_P001	626	43172	19	19	11	11	glyceraldehyde-3-phosphate dehydrogenase1 description
23	2	Zea_mays_GDB	Zm00001d027488_P001	476	47663	17	17	12	12	glyceraldehyde phosphate dehydrogenase B1 description
23	3	Zea_mays_GDB	Zm00001d025593_P001	148	44222	4	4	2	2	Glyceraldehyde-3-phosphate dehydrogenase GAPA1 chloroplastic
24	1	Zea_mays_GDB	Zm00001d015115_P001	567	43605	20	20	14	14	Ribose-phosphate pyrophosphokinase
25	1	Zea_mays_GDB	Zm00001d015274_P001	545	43661	18	18	11	11	Pyridoxamine 5-phosphate oxidase family protein
25	2	Zea_mays_GDB	Zm00001d031540_P001	441	39302	17	17	10	10	Pyridoxamine 5~-phosphate oxidase family protein
26	1	Zea_mays_GDB	Zm00001d052282_P006	530	178807	20	20	19	19	Nuclear-pore anchor
27	1	Zea_mays_GDB	Zm00001d038579_P001	524	49980	19	19	13	13	Phosphoglycerate kinase
27	2	Zea_mays_GDB	Zm00001d015376_P001	121	42470	6	6	5	5	Phosphoglycerate kinase
27	3	Zea_mays_GDB	Zm00001d010672_P001	86	18586	2	2	2	2	Metacaspase type II
27	4	Zea_mays_GDB	Zm00001d043194_P001	50	39038	2	2	2	2	Phosphoglycerate kinase
28	1	Zea_mays_GDB	Zm00001d044099_P002	520	50130	24	24	13	13	carbonic anhydrase1 description
28	2	Zea_mays_GDB	Zm00001d044096_P001	204	26766	12	12	7	7	carbonic anhydrase3 description
29	1	Zea_mays_GDB	Zm00001d049641_P001	501	36586	14	14	8	8	glyceraldehyde-3-phosphate dehydrogenase1 description
29	2	Zea_mays_GDB	Zm00001d035156_P001	445	35809	16	16	9	9	glyceraldehyde-3-phosphate dehydrogenase2 description
29	3	Zea_mays_GDB	Zm00001d015383_P001	78	44660	2	2	2	2	Glyceraldehyde-3-phosphate dehydrogenase%2C cytosolic
30	1	Zea_mays_GDB	Zm00001d023559_P001	484	41896	15	15	7	7	Fructose-bisphosphate aldolase
30	2	Zea_mays_GDB	Zm00001d053015_P001	81	42631	5	5	4	4	Fructose-bisphosphate aldolase
31	1	Zea_mays_GDB	Zm00001d021672_P001	481	45644	14	14	11	11	Pyruvate phosphate dikinase regulatory protein 2
32	1	Zea_mays_GDB	Zm00001d018632_P001	464	44219	15	15	9	9	Pyridoxal 5~-phosphate synthase-like subunit PDX1.2
32	2	Zea_mays_GDB	Zm00001d007936_P001	420	33693	13	13	8	8	Pyridoxal 5~-phosphate synthase-like subunit PDX1.2
33	1	Zea_mays_GDB	Zm00001d020580_P001	461	16410	14	14	6	6	histone2b1 description
33	2	Zea_mays_GDB	Zm00001d025913_P001	454	16134	14	14	6	6	Histone H2B
33	3	Zea_mays_GDB	Zm00001d047788_P002	71	7249	5	5	4	4	Histone H2B
34	1	Zea_mays_GDB	Zm00001d048593_P001	457	48079	12	12	8	8	RUBISCO activase2 description
35	1	Zea_mays_GDB	Zm00001d014734_P001	437	40647	26	26	15	15	GRMZM2G084942 description
35	2	Zea_mays_GDB	Zm00001d036439_P001	291	38937	15	15	9	9	Arogenate dehydrogenase isoform 2
35	3	Zea_mays_GDB	Zm00001d014737_P001	62	44454	4	4	2	2	Arogenate dehydrogenase 1 chloroplastic
36	1	Zea_mays_GDB	Zm00001d053765_P001	424	68689	15	15	14	14	vacuolar proton pump3 description
37	1	Zea_mays_GDB	Zm00001d021620_P001	415	40107	18	18	11	11	ATP synthase chloroplast subunit2 description
37	2	Zea_mays_GDB	Zm00001d044380_P001	150	18435	4	4	2	2	ATP synthase gamma chain 1 chloroplastic
37	3	Zea_mays_GDB	Zm00001d053572_P001	94	53503	4	4	2	2	RNA-binding (RRM/RBD/RNP motifs) family protein
37	4	Zea_mays_GDB	Zm00001d006475_P001	90	26291	4	4	2	2	ATP synthase gamma chain 1 chloroplastic
38	1	Zea_mays_GDB	Zm00001d046742_P001	401	39771	9	9	5	5	Ribose-phosphate pyrophosphokinase
39	1	Zea_mays_GDB	Zm00001d000316_P001	382	66519	10	10	8	8	NADP malic enzyme3 description
39	2	Zea_mays_GDB	Zm00001d037961_P001	90	71107	3	3	2	2	NADP-dependent malic enzyme 3
40	1	Zea_mays_GDB	Zm00001d048736_P001	370	49157	11	11	7	7	S-alkyl-thiohydroximate lyase SUR1
41	1	Zea_mays_GDB	Zm00001d010255_P001	276	51286	12	12	8	8	Guanosine nucleotide diphosphate dissociation inhibitor 2
41	2	Zea_mays_GDB	Zm00001d029596_P001	102	50634	2	2	1	1	Guanosine nucleotide diphosphate dissociation inhibitor
41	3	Zea_mays_GDB	Zm00001d010251_P001	37	50346	3	3	3	3	Guanosine nucleotide diphosphate dissociation inhibitor 2
42	1	Zea_mays_GDB	Zm00001d006871_P001	354	33179	9	9	6	6	40S ribosomal protein Sa-1
42	2	Zea_mays_GDB	Zm00001d048346_P001	351	33798	8	8	5	5	acidic ribosomal protein P40 description
42	3	Zea_mays_GDB	Zm00001d048253_P004	261	17109	7	7	5	5	40S ribosomal protein Sa-1

42	4	Zea_mays_GDB	Zm00001d027919_P001	257	33875	6	6	4	4	40S ribosomal protein Sa-1
43	1	Zea_mays_GDB	Zm00001d033132_P001	351	27948	16	16	7	7	photosystem II light harvesting complex gene 2.1
43	2	Zea_mays_GDB	Zm00001d039040_P001	348	28180	12	12	6	6	light harvesting complex mesophyll7 description
43	3	Zea_mays_GDB	Zm00001d011285_P001	304	28199	7	7	4	4	light harvesting chlorophyll a/b binding protein1 description
43	4	Zea_mays_GDB	Zm00001d021435_P001	280	27912	8	8	5	5	light harvesting chlorophyll a/b binding protein2 description
43	5	Zea_mays_GDB	Zm00001d009589_P001	245	28093	9	9	5	5	light harvesting chlorophyll a/b binding protein3 description
43	6	Zea_mays_GDB	Zm00001d044399_P001	240	28098	7	7	4	4	photosystem II light harvesting complex gene B1B2
43	7	Zea_mays_GDB	Zm00001d044396_P001	220	28128	6	6	3	3	Chlorophyll a-b binding protein 48%2C chloroplastic
44	1	Zea_mays_GDB	Zm00001d001814_P001	350	32423	14	14	6	6	Clathrin light chain 2
44	2	Zea_mays_GDB	Zm00001d026630_P001	337	32884	13	13	6	6	Clathrin light chain 2
45	1	Zea_mays_GDB	Zm00001d018810_P001	350	45430	13	13	9	9	glycolate oxidase1 description
45	2	Zea_mays_GDB	Zm00001d002261_P001	130	31372	5	5	5	5	Peroxisomal (S)-2-hydroxy-acid oxidase GLO1
46	1	Zea_mays_GDB	Zm00001d037513_P001	332	22101	14	14	4	4	germin-like protein1 description
47	1	Zea_mays_GDB	Zm00001d007824_P001	332	44498	12	12	7	7	60S ribosomal protein L4-1
47	2	Zea_mays_GDB	Zm00001d018957_P001	239	44379	9	9	6	6	60S ribosomal protein L4-1
47	3	Zea_mays_GDB	Zm00001d013076_P001	270	44483	9	9	5	5	60S ribosomal protein L4-1
47	4	Zea_mays_GDB	Zm00001d034440_P001	211	44594	7	7	4	4	60S ribosomal protein L4-1
48	1	Zea_mays_GDB	Zm00001d018779_P001	330	32073	7	7	6	6	photosystem II oxygen evolving polypeptide2 description
48	2	Zea_mays_GDB	Zm00001d007857_P001	123	27411	4	4	3	3	photosystem II oxygen evolving polypeptide1 description
49	1	Zea_mays_GDB	Zm00001d001812_P001	326	51001	12	12	10	10	Rubisco Assembly Factor 1 description
50	1	Zea_mays_GDB	Zm00001d025273_P008	316	102122	11	11	11	11	Double Clp-N motif-containing P-loop nucleoside triphosphate hydrolas
50	2	Zea_mays_GDB	Zm00001d015520_P001	76	109769	2	2	2	2	Chaperone protein ClpB4 mitochondrial
50	3	Zea_mays_GDB	Zm00001d038806_P001	42	101284	1	1	1	1	heat-shock protein 101 description
51	1	Zea_mays_GDB	Zm00001d005238_P001	308	51340	8	8	7	7	Eukaryotic translation initiation factor 3 subunit E
52	1	Zea_mays_GDB	Zm00001d030011_P001	302	83062	17	17	13	13	Eukaryotic translation initiation factor 3 subunit B
53	1	Zea_mays_GDB	Zm00001d053630_P001	301	25155	7	7	5	5	ribosomal protein S8 homolog description
54	1	Zea_mays_GDB	Zm00001d042697_P001	291	28434	6	6	3	3	photosystem II subunit PsbS1 description
55	1	Zea_mays_GDB	Zm00001d006540_P001	286	23037	14	14	9	9	Oxygen-evolving enhancer protein 3-1
55	2	Zea_mays_GDB	Zm00001d021703_P001	286	23119	14	14	9	9	oxygen evolving complex2 description
56	1	Zea_mays_GDB	Zm00001d013507_P001	282	73357	10	10	8	8	Heat shock 70 kDa protein 6 chloroplastic
56	2	Zea_mays_GDB	Zm00001d023802_P001	277	74853	9	9	7	7	Heat shock 70 kDa protein 6 chloroplastic
57	1	Zea_mays_GDB	Zm00001d050375_P001	282	28960	10	10	7	7	14-3-3-like protein
57	2	Zea_mays_GDB	Zm00001d003401_P001	278	29758	8	8	6	6	general regulatory factor1 description
57	3	Zea_mays_GDB	Zm00001d007446_P002	141	29411	4	4	4	4	14-3-3-like protein
57	4	Zea_mays_GDB	Zm00001d052796_P003	106	29808	3	3	3	3	14-3-3-like protein A
57	5	Zea_mays_GDB	Zm00001d052698_P001	68	28687	2	2	2	2	14-3-3-like protein GF14 nu
58	1	Zea_mays_GDB	Zm00001d036961_P004	281	54142	10	10	10	10	Putative ATPase%2C V1 complex%2C subunit B protein isoform 1%3B Putativ
59	1	Zea_mays_GDB	Zm00001d052595_P001	271	19424	13	13	8	8	ribulose bisphosphate carboxylase small subunit1 description
59	2	Zea_mays_GDB	Zm00001d004894_P001	250	19364	11	11	7	7	ribulose bisphosphate carboxylase small subunit2 description
60	1	Zea_mays_GDB	Zm00001d042050_P001	269	44032	12	12	9	9	Protein RETICULATA-RELATED 4 chloroplastic
61	1	Zea_mays_GDB	Zm00001d038923_P001	268	36670	8	8	5	5	receptor for activated C kinase1 description
62	1	Zea_mays_GDB	Zm00001d007267_P002	263	30266	7	7	3	3	light harvesting chlorophyll a/b binding protein5 description
63	1	Zea_mays_GDB	Zm00001d012276_P001	252	15332	8	8	4	4	Histone H3.2
63	2	Zea_mays_GDB	Zm00001d016908_P001	187	15454	8	8	4	4	uaz248b(his3) description
64	1	Zea_mays_GDB	Zm00001d016702_P006	246	58329	9	9	6	6	Iron-sulphur cluster biosynthesis family protein
65	1	Zea_mays_GDB	Zm00001d042049_P002	243	41180	6	6	5	5	Ferredoxin
65	2	Zea_mays_GDB	Zm00001d045575_P001	226	39640	4	4	3	3	Ferredoxin--NADP reductase leaf isozyme 1 chloroplastic
66	1	Zea_mays_GDB	Zm00001d036535_P001	242	34783	9	9	6	6	oxygen evolving complex, 33kDa subunit description
66	2	Zea_mays_GDB	Zm00001d014564_P001	188	39337	8	8	5	5	oxygen-evolving complex 33 kda protein b description
67	1	Zea_mays_GDB	Zm00001d013410_P001	242	41998	12	12	7	7	Actin-1
67	2	Zea_mays_GDB	Zm00001d004855_P002	216	66135	11	11	6	6	Actin-7
68	1	Zea_mays_GDB	Zm00001d017707_P001	236	71705	10	10	8	8	P-loop containing nucleoside triphosphate hydrolases superfamily protei
68	2	Zea_mays_GDB	Zm00001d017783_P001	64	69525	4	4	4	4	AAA-type ATPase family protein
69	1	Zea_mays_GDB	Zm00001d033527_P003	228	44296	7	7	6	6	magnesium transporter12 description
70	1	Zea_mays_GDB	Zm00001d003680_P001	227	44892	8	8	6	6	60S ribosomal protein L3-1
70	2	Zea_mays_GDB	Zm00001d002462_P009	37	28790	1	1	1	1	Peroxisome biogenesis protein 6
71	1	Zea_mays_GDB	Zm00001d033594_P001	225	39978	6	6	4	4	Myelin-associated oligodendrocyte basic protein isoform 1
71	2	Zea_mays_GDB	Zm00001d013506_P001	192	40923	5	5	3	3	Myelin-associated oligodendrocyte basic protein isoform 1
72	1	Zea_mays_GDB	Zm00001d052948_P001	214	59382	5	5	5	5	Putative TCP-1/cpn60 chaperonin family protein
72	2	Zea_mays_GDB	Zm00001d007508_P003	186	61511	5	5	5	5	T-complex protein 1 subunit theta
73	1	Zea_mays_GDB	Zm00001d035094_P001	213	17203	7	7	4	4	40S ribosomal protein S13-1
74	1	Zea_mays_GDB	Zm00001d031899_P001	211	44631	5	5	3	3	malate dehydrogenase6 description
75	1	Zea_mays_GDB	Zm00001d018873_P001	210	36351	8	8	7	7	Eukaryotic translation initiation factor 3 subunit 2
75	2	Zea_mays_GDB	Zm00001d007900_P002	188	36379	7	7	6	6	Eukaryotic translation initiation factor 3 subunit I
76	1	Zea_mays_GDB	Zm00001d053857_P001	209	49547	6	6	6	6	T-complex protein 1 subunit eta
77	1	Zea_mays_GDB	Zm00001d046786_P001	209	28966	8	8	5	5	Photosystem I chlorophyll a/b-binding protein 3-1 chloroplastic
78	1	Zea_mays_GDB	Zm00001d010821_P002	207	103720	11	11	10	10	starch synthase4 description
78	2	Zea_mays_GDB	Zm00001d051976_P001	42	56638	2	2	2	2	starch synthase5 description
79	1	Zea_mays_GDB	Zm00001d018349_P001	207	59457	5	5	4	4	T-complex protein 1 subunit alpha
80	1	Zea_mays_GDB	Zm00001d024511_P001	205	25713	6	6	5	5	ribosomal protein S3
81	1	Zea_mays_GDB	Zm00001d006008_P001	199	80407	5	5	5	5	Heat shock protein 90-2
82	1	Zea_mays_GDB	Zm00001d024281_P001	199	56449	6	6	6	6	polyamine oxidase1 description
83	1	Zea_mays_GDB	Zm00001d008757_P001	196	46786	5	5	5	5	Proteasome component (PCI) domain protein
84	1	Zea_mays_GDB	Zm00001d036630_P001	194	49284	4	4	3	3	Filamentation temperature-sensitive H 2B%3B Filamentation temperature-se
85	1	Zea_mays_GDB	Zm00001d040257_P001	194	57701	5	5	4	4	T-complex protein 1 subunit beta
86	1	Zea_mays_GDB	Zm00001d000270_P010	192	10121	5	5	5	5	DNA-directed RNA polymerase subunit beta
87	1	Zea_mays_GDB	Zm00001d024540_P001	192	43307	7	7	6	6	stomatin1 description
88	1	Zea_mays_GDB	Zm00001d037873_P001	190	49478	8	8	6	6	elongation factor alpha3 description
89	1	Zea_mays_GDB	Zm00001d006899_P001	188	28755	5	5	5	5	40S ribosomal protein S6
90	1	Zea_mays_GDB	Zm00001d035937_P001	182	62041	5	5	5	5	Chaperonin 60 subunit beta 2 chloroplastic
91	1	Zea_mays_GDB	Zm00001d028471_P001	179	73913	7	7	7	7	phosphoenolpyruvate carboxykinase1 description
92	1	Zea_mays_GDB	Zm00001d009848_P001	178	21187	5	5	4	4	60S ribosomal protein L18-3
92	2	Zea_mays_GDB	Zm00001d022462_P001	66	21144	2	2	2	2	60S ribosomal protein L18-3
93	1	Zea_mays_GDB	Zm00001d028620_P001	175	70926	4	4	4	4	Putative clathrin assembly protein
94	1	Zea_mays_GDB	Zm00001d002235_P009	174	62586	5	5	5	5	
95	1	Zea_mays_GDB	Zm00001d036565_P001	172	16436	6	6	5	5	40S ribosomal protein S14-3
96	1	Zea_mays_GDB	Zm00001d017118_P001	172	29628	8	8	5	5	csu173 description
97	1	Zea_mays_GDB	Zm00001d052242_P001	171	26704	3	3	2	2	ATP synthase subunit delta chloroplastic
98	1	Zea_mays_GDB	Zm00001d021494_P001	169	116594	8	8	7	7	C2 calcium/lipid-binding plant phosphoribosyltransferase family protei
98	2	Zea_mays_GDB	Zm00001d006371_P001	149	116116	6	6	6	6	C2 calcium/lipid-binding plant phosphoribosyltransferase family protei
99	1	Zea_mays_GDB	Zm00001d034771_P001	166	94813	4	4	4	4	Elongation factor 2
100	1	Zea_mays_GDB	Zm00001d036046_P001	163	34712	4	4	3	3	Magnesium protoporphyrin IX methyltransferase chloroplastic
101	1	Zea_mays_GDB	Zm00001d002166_P022	163	72491	4	4	4	4	Eukaryotic translation initiation factor 3 subunit C
102	1	Zea_mays_GDB	Zm00001d037929_P001	162	22297	4	4	3	3	40S ribosomal protein S5-2
103	1	Zea_mays_GDB	Zm00001d023583_P001	161	83640	4	4	3	3	Dynamin-like protein ARC5
104	1	Zea_mays_GDB	Zm00001d032187_P001	160	41373	4	4	3	3	Malate dehydrogenase
105	1	Zea_mays_GDB	Zm00001d019473_P001	160	28256	4	4	4	4	60S ribosomal protein L8-3
106	1	Zea_mays_GDB	Zm00001d012161_P001	158	34414	4	4	3	3	60S ribosomal protein L5-1 homolog b
107	1	Zea_mays_GDB	Zm00001d041760_P001	158	22475	7	7	6	6	40S ribosomal protein S9-2
107	2	Zea_mays_GDB	Zm00001d006923_P002	89	22560	5	5	5	5	40S ribosomal protein S9-2
108	1	Zea_mays_GDB	Zm00001d039518_P001	157	112004	7	7	7	7	eukaryotic initiation factor3 description
109	1	Zea_mays_GDB	Zm00001d007192_P001	156	60092	6	6	6	6	T-complex protein 1 subunit zeta
111	1	Zea_mays_GDB	Zm00001d047789_P001	153	22758	5	5	4	4	ATP synthase B chain
112	1	Zea_mays_GDB	Zm00001d035201_P001	152	34581	4	4	3	3	60S acidic ribosomal protein P0 description
113	1	Zea_mays_GDB	Zm00001d015886_P001	150	46865	4	4	4	4	26S protease regulatory subunit 6B homolog
113	2	Zea_mays_GDB	Zm00001d018409_P002	66	47875	4	4	4	4	26S protease regulatory subunit 6A homolog A
113	3	Zea_mays_GDB	Zm00001d015788_P001	40	44771	2	2	2	2	proteasome component4 description
114	1	Zea_mays_GDB	Zm00001d047581_P001	148	45966	7	7	6	6	30S ribosomal protein S1
115	1	Zea_mays_GDB	Zm00001d006520_P001	146	46170	6	6	5	5	PDK regulatory protein1 description
116	1	Zea_mays_GDB	Zm00001d028117_P001	145	29951	5	5	5	5	40S ribosomal protein S3a-2
117	1	Zea_mays_GDB	Zm00001d005023_P001	144	16853	4	4	2	2	40S ribosomal protein S16
118	1	Zea_mays_GDB	Zm00001d043972_P001	143	18961	3	3	1	1	50S ribosomal protein L12-1
119	1	Zea_mays_GDB	Zm00001d019147_P001	142	30566	5	5	4	4	40S ribosomal protein S2
119	2	Zea_mays_GDB	Zm00001d013034_P001	79	30027	3	3	3	3	40S ribosomal protein S2-1
120	1	Zea_mays_GDB	Zm00001d014673_P001	139	47208	5	5	5	5	eucaryotic initiation factor4 description
121	1	Zea_mays_GDB	Zm00001d049018_P001	139	45457	10	10	8	8	Trypsin family protein with PDZ domain
122	1	Zea_mays_GDB	Zm00001d019312_P001	135	19161	1	1	1	1	beta glucosidase aggregating factor1 description
123	1	Zea_mays_GDB	Zm00001d018532_P001	134	120555	3	3	2	2	
124	1	Zea_mays_GDB	Zm00001d002395_P001	133	28525	10	10	8	8	60S ribosomal protein L7-2
124	2	Zea_mays_GDB	Zm00001d026254_P001	133	28715	10	10	8	8	60S ribosomal protein L7-2
125	1	Zea_mays_GDB	Zm00001d016736_P001	131	28318	6	6	5	5	2-Cys peroxiredoxin BAS1 chloroplastic
126	1	Zea_mays_GDB	Zm00001d009877_P001	130	51878	4	4	4	4	Protein plastid transcriptionally active 16 chloroplastic
127	1	Zea_mays_GDB	Zm00001d023707_P002	129	19085	2	2	2	2	thioredoxin M1 description
128	1	Zea_mays_GDB	Zm00001d038084_P001	127	37249	3	3	3	3	50S ribosomal protein L1 chloroplastic
129	1	Zea_mays_GDB	Zm00001d050920_P002	125	24227	5	5	4	4	60S ribosomal protein L6
130	1	Zea_mays_GDB	Zm00001d011100_P001	124	31398	3	3	3	3	Eukaryotic translation initiation factor 3 subunit F

130	2	Zea_mays_GDB	Zm00001d035752_P001	107	31501	3	3	3	3	STIP1 and U box-containing protein 1%3B STIP1 homology and U box-containi
131	1	Zea_mays_GDB	Zm00001d004784_P001	119	40922	3	3	3	3	Protein TPLATE
132	1	Zea_mays_GDB	Zm00001d043606_P001	117	15648	4	4	2	2	40S ribosomal protein S24
133	1	Zea_mays_GDB	Zm00001d004344_P001	116	42290	2	2	2	2	Natterin-4
135	1	Zea_mays_GDB	Zm00001d013252_P001	114	23842	5	5	3	3	60S ribosomal protein L13a-1
135	2	Zea_mays_GDB	Zm00001d034059_P001	108	27402	4	4	2	2	ribosomal protein L13A homolog description
136	1	Zea_mays_GDB	Zm00001d018487_P001	113	48499	7	7	5	5	3-ketoacyl-CoA thiolase 2 peroxisomal
137	1	Zea_mays_GDB	ZemaCp047	110	24913	4	4	3	3	clpP description
138	1	Zea_mays_GDB	Zm00001d025185_P001	109	39346	3	3	3	3	Eukaryotic translation initiation factor 3 subunit H
139	1	Zea_mays_GDB	Zm00001d000399_P001	108	61939	2	2	2	2	chloroplast protein synthesis2 description
139	2	Zea_mays_GDB	Zm00001d034919_P001	40	61419	2	2	2	2	RuBisCO large subunit-binding protein subunit alpha
140	1	Zea_mays_GDB	Zm00001d039167_P001	107	42955	4	4	4	4	ATP-dependent Clp protease proteolytic subunit
141	1	Zea_mays_GDB	Zm00001d045451_P001	106	79444	3	3	3	3	transketolase 1 description
142	1	Zea_mays_GDB	Zm00001d021763_P001	106	31422	4	4	4	4	photosystem II subunit29 description
143	1	Zea_mays_GDB	Zm00001d036371_P001	105	72889	3	3	3	3	ATP-dependent zinc metalloprotease FTSH 5 chloroplastic
144	1	Zea_mays_GDB	Zm00001d045430_P001	104	32183	1	1	1	1	ATP-dependent Clp protease proteolytic subunit
145	1	Zea_mays_GDB	Zm00001d037103_P001	103	23781	2	2	2	2	Peroxiredoxin Q chloroplastic
146	1	Zea_mays_GDB	Zm00001d019518_P001	103	14401	3	3	3	3	Photosystem I reaction center subunit IV A
147	1	Zea_mays_GDB	ZemaCp058	103	15686	3	3	2	2	rps8 description
148	1	Zea_mays_GDB	Zm00001d017857_P001	102	42593	4	4	4	4	adenine nucleotide translocator1 description
149	1	Zea_mays_GDB	Zm00001d046553_P002	102	68480	2	2	2	2	Fructose-bisphosphate aldolase 7 cytosolic
150	1	Zea_mays_GDB	ZemaCp076	101	17648	2	2	2	2	rps7-A description
151	1	Zea_mays_GDB	Zm00001d023379_P001	101	57758	1	1	1	1	pyruvate kinase2
152	1	Zea_mays_GDB	Zm00001d053675_P001	101	102447	4	4	4	4	lipoxygenase10 description
153	1	Zea_mays_GDB	ZemaCp052	100	26279	3	3	2	2	petB
154	1	Zea_mays_GDB	ZemaCp021	100	12229	4	4	3	3	rps14 description
155	1	Zea_mays_GDB	Zm00001d035901_P001	100	21505	4	4	4	4	60S ribosomal protein L9-1
155	2	Zea_mays_GDB	Zm00001d054084_P001	44	21418	2	2	2	2	60S ribosomal protein L9-1
156	1	Zea_mays_GDB	Zm00001d010566_P001	99	15781	2	2	2	2	40S ribosomal protein S23-2
157	1	Zea_mays_GDB	ZemaCp026	99	23505	3	3	2	2	RPS4 description
158	1	Zea_mays_GDB	Zm00001d007937_P001	98	53934	5	5	5	5	alanine amino transferase8 description
160	1	Zea_mays_GDB	Zm00001d039305_P001	98	61538	6	6	6	6	RNA polymerase I-associated factor PAF67
161	1	Zea_mays_GDB	Zm00001d007960_P001	98	61109	3	3	3	3	T-complex protein 1 subunit epsilon
162	1	Zea_mays_GDB	Zm00001d031956_P001	97	24705	3	3	3	3	Ribosomal protein
162	2	Zea_mays_GDB	Zm00001d042772_P001	25	29352	2	2	2	2	Ribosomal protein
163	1	Zea_mays_GDB	Zm00001d034422_P001	97	14820	5	5	4	4	40S ribosomal protein S18
163	2	Zea_mays_GDB	Zm00001d013086_P001	83	17741	6	6	5	5	40S ribosomal protein S18
164	1	Zea_mays_GDB	Zm00001d013777_P001	97	74573	3	3	3	3	MYB-related-transcription factor 40
165	1	Zea_mays_GDB	Zm00001d012878_P001	96	30112	6	6	5	5	ribosomal protein S4 description
165	2	Zea_mays_GDB	Zm00001d035797_P001	52	63865	2	2	2	2	DEAD-box ATP-dependent RNA helicase 7
165	3	Zea_mays_GDB	Zm00001d006309_P002	51	39095	2	2	2	2	UDP-glucose pyrophosphorylase1 description
166	1	Zea_mays_GDB	Zm00001d016200_P001	94	21055	4	4	4	4	
166	2	Zea_mays_GDB	Zm00001d010016_P001	89	20893	4	4	4	4	60S ribosomal protein L11-1
167	1	Zea_mays_GDB	Zm00001d029121_P005	93	37083	2	2	2	2	ATP-dependent Clp protease proteolytic subunit
168	1	Zea_mays_GDB	Zm00001d013039_P001	91	25320	5	5	4	4	photosystem I subunit d1
169	1	Zea_mays_GDB	Zm00001d000417_P001	91	39100	2	2	2	2	psbA description
170	1	Zea_mays_GDB	Zm00001d012598_P001	91	12015	2	2	2	2	40S ribosomal protein S25-2
171	1	Zea_mays_GDB	Zm00001d031349_P001	91	44363	6	6	4	4	Serine--glyoxylate aminotransferase
172	1	Zea_mays_GDB	Zm00001d015259_P001	90	97439	5	5	4	4	26S proteasome non-ATPase regulatory subunit 2 homolog A
173	1	Zea_mays_GDB	Zm00001d023910_P001	90	68928	4	4	4	4	Adaptor complexe medium subunit family protein
174	1	Zea_mays_GDB	Zm00001d038745_P001	89	57834	3	3	3	3	SNF1-related protein kinase catalytic subunit alpha KIN10
175	1	Zea_mays_GDB	Zm00001d028998_P001	88	41036	6	6	5	5	hydroxyproline-rich glycoprotein family protein
176	1	Zea_mays_GDB	Zm00001d026599_P001	87	26570	2	2	2	2	light harvesting chlorophyll a/b binding protein6 description
177	1	Zea_mays_GDB	Zm00001d029502_P001	87	72271	3	3	3	3	Glucose-6-phosphate 1-dehydrogenase
177	2	Zea_mays_GDB	Zm00001d025015_P001	73	67511	5	5	4	4	Glucose-6-phosphate 1-dehydrogenase
178	1	Zea_mays_GDB	Zm00001d006388_P001	85	24503	2	2	2	2	Ribosomal protein L15
179	1	Zea_mays_GDB	Zm00001d028183_P001	85	58500	5	5	5	5	T-complex protein 1 subunit gamma
180	1	Zea_mays_GDB	Zm00001d034808_P001	84	23902	4	4	3	3	30S ribosomal protein S6 alpha chloroplastic
181	1	Zea_mays_GDB	Zm00001d003127_P001	84	16930	2	2	1	1	60S ribosomal protein L23a-1
182	1	Zea_mays_GDB	Zm00001d011992_P001	84	23936	4	4	3	3	60S ribosomal protein L13
183	1	Zea_mays_GDB	Zm00001d007158_P003	83	94001	2	2	2	2	Probable acyl-CoA dehydrogenase IBR3
184	1	Zea_mays_GDB	Zm00001d032197_P001	83	26998	3	3	2	2	Chlorophyll a-b binding protein 4 chloroplastic
185	1	Zea_mays_GDB	Zm00001d045000_P001	82	16433	3	3	3	3	40S ribosomal protein S17-4
186	2	Zea_mays_GDB	Zm00001d044246_P002	56	16434	5	5	4	4	Histone H2A
186	3	Zea_mays_GDB	Zm00001d035619_P001	49	16270	5	5	4	4	Histone H2A
186	4	Zea_mays_GDB	Zm00001d006547_P001	44	14036	3	3	2	2	Histone H2A
186	5	Zea_mays_GDB	Zm00001d012837_P001	40	15714	2	2	2	2	Histone H2A
186	6	Zea_mays_GDB	Zm00001d013300_P001	38	14464	3	3	2	2	Histone H2A
186	7	Zea_mays_GDB	Zm00001d007553_P001	35	20508	1	1	1	1	Probable histone H2A variant 3
187	1	Zea_mays_GDB	Zm00001d043955_P001	81	65074	3	3	3	3	Eukaryotic translation initiation factor 3 subunit D
187	2	Zea_mays_GDB	Zm00001d039038_P001	71	65308	3	3	3	3	Eukaryotic translation initiation factor 3 subunit D
188	1	Zea_mays_GDB	Zm00001d028562_P001	79	44972	6	6	5	5	Fructose-1%2C6-bisphosphatase
189	1	Zea_mays_GDB	Zm00001d031128_P001	79	82279	3	3	3	3	5-methyltetrahydropteroyltriglutamate--homocysteine methyltransferase 1
189	2	Zea_mays_GDB	Zm00001d013644_P001	68	88785	3	3	3	3	5-methyltetrahydropteroyltriglutamate--homocysteine methyltransferase 1
190	1	Zea_mays_GDB	ZemaCp059	78	13542	3	3	3	3	rpl14 description
191	1	Zea_mays_GDB	Zm00001d005648_P002	78	31488	1	1	1	1	hypersensitive induced response2 description
192	1	Zea_mays_GDB	Zm00001d048252_P001	71	25830	2	2	2	2	Eukaryotic translation initiation factor 3 subunit K
193	1	Zea_mays_GDB	Zm00001d006064_P001	76	15929	1	1	1	1	60S ribosomal protein L32-1
194	1	Zea_mays_GDB	Zm00001d006287_P001	75	15395	5	5	4	4	60S ribosomal protein L14-1
194	2	Zea_mays_GDB	Zm00001d021445_P001	62	19407	4	4	4	4	60S ribosomal protein L14-1
195	1	Zea_mays_GDB	Zm00001d035095_P001	74	16545	2	2	2	2	Calmodulin
196	1	Zea_mays_GDB	Zm00001d023431_P001	74	61237	1	1	1	1	Signal recognition particle 54 kDa protein chloroplastic
197	1	Zea_mays_GDB	Zm00001d023536_P001	73	46131	2	2	2	2	oil yellow1 description
198	1	Zea_mays_GDB	Zm00001d003083_P001	73	54928	2	2	1	1	Isocitrate dehydrogenase
199	1	Zea_mays_GDB	Zm00001d006651_P001	73	50221	3	3	2	2	alpha tubulin5 description
199	2	Zea_mays_GDB	Zm00001d013367_P001	64	50370	4	4	2	2	alpha tubulin4 description
200	1	Zea_mays_GDB	Zm00001d002034_P001	73	22932	2	2	1	1	6%2C7-dimethyl-8-ribityllumazine synthase
201	1	Zea_mays_GDB	Zm00001d010631_P001	72	18455	3	3	3	3	60S ribosomal protein L24%3B 60S ribosomal protein L24 isoform 1%3B 60
202	1	Zea_mays_GDB	Zm00001d018034_P001	71	50406	1	1	1	1	geranylgeranyl hydrogenase1 description
203	1	Zea_mays_GDB	Zm00001d011504_P001	71	26727	3	3	3	3	V-type proton ATPase subunit E3
204	1	Zea_mays_GDB	Zm00001d042840_P001	70	41651	1	1	1	1	sedoheptulose bisphosphatase1 description
205	1	Zea_mays_GDB	Zm00001d012027_P001	70	90524	3	3	3	3	Dynamin-related protein 3A
206	1	Zea_mays_GDB	Zm00001d043335_P001	70	110463	2	2	2	2	Branched-chain-amino-acid aminotransferase 5 chloroplastic
207	1	Zea_mays_GDB	Zm00001d028426_P001	70	13930	2	2	2	2	40S ribosomal protein S20-1
208	1	Zea_mays_GDB	Zm00001d009127_P001	68	66504	2	2	2	2	ATP-citrate synthase beta chain protein 2
209	1	Zea_mays_GDB	Zm00001d014093_P001	68	45390	4	4	3	3	3-ketoacyl-CoA thiolase 2 peroxisomal
210	1	Zea_mays_GDB	Zm00001d044906_P001	68	41909	1	1	1	1	12-oxo-phytodienoic acid reductase2 description
211	1	Zea_mays_GDB	Zm00001d008706_P001	68	22369	1	1	1	1	Photosystem I reaction center subunit XI chloroplastic
212	1	Zea_mays_GDB	Zm00001d026283_P001	68	34236	1	1	1	1	coatomer protein2
213	1	Zea_mays_GDB	Zm00001d016943_P001	67	24258	2	2	2	2	Photosynthetic NDH subunit of lumenal location 2 chloroplastic
214	1	Zea_mays_GDB	Zm00001d049656_P001	67	31814	2	2	2	2	Eukaryotic translation initiation factor 3 subunit G
215	1	Zea_mays_GDB	Zm00001d020201_P001	67	12951	1	1	1	1	60S ribosomal protein L35a-2
216	1	Zea_mays_GDB	Zm00001d009000_P001	67	14795	1	1	1	1	40S ribosomal protein S15a-1
217	1	Zea_mays_GDB	Zm00001d014898_P001	66	30361	4	4	3	3	Clathrin light chain 2
218	1	Zea_mays_GDB	Zm00001d002339_P001	66	17907	3	3	2	2	rps11 description
219	1	Zea_mays_GDB	Zm00001d044364_P001	66	202732	1	1	1	1	
220	1	Zea_mays_GDB	Zm00001d029543_P001	66	16313	2	2	2	2	40S ribosomal protein S19-3
221	1	Zea_mays_GDB	Zm00001d012820_P001	66	23965	3	3	3	3	Outer envelope pore protein 24A chloroplastic
222	1	Zea_mays_GDB	Zm00001d013146_P001	65	24658	2	2	1	1	Photosystem I reaction center subunit III chloroplastic
223	1	Zea_mays_GDB	Zm00001d003712_P001	65	27760	1	1	1	1	abscisic acid stress ripening3 description
224	1	Zea_mays_GDB	Zm00001d036148_P003	65	31802	2	2	2	2	whirly1 description
225	1	Zea_mays_GDB	Zm00001d038865_P001	65	16006	2	2	1	1	60S ribosomal protein L28-1
226	1	Zea_mays_GDB	Zm00001d003923_P001	65	48860	2	2	2	2	Dihydrolipoyllysine-residue succinyltransferase component of 2-oxoglutar
227	1	Zea_mays_GDB	Zm00001d011993_P001	64	22488	3	3	2	2	50S ribosomal protein L27 chloroplastic
228	1	Zea_mays_GDB	Zm00001d016542_P001	64	31700	3	3	3	3	FAD/NAD(P)-binding oxidoreductase
229	1	Zea_mays_GDB	Zm00001d003947_P002	64	116872	2	2	2	2	2-oxoglutarate dehydrogenase E1 component
230	1	Zea_mays_GDB	Zm00001d026160_P001	63	14083	2	2	2	2	gibberellin responsive2 description
231	1	Zea_mays_GDB	Zm00001d017711_P001	63	45121	3	3	3	3	Phosphoribulokinase
232	1	Zea_mays_GDB	Zm00001d036608_P001	62	65578	1	1	1	1	Glycosyl hydrolases family 31 protein
233	1	Zea_mays_GDB	Zm00001d002939_P001	62	117869	3	3	3	3	C2 calcium/lipid-binding plant phosphoribosyltransferase family protein
234	1	Zea_mays_GDB	Zm00001d031071_P001	61	39207	1	1	1	1	albino or pale green mutant1 description
235	1	Zea_mays_GDB	Zm00001d041553_P001	61	19183	1	1	1	1	Osmotin-like protein
236	1	Zea_mays_GDB	Zm00001d012992_P002	61	15181	1	1	1	1	40S ribosomal protein S26-3
237	1	Zea_mays_GDB	Zm00001d021784_P001	60	28829	5	5	2	2	Chlorophyll a-b binding protein of LHCII type III
238	1	Zea_mays_GDB	Zm00001d028240_P001	60	28390	2	2	2	2	50S ribosomal protein L15 chloroplastic
239	1	Zea_mays_GDB	Zm00001d025848_P001	60	37978	3	3	3	3	plastid transcriptionally active 5

240	1	Zea_mays_GDB	Zm00001d021310_P001	59	32686	3	3	3	3	Triosephosphate isomerase
241	1	Zea_mays_GDB	Zm00001d006100_P001	58	29543	4	4	4	4	60S ribosomal protein L7a-1
242	1	Zea_mays_GDB	Zm00001d024674_P001	58	24765	1	1	1	1	Multiple organellar RNA editing factor 9 chloroplastic
243	1	Zea_mays_GDB	Zm00001d028542_P006	57	33754	3	3	2	2	Peroxisomal nicotinamide adenine dinucleotide carrier
244	1	Zea_mays_GDB	Zm00001d046549_P002	57	109001	3	3	3	3	110 kDa U5 small nuclear ribonucleoprotein component CLO
245	1	Zea_mays_GDB	Zm00001d035981_P001	57	51276	1	1	1	1	Aspartyl protease AED1
246	1	Zea_mays_GDB	Zm00001d022124_P001	56	16099	4	4	4	4	60S ribosomal protein L27a-3
247	1	Zea_mays_GDB	Zm00001d017239_P001	56	57138	1	1	1	1	RAN GTPase-activating protein 2
248	1	Zea_mays_GDB	Zm00001d005753_P001	56	14549	2	2	2	2	60S ribosomal protein L26-1
249	1	Zea_mays_GDB	Zm00001d014858_P001	56	17136	3	3	3	3	high mobility group protein1 description
250	1	Zea_mays_GDB	Zm00001d012556_P001	55	50945	3	3	3	3	beta tubulin6 description
251	1	Zea_mays_GDB	Zm00001d022463_P001	55	14551	1	1	1	1	60S ribosomal protein L22-2
252	1	Zea_mays_GDB	Zm00001d032420_P001	55	34445	2	2	2	2	30S ribosomal protein S5 chloroplastic
253	1	Zea_mays_GDB	Zm00001d025413_P001	55	19983	3	3	3	3	40S ribosomal protein S10-1
254	1	Zea_mays_GDB	Zm00001d013918_P001	55	24152	1	1	1	1	Thylakoid lumenal 17.4 kDa protein chloroplastic
255	1	Zea_mays_GDB	Zm00001d028586_P001	55	92030	2	2	2	2	Protein TOC75-3 chloroplastic
256	1	Zea_mays_GDB	Zm00001d016358_P001	54	47243	1	1	1	1	Probable elongation factor 1-gamma 2
257	1	Zea_mays_GDB	Zm00001d026458_P001	54	51923	1	1	1	1	cysteine protease3 description
258	1	Zea_mays_GDB	Zm00001d039189_P001	54	138927	2	2	2	2	Structural maintenance of chromosomes protein 3
259	1	Zea_mays_GDB	Zm00001d035321_P001	53	15421	1	1	1	1	SWIB/MDM2 domain superfamily protein
260	1	Zea_mays_GDB	Zm00001d039542_P001	53	56532	2	2	2	2	Phospholipase A1-Igamma1 chloroplastic
261	1	Zea_mays_GDB	Zm00001d015569_P001	53	80548	2	2	2	2	Vacuolar H+-translocating inorganic pyrophosphatase
262	1	Zea_mays_GDB	Zm00001d006663_P001	53	28252	2	2	2	2	light harvesting complex A1 description
263	1	Zea_mays_GDB	Zm00001d039104_P001	53	104778	1	1	1	1	Protein CHUP1 chloroplastic
264	1	Zea_mays_GDB	Zm00001d002810_P001	52	41167	1	1	1	1	SH3 domain-containing protein 2
265	1	Zea_mays_GDB	Zm00001d018954_P002	52	23670	1	1	1	1	Rhicadhesin receptor
266	1	Zea_mays_GDB	Zm00001d038984_P001	52	14920	2	2	2	2	photosystem I H subunit1 description
267	1	Zea_mays_GDB	Zm00001d031168_P001	51	15475	1	1	1	1	glycine-rich protein1 description
268	1	Zea_mays_GDB	Zm00001d014488_P001	51	23030	1	1	1	1	50S ribosomal protein L24 chloroplastic
269	1	Zea_mays_GDB	Zm00001d037725_P001	43	33411	2	2	2	2	Mitochondrial dicarboxylate/tricarboxylate transporter DTC
270	1	Zea_mays_GDB	Zm00001d002006_P001	49	81613	1	1	1	1	plasma-membrane H+ATPase2 description
271	1	Zea_mays_GDB	Zm00001d018542_P001	49	46216	2	2	2	2	DEAD-box ATP-dependent RNA helicase 34
272	1	Zea_mays_GDB	Zm00001d001857_P001	49	27093	1	1	1	1	light harvesting complex photosystem II subunit 6
273	1	Zea_mays_GDB	Zm00001d051976_P003	48	33783	1	1	1	1	starch synthase5 description
274	1	Zea_mays_GDB	Zm00001d018410_P001	48	6927	1	1	1	1	40S ribosomal protein S30
275	1	Zea_mays_GDB	Zm00001d037239_P001	48	40844	1	1	1	1	glycine-rich protein
276	1	Zea_mays_GDB	Zm00001d027326_P001	48	49134	1	1	1	1	actin related protein like4 description
277	1	Zea_mays_GDB	Zm00001d000270_P008	48	32936	1	1	1	1	DNA-directed RNA polymerase subunit beta
278	1	Zea_mays_GDB	Zm00001d045027_P001	48	51721	1	1	1	1	3-oxoacyl-[acyl-carrier-protein] synthase I chloroplastic
279	1	Zea_mays_GDB	Zm00001d011965_P001	47	46388	1	1	1	1	Aldolase-type TIM barrel family protein
280	1	Zea_mays_GDB	Zm00001d049239_P001	47	53884	1	1	1	1	adenosyl homocysteine hydrolase1 description
281	1	Zea_mays_GDB	Zm00001d015033_P001	47	103535	1	1	1	1	phospholipase D17
282	1	Zea_mays_GDB	Zm00001d032010_P001	47	56015	1	1	1	1	26S proteasome non-ATPase regulatory subunit 3 homolog A
283	1	Zea_mays_GDB	Zm00001d039658_P001	47	25720	1	1	1	1	bZIP-transcription factor 6 description
284	1	Zea_mays_GDB	Zm00001d009146_P001	46	22614	2	2	1	1	S-adenosylmethionine synthase
285	1	Zea_mays_GDB	Zm00001d009579_P001	46	21573	2	2	2	2	60S ribosomal protein L18a
286	1	Zea_mays_GDB	Zm00001d018370_P001	46	62181	1	1	1	1	pco101682(581) description
287	1	Zea_mays_GDB	Zm00001d022421_P001	46	44688	3	3	2	2	hydroxyproline-rich glycoprotein family protein
288	1	Zea_mays_GDB	Zm00001d000260_P001	45	39762	1	1	1	1	Photosystem II D2 protein
289	1	Zea_mays_GDB	Zm00001d002258_P001	45	44406	1	1	1	1	Aminomethyltransferase
290	1	Zea_mays_GDB	Zm00001d044606_P001	45	31550	1	1	1	1	Bifunctional monothiol glutaredoxin-S16 chloroplastic
291	1	Zea_mays_GDB	Zm00001d028702_P001	45	24509	1	1	1	1	50S ribosomal protein L10 chloroplastic
292	1	Zea_mays_GDB	Zm00001d027813_P002	45	12233	1	1	1	1	40S ribosomal protein S28
293	1	Zea_mays_GDB	Zm00001d005200_P001	45	15313	1	1	1	1	40S ribosomal protein S12
294	1	Zea_mays_GDB	Zm00001d020827_P001	45	90602	1	1	1	1	Heat shock protein 90-5 chloroplastic
295	1	Zea_mays_GDB	Zm00001d038535_P001	43	16465	3	3	3	3	60S ribosomal protein L30-2
296	1	Zea_mays_GDB	Zm00001d032950_P001	43	43337	1	1	1	1	GDP-mannose 35-epimerase
297	1	Zea_mays_GDB	Zm00001d018386_P001	43	50580	2	2	2	2	glutamate-oxaloacetate transaminase2 description
298	1	Zea_mays_GDB	Zm00001d017489_P001	43	12518	1	1	1	1	50S ribosomal protein L29 chloroplastic
299	1	Zea_mays_GDB	Zm00001d024048_P001	43	46015	1	1	1	1	Argininosuccinate synthase chloroplastic
300	1	Zea_mays_GDB	Zm00001d013013_P001	43	68751	1	1	1	1	Mg chelataseD1 description
301	1	Zea_mays_GDB	Zm00001d014124_P001	43	90803	2	2	2	2	Cell division cycle protein 48
302	1	Zea_mays_GDB	Zm00001d002000_P002	43	97905	2	2	2	2	lipoxygenase6 description
303	1	Zea_mays_GDB	Zm00001d051746_P001	43	40099	1	1	1	1	Calcium sensing receptor chloroplastic
304	1	Zea_mays_GDB	Zm00001d020450_P001	42	13886	2	2	2	2	60S ribosomal protein L34-3
305	1	Zea_mays_GDB	Zm00001d010180_P001	42	24167	1	1	1	1	Thioesterase superfamily protein
306	1	Zea_mays_GDB	Zm00001d002815_P001	42	24021	2	2	2	2	NAD(P)H-quinone oxidoreductase subunit M chloroplastic
307	1	Zea_mays_GDB	Zm00001d010009_P001	42	19621	2	2	2	2	ribosomal protein L17a description
308	1	Zea_mays_GDB	Zm00001d015754_P001	42	210103	1	1	1	1	Nuclear pore complex protein NUP205
309	1	Zea_mays_GDB	Zm00001d012353_P001	41	14814	2	2	2	2	RPS17 description
310	1	Zea_mays_GDB	Zm00001d053919_P002	41	19628	1	1	1	1	Histone deacetylase complex subunit SAP18
311	1	Zea_mays_GDB	Zm00001d027371_P001	41	58096	1	1	1	1	T-complex protein 1 subunit delta
312	1	Zea_mays_GDB	Zm00001d011513_P001	41	47584	1	1	1	1	Polynucleotidyl transferase ribonuclease H-like superfamily protein
313	1	Zea_mays_GDB	Zm00001d004707_P001	41	119410	1	1	1	1	thylakoid assembly1 description
314	1	Zea_mays_GDB	Zm00001d035761_P001	40	26599	2	2	1	1	Peptidyl-prolyl cis-trans isomerase
315	1	Zea_mays_GDB	Zm00001d009364_P001	40	53567	4	4	1	1	Protein kinase and PP2C-like domain-containing protein
316	1	Zea_mays_GDB	Zm00001d036073_P002	40	32283	1	1	1	1	Hypersensitive-induced reaction protein 4
317	1	Zea_mays_GDB	Zm00001d008210_P001	40	22514	1	1	1	1	Germin-like protein subfamily 2 member 1
318	1	Zea_mays_GDB	Zm00001d002373_P002	39	109932	1	1	1	1	26S proteasome non-ATPase regulatory subunit 1 homolog B
319	1	Zea_mays_GDB	Zm00001d005151_P007	39	31593	1	1	1	1	aspartate kinase2 description
320	1	Zea_mays_GDB	Zm00001d014946_P001	39	46910	1	1	1	1	BAG family molecular chaperone regulator 7
321	1	Zea_mays_GDB	Zm00001d003373_P001	39	38454	2	2	1	1	
322	1	Zea_mays_GDB	Zm00001d029201_P001	39	25000	2	2	1	1	50S ribosomal protein L6
323	1	Zea_mays_GDB	Zm00001d016549_P001	38	14291	5	5	4	4	60S ribosomal protein L35 description
324	1	Zea_mays_GDB	Zm00001d014073_P001	34	13618	2	2	2	2	60S ribosomal protein L23
325	1	Zea_mays_GDB	Zm00001d011483_P001	39	144182	1	1	1	1	Transducin/WD40 repeat-like superfamily protein
326	1	Zea_mays_GDB	Zm00001d052723_P002	38	88366	1	1	1	1	
327	1	Zea_mays_GDB	Zm00001d017424_P001	38	51677	1	1	1	1	ATP-dependent zinc metalloprotease FTSH 7 chloroplastic
328	1	Zea_mays_GDB	Zm00001d039173_P001	38	31876	1	1	1	1	hypersensitive induced reaction3 description
329	1	Zea_mays_GDB	Zm00001d011610_P001	38	238577	1	1	1	1	Glutamate synthase 1 [NADH] chloroplastic
330	1	Zea_mays_GDB	Zm00001d002260_P001	38	33665	1	1	1	1	Peroxisomal (S)-2-hydroxy-acid oxidase GLO1
331	1	Zea_mays_GDB	Zm00001d030581_P001	38	52791	2	2	2	2	Pentatricopeptide repeat (PPR) superfamily protein
332	1	Zea_mays_GDB	Zm00001d024463_P001	38	123993	1	1	1	1	Thyroid adenoma-associated protein-like protein
333	1	Zea_mays_GDB	Zm00001d029174_P001	38	60753	1	1	1	1	homolog of nucleolar protein NOP56
334	1	Zea_mays_GDB	Zm00001d046348_P007	38	38011	1	1	1	1	Histone deacetylase 11
335	1	Zea_mays_GDB	Zm00001d048669_P001	38	70283	2	2	2	2	Auxin-induced beta-glucosidase%3B Putative O-Glycosyl hydrolase superfamily
336	1	Zea_mays_GDB	Zm00001d003189_P001	38	3252	4	4	1	1	
337	1	Zea_mays_GDB	ZemaCp062	37	17701	1	1	1	1	rpl22 description
338	1	Zea_mays_GDB	Zm00001d022457_P001	37	33762	1	1	1	1	peroxidase3 description
339	1	Zea_mays_GDB	Zm00001d032331_P001	37	46839	1	1	1	1	Peptidyl-prolyl cis-trans isomerase
340	1	Zea_mays_GDB	Zm00001d008379_P001	37	41840	1	1	1	1	cysteine synthase1 description
341	1	Zea_mays_GDB	Zm00001d053342_P003	37	23507	1	1	1	1	DUF3119 family protein
342	1	Zea_mays_GDB	Zm00001d022630_P001	37	28251	2	2	1	1	knotted1 induced1 description
343	1	Zea_mays_GDB	Zm00001d014679_P001	37	12184	1	1	1	1	acidic ribosomal protein P3 description
344	1	Zea_mays_GDB	Zm00001d015366_P001	37	46158	1	1	1	1	camouflage1 description
345	1	Zea_mays_GDB	ZemaCp022	37	82719	1	1	1	1	psaB description
346	1	Zea_mays_GDB	Zm00001d012886_P001	37	9086	1	1	1	1	40S ribosomal protein S21
347	1	Zea_mays_GDB	Zm00001d026652_P001	37	40934	1	1	1	1	DNA binding protein
348	1	Zea_mays_GDB	Zm00001d024635_P001	37	54206	1	1	1	1	Chaperone protein dnaJ A6 chloroplastic
349	1	Zea_mays_GDB	Zm00001d018901_P002	36	37360	1	1	1	1	protein containing PDZ domain a K-box domain and a TPR region
350	1	Zea_mays_GDB	Zm00001d016134_P001	36	24324	1	1	1	1	iron-sulfur protein1 description
351	1	Zea_mays_GDB	Zm00001d007898_P001	36	27694	1	1	1	1	Small nuclear ribonucleoprotein-associated protein
352	1	Zea_mays_GDB	Zm00001d015450_P001	36	51100	2	2	2	2	SUN domain protein1 description
353	1	Zea_mays_GDB	Zm00001d024903_P001	36	82037	1	1	1	1	heat shock protein, 90 kDa description
354	1	Zea_mays_GDB	ZemaCp084	36	6855	2	2	1	1	rpl32 description
355	1	Zea_mays_GDB	Zm00001d002253_P001	36	15798	1	1	1	1	60S ribosomal protein L27
356	1	Zea_mays_GDB	Zm00001d029738_P001	36	22497	1	1	1	1	Alpha/beta hydrolase related protein
357	1	Zea_mays_GDB	Zm00001d011130_P001	36	40360	1	1	1	1	Nucleosome assembly protein 1;2
358	1	Zea_mays_GDB	Zm00001d003457_P001	36	21998	1	1	1	1	PLAT domain-containing protein 3
359	1	Zea_mays_GDB	Zm00001d022134_P001	36	24951	2	2	2	2	Elongation factor 1-beta
360	1	Zea_mays_GDB	ZemaCp061	35	26128	4	4	4	4	rps3 description
361	1	Zea_mays_GDB	Zm00001d015050_P001	35	32348	1	1	1	1	Chaperone protein dnaJ 6
362	1	Zea_mays_GDB	Zm00001d002721_P001	35	14169	1	1	1	1	extensin-like protein
363	1	Zea_mays_GDB	Zm00001d041593_P001	35	46948	3	3	3	3	ATP-citrate synthase
364	1	Zea_mays_GDB	Zm00001d019328_P001	35	28833	1	1	1	1	BTB/POZ domain-containing protein
365	1	Zea_mays_GDB	Zm00001d014341_P001	35	36863	1	1	1	1	Peroxidase 53

366	1	Zea_mays_GDB	ZemaCp082	35	9438	1	1	1	1	rps15
367	1	Zea_mays_GDB	Zm00001d015385_P001	35	26520	3	3	2	2	Chlorophyll a-b binding protein 6 chloroplastic
368	1	Zea_mays_GDB	Zm00001d026501_P001	35	46344	2	2	2	2	glutamine synthetase1 description
369	1	Zea_mays_GDB	Zm00001d022513_P001	34	23450	2	2	2	2	Elongation factor 1-beta 2
370	1	Zea_mays_GDB	Zm00001d011474_P001	34	25500	1	1	1	1	Ran GTP binding protein
371	1	Zea_mays_GDB	Zm00001d015505_P001	34	32477	1	1	1	1	Nucleoid-associated protein chloroplastic
372	1	Zea_mays_GDB	Zm00001d031850_P001	33	13654	1	1	1	1	Protein PROTON GRADIENT REGULATION 5 chloroplastic
373	1	Zea_mays_GDB	Zm00001d005996_P001	33	15304	1	1	1	1	Photosystem I reaction center subunit V
374	1	Zea_mays_GDB	Zm00001d032695_P001	33	35909	4	4	3	3	malate dehydrogenase4 description
375	1	Zea_mays_GDB	Zm00001d028786_P001	33	22312	3	3	3	3	40S ribosomal protein S7
376	1	Zea_mays_GDB	Zm00001d037802_P001	33	28431	1	1	1	1	
377	1	Zea_mays_GDB	Zm00001d013111_P001	33	47216	1	1	1	1	DnaJ protein
378	1	Zea_mays_GDB	Zm00001d027366_P001	33	26521	1	1	1	1	peroxisomal membrane protein homolog1
379	1	Zea_mays_GDB	Zm00001d020487_P001	33	51009	1	1	1	1	ARM repeat superfamily protein
380	1	Zea_mays_GDB	Zm00001d043526_P001	33	10255	1	1	1	1	haloacid dehalogenase-like hydrolase family protein
381	1	Zea_mays_GDB	ZemaCp045	32	20643	2	2	1	1	rps18 description
382	1	Zea_mays_GDB	Zm00001d012771_P001	32	59811	1	1	1	1	tRNA/rRNA methyltransferase (SpoU) family protein
383	1	Zea_mays_GDB	Zm00001d017630_P001	32	31012	1	1	1	1	CBL-interacting serine/threonine-protein kinase 5
384	1	Zea_mays_GDB	Zm00001d017850_P001	32	14087	1	1	1	1	60S ribosomal protein L31
385	1	Zea_mays_GDB	Zm00001d042216_P001	32	23880	2	2	2	2	glutathione S-transferase4 description
386	1	Zea_mays_GDB	Zm00001d017401_P001	32	77071	1	1	1	1	Hsp70-Hsp90 organizing protein 3
387	1	Zea_mays_GDB	Zm00001d018412_P001	32	10040	1	1	1	1	50S ribosomal protein L9 chloroplastic
388	1	Zea_mays_GDB	Zm00001d013405_P001	32	123807	1	1	1	1	Protein kinase superfamily protein
389	1	Zea_mays_GDB	Zm00001d019454_P001	32	37548	1	1	1	1	PGR5-like protein 1B chloroplastic
390	1	Zea_mays_GDB	Zm00001d023516_P001	32	13559	1	1	1	1	Salt stress-induced protein
391	1	Zea_mays_GDB	Zm00001d040686_P001	32	127001	1	1	1	1	Protein translocase subunit SECA1 chloroplastic
392	1	Zea_mays_GDB	2::Q1RMK2	31	65815	1	1	1	1	
393	1	Zea_mays_GDB	Zm00001d034241_P001	31	37782	1	1	1	1	Malate dehydrogenase
394	1	Zea_mays_GDB	Zm00001d002450_P001	31	17821	2	2	2	2	60S ribosomal protein L12-3
395	1	Zea_mays_GDB	Zm00001d028612_P001	30	35283	1	1	1	1	Expressed protein; protein
396	1	Zea_mays_GDB	Zm00001d015765_P010	30	15184	1	1	1	1	SWR1-complex protein 4
397	1	Zea_mays_GDB	Zm00001d003590_P001	30	39792	1	1	1	1	Protein phosphatase methylesterase 1
398	1	Zea_mays_GDB	Zm00001d014138_P002	30	33059	1	1	1	1	Splicing factor CC1-like
399	1	Zea_mays_GDB	Zm00001d042620_P001	30	15507	1	1	1	1	NAD(P)H-quinone oxidoreductase subunit N chloroplastic
400	1	Zea_mays_GDB	Zm00001d047062_P001	30	55266	1	1	1	1	Ras-related protein Rab5
401	1	Zea_mays_GDB	Zm00001d039243_P001	30	42506	1	1	1	1	Receptor-like serine/threonine-protein kinase
402	1	Zea_mays_GDB	Zm00001d006588_P001	30	32018	1	1	1	1	Protein THYLAKOID FORMATION 1 chloroplastic
403	1	Zea_mays_GDB	Zm00001d035871_P001	30	49159	1	1	1	1	GDSL esterase/lipase
404	1	Zea_mays_GDB	Zm00001d009929_P001	29	30899	1	1	1	1	Beta-propeller domain of methanol dehydrogenase type%3B Beta-propeller do
405	1	Zea_mays_GDB	Zm00001d028560_P001	29	46728	1	1	1	1	LRR receptor-like serine/threonine-protein kinase FEI 1
406	1	Zea_mays_GDB	ZemaCp092	29	45829	1	1	1	1	ndhH description
407	1	Zea_mays_GDB	Zm00001d019926_P001	29	39455	1	1	1	1	Enoyl-[acyl-carrier-protein] reductase [NADH] chloroplastic
408	1	Zea_mays_GDB	Zm00001d036772_P002	29	28388	1	1	1	1	
409	1	Zea_mays_GDB	Zm00001d027442_P001	29	30551	1	1	1	1	50S ribosomal protein L5
410	1	Zea_mays_GDB	Zm00001d052854_P001	29	53846	1	1	1	1	Pentatricopeptide repeat-containing protein mitochondrial
411	1	Zea_mays_GDB	Zm00001d029924_P001	28	48613	1	1	1	1	Flavin-containing monooxygenase FMO GS-OX5
412	1	Zea_mays_GDB	Zm00001d009761_P001	28	49188	1	1	1	1	Spliceosome RNA helicase BAT1 isoform 1%3B Spliceosome RNA helicase BAT1
413	1	Zea_mays_GDB	Zm00001d033665_P001	28	20904	1	1	1	1	30S ribosomal protein S13 chloroplastic
414	1	Zea_mays_GDB	Zm00001d005707_P002	27	75237	1	1	1	1	AUGMIN subunit 5
415	1	Zea_mays_GDB	Zm00001d002896_P001	27	50041	1	1	1	1	TUB-transcription factor 4 description
416	1	Zea_mays_GDB	Zm00001d010690_P001	27	50197	1	1	1	1	Anthocyanidin 3-O-glucosyltransferase
417	1	Zea_mays_GDB	Zm00001d004342_P001	27	41350	2	2	2	2	Natterin-4
418	1	Zea_mays_GDB	Zm00001d034015_P001	27	53725	2	2	2	2	exoglucanase1 description
419	1	Zea_mays_GDB	Zm00001d031847_P001	27	141488	1	1	1	1	Structural maintenance of chromosomes (SMC) family protein
420	1	Zea_mays_GDB	Zm00001d044208_P001	27	65062	1	1	1	1	P-loop containing nucleoside triphosphate hydrolases superfamily prote
421	1	Zea_mays_GDB	Zm00001d024977_P001	26	17118	1	1	1	1	Disease resistance protein RPM1
422	1	Zea_mays_GDB	Zm00001d012103_P001	26	38891	1	1	1	1	aldolase2 description
423	1	Zea_mays_GDB	Zm00001d006756_P001	26	17391	1	1	1	1	WPP domain-containing protein 2
424	1	Zea_mays_GDB	Zm00001d027291_P001	26	14965	1	1	1	1	extensin-like protein
425	1	Zea_mays_GDB	Zm00001d016822_P001	25	68556	1	1	1	1	Phosphoinositide phosphatase SAC6
426	1	Zea_mays_GDB	Zm00001d019266_P001	25	56661	1	1	1	1	ADP glucose pyrophosphorylase large subunit leaf2 description
427	1	Zea_mays_GDB	Zm00001d042289_P001	25	40215	1	1	1	1	Rieske domain containing protein
428	1	Zea_mays_GDB	Zm00001d020929_P001	25	88597	1	1	1	1	
429	1	Zea_mays_GDB	Zm00001d010617_P001	25	35307	2	2	1	1	WRKY-transcription factor 44 description
430	1	Zea_mays_GDB	Zm00001d003257_P001	25	44211	1	1	1	1	26S proteasome non-ATPase regulatory subunit 6 homolog
431	1	Zea_mays_GDB	Zm00001d007215_P001	25	51628	1	1	1	1	26S proteasome regulatory subunit 4 homolog A
432	1	Zea_mays_GDB	Zm00001d043625_P001	25	40409	1	1	1	1	Plasminogen activator inhibitor 1 RNA-binding protein
433	1	Zea_mays_GDB	Zm00001d035212_P001	24	35994	2	2	2	2	Chloroplast stem-loop binding protein of 41 kDa b chloroplastic
434	1	Zea_mays_GDB	Zm00001d027510_P001	24	106568	1	1	1	1	NLP-transcription factor 7 description
435	1	Zea_mays_GDB	Zm00001d052843_P001	24	27533	1	1	1	1	Caffeoyl-CoA O-methyltransferase 2
436	1	Zea_mays_GDB	Zm00001d005231_P001	24	21298	1	1	1	1	ADP-ribosylation factor A1F
437	1	Zea_mays_GDB	Zm00001d000327_P001	24	15328	1	1	1	1	Cysteine protease 1
438	1	Zea_mays_GDB	Zm00001d006034_P007	24	26745	1	1	1	1	DNA binding protein
439	1	Zea_mays_GDB	Zm00001d009583_P001	24	14042	1	1	1	1	
440	1	Zea_mays_GDB	Zm00001d023496_P001	24	16451	1	1	1	1	
441	1	Zea_mays_GDB	Zm00001d026041_P004	23	125959	1	1	1	1	ABC transporter C family member 9
442	1	Zea_mays_GDB	Zm00001d015837_P001	23	71732	1	1	1	1	Outer envelope protein 80 chloroplastic
443	1	Zea_mays_GDB	Zm00001d034721_P001	23	80521	1	1	1	1	RNA helicase4 description
444	1	Zea_mays_GDB	Zm00001d014082_P001	23	18894	1	1	1	1	60S ribosomal protein L21-1
445	1	Zea_mays_GDB	Zm00001d012751_P059	23	89368	1	1	1	1	Homeobox-transcription factor 9 description
446	1	Zea_mays_GDB	Zm00001d044287_P001	23	24156	1	1	1	1	Ribosomal protein L19
447	1	Zea_mays_GDB	Zm00001d050955_P001	23	56077	1	1	1	1	Putative cytochrome P450 superfamily protein
448	1	Zea_mays_GDB	Zm00001d039921_P001	23	58203	3	3	1	1	PR5-like receptor kinase
449	1	Zea_mays_GDB	Zm00001d032967_P001	23	39680	1	1	1	1	
450	1	Zea_mays_GDB	Zm00001d003695_P001	23	55079	2	2	1	1	
451	1	Zea_mays_GDB	Zm00001d015992_P001	22	132169	1	1	1	1	
452	1	Zea_mays_GDB	Zm00001d038547_P001	22	50361	1	1	1	1	Glutamate-1-semialdehyde 21-aminomutase 2 chloroplastic
453	1	Zea_mays_GDB	Zm00001d040582_P001	22	17172	1	1	1	1	Probable tRNA N6-adenosine threonylcarbamoyltransferase mitochondrial
454	1	Zea_mays_GDB	Zm00001d037171_P001	22	15586	1	1	1	1	Galactoside 2-alpha-L-fucosyltransferase
455	1	Zea_mays_GDB	Zm00001d021912_P001	22	84367	2	2	1	1	subtilisin3 description
456	1	Zea_mays_GDB	Zm00001d046719_P015	22	43576	1	1	1	1	ARID-transcription factor 10 description
457	1	Zea_mays_GDB	Zm00001d042262_P005	22	26613	1	1	1	1	Retrovirus-related Pol polyprotein LINE-1
458	1	Zea_mays_GDB	Zm00001d039758_P002	21	195469	1	1	1	1	ARM repeat superfamily protein
459	1	Zea_mays_GDB	Zm00001d044815_P001	21	33701	1	1	1	1	Protein BOBBER 1
460	1	Zea_mays_GDB	Zm00001d007581_P001	21	29408	1	1	1	1	harpin binding protein1
461	1	Zea_mays_GDB	Zm00001d008982_P001	21	30869	1	1	1	1	Reticulon-like protein B4
462	1	Zea_mays_GDB	Zm00001d038924_P001	21	38357	1	1	1	1	Galactosyltransferase family protein
463	1	Zea_mays_GDB	Zm00001d009452_P001	21	62961	1	1	1	1	Putative clathrin assembly protein
464	1	Zea_mays_GDB	Zm00001d052344_P002	21	11640	1	1	1	1	PAP/OAS1 substrate-binding domain superfamily
465	1	Zea_mays_GDB	Zm00001d015120_P001	21	50233	1	1	1	1	RuvB-like protein 1
466	1	Zea_mays_GDB	Zm00001d034479_P001	21	27868	1	1	1	1	histone one (H1) description
467	1	Zea_mays_GDB	Zm00001d023343_P001	20	12199	1	1	1	1	phospholipid transfer protein homolog1 description
468	1	Zea_mays_GDB	Zm00001d026215_P001	20	38566	1	1	1	1	
469	1	Zea_mays_GDB	Zm00001d030559_P001	20	86156	1	1	1	1	Callose synthase 11
470	1	Zea_mays_GDB	Zm00001d007810_P003	20	13353	1	1	1	1	40S ribosomal protein S15-4
471	1	Zea_mays_GDB	Zm00001d048540_P002	20	120767	1	1	1	1	Phosphatidylinositol N-acetyglucosaminlytransferase subunit P-related
472	1	Zea_mays_GDB	ZemaCp065	20	30245	1	1	1	1	rpl2
473	1	Zea_mays_GDB	Zm00001d029173_P003	20	19099	1	1	1	1	Nascent polypeptide-associated complex alpha subunit-like protein
474	1	Zea_mays_GDB	Zm00001d019899_P001	20	35365	1	1	1	1	Rhodanese-like domain-containing protein 9 chloroplastic
475	1	Zea_mays_GDB	Zm00001d015772_P001	20	65254	2	2	1	1	Putative UPF0481 protein
476	1	Zea_mays_GDB	Zm00001d006947_P001	20	59699	1	1	1	1	Cytochrome P450 CYP709C14
477	1	Zea_mays_GDB	Zm00001d032567_P001	20	137775	1	1	1	1	Mediator of RNA polymerase II transcription subunit 16
478	1	Zea_mays_GDB	Zm00001d009824_P002	20	37870	1	1	1	1	OSJNBa0059D20.8 protein; protein
479	1	Zea_mays_GDB	Zm00001d035818_P001	19	91322	1	1	1	1	Protein kinase superfamily protein with octicosapeptide/Phox/Bem1p domain
480	1	Zea_mays_GDB	Zm00001d042842_P003	19	106134	1	1	1	1	starch phoshorylase2 description
481	1	Zea_mays_GDB	Zm00001d026073_P001	19	12888	1	1	1	1	
482	1	Zea_mays_GDB	Zm00001d036401_P001	19	92572	1	1	1	1	Endoplasmin homolog
483	1	Zea_mays_GDB	Zm00001d035034_P001	19	31564	2	2	2	2	adenylate kinase1 description
484	1	Zea_mays_GDB	Zm00001d015100_P001	19	58493	1	1	1	1	Calcium-dependent protein kinase 28
485	1	Zea_mays_GDB	Zm00001d014258_P002	19	97605	1	1	1	1	alanine aminotransferase5 description
486	1	Zea_mays_GDB	Zm00001d039306_P001	19	81741	1	1	1	1	MYB-transcription factor 117 description
487	1	Zea_mays_GDB	Zm00001d016815_P001	19	67196	1	1	1	1	Pentatricopeptide repeat-containing protein
488	1	Zea_mays_GDB	Zm00001d019353_P001	19	57266	1	1	1	1	protein kinase domain containing protein expressed
489	1	Zea_mays_GDB	Zm00001d026703_P001	19	20281	1	1	1	1	Thylakoidal processing peptidase 1 chloroplastic
490	1	Zea_mays_GDB	Zm00001d011976_P001	19	19690	1	1	1	1	
491	1	Zea_mays_GDB	Zm00001d011192_P001	18	128347	1	1	1	1	Carbamoyl-phosphate synthase large chain chloroplastic

492	1	Zea_mays_GDB	Zm00001d032861_P001	18	35678	1	1	1	1	B-block binding subunit of TFIIIC
493	1	Zea_mays_GDB	Zm00001d010758_P001	18	79923	1	1	1	1	homeobox1 description
494	1	Zea_mays_GDB	Zm00001d007194_P001	18	11218	1	1	1	1	Calmodulin-7
495	1	Zea_mays_GDB	Zm00001d046304_P001	18	78033	1	1	1	1	RING/FYVE/PHD-type zinc finger family protein
496	1	Zea_mays_GDB	Zm00001d010178_P001	18	54501	1	1	1	1	Protein kinase superfamily protein
497	1	Zea_mays_GDB	Zm00001d033383_P001	18	67580	1	1	1	1	hydroxymethylpyrimidine phosphate synthase1 description
498	1	Zea_mays_GDB	Zm00001d051924_P002	18	29986	1	1	1	1	Haloacid dehalogenase-like hydrolase domain-containing protein
499	1	Zea_mays_GDB	Zm00001d008932_P001	18	33492	1	1	1	1	SEC13 protein isoform 1%3B SEC13 protein isoform 2%3B SEC13-related prote
500	1	Zea_mays_GDB	Zm00001d035561_P001	18	34392	1	1	1	1	Expressed protein; Mannose-specific jacalin-related lectin; protein
501	1	Zea_mays_GDB	Zm00001d011846_P001	18	38263	1	1	1	1	Calcium-dependent lipid-binding (CaLB domain) family protein
502	1	Zea_mays_GDB	Zm00001d019357_P002	18	40593	1	1	1	1	
503	1	Zea_mays_GDB	Zm00001d025134_P001	18	102306	1	1	1	1	
504	1	Zea_mays_GDB	Zm00001d000444_P001	18	30938	1	1	1	1	
505	1	Zea_mays_GDB	Zm00001d052452_P001	18	17779	1	1	1	1	KOW domain-containing protein
506	1	Zea_mays_GDB	Zm00001d048876_P087	18	36536	1	1	1	1	OSJNBa0016N04.16 protein; OSJNBb0042I07.4 protein
507	1	Zea_mays_GDB	Zm00001d029839_P002	18	50141	1	1	1	1	Protein NEN2
508	1	Zea_mays_GDB	Zm00001d011700_P014	17	106863	1	1	1	1	Protein kinase superfamily protein
509	1	Zea_mays_GDB	Zm00001d039346_P001	17	65956	1	1	1	1	Indole-3-acetic acid-amido synthetase GH3.6
510	1	Zea_mays_GDB	Zm00001d018457_P001	17	12774	1	1	1	1	Probable dolichyl pyrophosphate Man9GlcNAc2 alpha-13-glucosyltransferas
511	1	Zea_mays_GDB	Zm00001d012998_P001	17	22818	1	1	1	1	50S ribosomal protein L17 chloroplastic
512	1	Zea_mays_GDB	Zm00001d037955_P001	17	13973	2	2	1	1	
513	1	Zea_mays_GDB	Zm00001d020644_P001	17	28658	1	1	1	1	Mediator of RNA polymerase II transcription subunit 20a
514	1	Zea_mays_GDB	Zm00001d028875_P001	17	81147	1	1	1	1	Lon protease homolog 2 peroxisomal
515	1	Zea_mays_GDB	Zm00001d035429_P001	17	75268	1	1	1	1	Retrovirus-related Pol polyprotein LINE-1
516	1	Zea_mays_GDB	Zm00001d006185_P001	17	34874	1	1	1	1	6-phosphogluconolactonase
517	1	Zea_mays_GDB	Zm00001d018407_P001	17	42845	1	1	1	1	
518	1	Zea_mays_GDB	Zm00001d032980_P016	17	76788	1	1	1	1	Phospholipid-transporting ATPase 3
519	1	Zea_mays_GDB	Zm00001d036835_P001	17	34108	1	1	1	1	Peroxidase 67
520	1	Zea_mays_GDB	Zm00001d002726_P004	16	14802	1	1	1	1	Pectinesterase
521	1	Zea_mays_GDB	Zm00001d053876_P003	16	88320	1	1	1	1	Protein kinase protein with adenine nucleotide alpha hydrolases-like d
522	1	Zea_mays_GDB	Zm00001d006396_P001	16	23954	1	1	1	1	
523	1	Zea_mays_GDB	Zm00001d003911_P004	16	19658	1	1	1	1	Protein AUXIN SIGNALING F-BOX 3
524	1	Zea_mays_GDB	Zm00001d037258_P001	16	16257	1	1	1	1	ROS1 protein
525	1	Zea_mays_GDB	Zm00001d047888_P001	16	10121	1	1	1	1	Catalytic/ oxidoreductase%2C acting on NADH or NADPH
526	1	Zea_mays_GDB	Zm00001d033734_P002	16	121037	2	2	1	1	Coatomer subunit alpha-1
527	1	Zea_mays_GDB	Zm00001d029106_P001	16	38624	1	1	1	1	Peter Pan-like protein
528	1	Zea_mays_GDB	Zm00001d009948_P001	16	92989	1	1	1	1	Heat shock 70 kDa protein 14
529	1	Zea_mays_GDB	Zm00001d031895_P001	16	45538	1	1	1	1	C2H2 and C2HC zinc fingers superfamily protein
530	1	Zea_mays_GDB	Zm00001d016164_P001	16	49034	1	1	1	1	GW2
531	1	Zea_mays_GDB	Zm00001d004821_P001	16	11364	1	1	1	1	V-type proton ATPase 16 kDa proteolipid subunit
532	1	Zea_mays_GDB	Zm00001d011706_P003	16	52649	1	1	1	1	Probable mitochondrial-processing peptidase subunit beta mitochondrial
533	1	Zea_mays_GDB	Zm00001d043852_P001	16	94092	1	1	1	1	
534	1	Zea_mays_GDB	Zm00001d034084_P001	16	45368	1	1	1	1	WRKY-transcription factor 31 description
535	1	Zea_mays_GDB	Zm00001d019561_P002	16	109315	1	1	1	1	SUPPRESSOR OF ABI3-5
536	1	Zea_mays_GDB	Zm00001d034705_P001	16	11422	1	1	1	1	Putative uncharacterized protein [Source
537	1	Zea_mays_GDB	Zm00001d010314_P001	16	37414	1	1	1	1	
538	1	Zea_mays_GDB	Zm00001d012036_P001	16	119298	1	1	1	1	sucrose phosphate synthase1 description
539	1	Zea_mays_GDB	Zm00001d042792_P001	16	47831	1	1	1	1	Transcription initiation factor TFIID subunit 12
540	1	Zea_mays_GDB	Zm00001d031351_P001	15	94557	1	1	1	1	Protein kinase superfamily protein
541	1	Zea_mays_GDB	Zm00001d023919_P001	15	106742	1	1	1	1	Protein STABILIZED1
542	1	Zea_mays_GDB	Zm00001d012821_P001	15	65546	1	1	1	1	Probable inactive poly [ADP-ribose] polymerase SRO1
543	1	Zea_mays_GDB	Zm00001d054011_P002	15	53625	1	1	1	1	Zn-dependent exopeptidases superfamily protein
544	1	Zea_mays_GDB	Zm00001d032784_P001	15	38553	1	1	1	1	G2-like-transcription factor 45 description
545	1	Zea_mays_GDB	Zm00001d047762_P002	15	97447	1	1	1	1	Ypt/Rab-GAP domain of gyp1p superfamily protein
546	1	Zea_mays_GDB	Zm00001d012818_P001	15	14853	1	1	1	1	Acyl carrier protein 3
547	1	Zea_mays_GDB	Zm00001d032604_P001	15	49074	1	1	1	1	WAT1-related protein chloroplastic
548	1	Zea_mays_GDB	Zm00001d041269_P001	15	106732	1	1	1	1	Probable serine/threonine protein kinase IRE
549	1	Zea_mays_GDB	Zm00001d021802_P001	15	51581	1	1	1	1	ARF GAP-like zinc finger-containing protein ZIGA3
550	1	Zea_mays_GDB	Zm00001d027835_P001	15	105187	1	1	1	1	Pentatricopeptide repeat-containing protein
551	1	Zea_mays_GDB	Zm00001d038884_P001	15	50744	1	1	1	1	Putative MAPKKK family protein kinase
552	1	Zea_mays_GDB	Zm00001d015204_P001	15	27850	1	1	1	1	50S ribosomal protein L3-1 chloroplastic
553	1	Zea_mays_GDB	Zm00001d001766_P001	15	33620	1	1	1	1	
554	1	Zea_mays_GDB	Zm00001d040694_P001	15	94382	1	1	1	1	
555	1	Zea_mays_GDB	Zm00001d007732_P004	15	20733	1	1	1	1	3-hydroxyisobutyryl-CoA hydrolase 1
556	1	Zea_mays_GDB	Zm00001d009638_P001	14	63213	1	1	1	1	Ketol-acid reductoisomerase chloroplastic
557	1	Zea_mays_GDB	Zm00001d040294_P001	14	15789	1	1	1	1	Sm-like protein LSM4
558	1	Zea_mays_GDB	Zm00001d032659_P012	14	12067	1	1	1	1	Protein EXECUTER 1 chloroplastic
559	1	Zea_mays_GDB	Zm00001d017505_P017	14	12907	1	1	1	1	Homeodomain-like superfamily protein
560	1	Zea_mays_GDB	Zm00001d041145_P001	14	173049	1	1	1	1	
561	1	Zea_mays_GDB	Zm00001d032062_P001	14	40446	1	1	1	1	Mitochondrial import inner membrane translocase subunit Tim17/Tim22/Tim
562	1	Zea_mays_GDB	Zm00001d024274_P002	14	44614	1	1	1	1	Obg-like ATPase 1
563	1	Zea_mays_GDB	Zm00001d017784_P001	14	45202	1	1	1	1	ZF-HD-transcription factor 12 description
564	1	Zea_mays_GDB	Zm00001d003857_P001	14	60592	1	1	1	1	calnexin homolog1 description

Significance threshold $P < 0.05$; Minimum number of significant unique sequences=1.

Supplementary Table 5. The phosphoproteomic analysis results of ZmBLK1 and ZmRBOH4.

6 hours post inoculation of *C. zeina*

Gene name	Protein accession	Position	Amino acid*	Q_CZ_6/ Y_CZ_6 Ratio	Q_CZ_6/ Y_CZ_6 P value	Regulated Type	Protein description	Gene name	Localization probability	PEP	Score	Charge	Modified sequence	Mass error [ppm]	Intensity Y_CZ_6_1	Intensity Y_CZ_6_2	Intensity Y_CZ_6_3	Intensity Q_CZ_6_1	Intensity Q_CZ_6_2	Intensity Q_CZ_6_3
ZmBLK1	Zm00001d034662_P001	13	S	0.860247	0.202889	None	Zm00001d034662 GN=Zm00001d034662	Zm00001d034662	0.999901	2.45E-08	101.29	3	IKDGS(1)PHPGASGMFSK	-0.94935	162240	171140	150760	152520	152340	111620
	Zm00001d034662_P001	80	S	0.904653	0.150905	None	Zm00001d034662 GN=Zm00001d034662	Zm00001d034662	1	2.34E-19	126.04	3	NFRPDS(1)VLGEGGFGSVYK	0.97982	82460	77801	68682	69788	69657	67669
ZmRBOH4	Zm00001d052653_P004	28	S	0.909226	0.257008	None	Zm00001d052653 GN=Zm00001d052653	Zm00001d052653	0.992464	0.002014	62.924	3	VIPHS(0.992)GPLS(0.008)K	0.37975	22152	22721	22547	22671	21135	17494
	Zm00001d052653_P004	32	S	0.828493	0.17577	None	Zm00001d052653 GN=Zm00001d052653	Zm00001d052653	0.998113	0.000517	86.803	3	VIPHS(0.002)GPLS(0.998)K	0.45769	10669	8867.4	8468.3	8005.4	6406.8	8789.5
	Zm00001d052653_P004	48	S	0.996695	0.963532	None	Zm00001d052653 GN=Zm00001d052653	Zm00001d052653	0.940524	0.000147	75.143	3	FAES(0.059)VS(0.941)APLSAPPPR	0.77412	7254.6	8338.6	8100	8609.1	7865.1	7140.7
	Zm00001d052653_P004	121	S	0.865754	0.006799	None	Zm00001d052653 GN=Zm00001d052653	Zm00001d052653	0.984873	0.000925	100.09	2	RS(0.985)S(0.01)S(0.005)YGHSVIR	1.0852	16451	16635	15772	14673	13513	14113
	Zm00001d052653_P004	148	S	0.770177	0.078394	None	Zm00001d052653 GN=Zm00001d052653	Zm00001d052653	1	0.019253	53.493	3	IAS(1)INRR	-0.06791	13376	15749	14563	13706	9832.5	10109
	Zm00001d052653_P004	344	S	0.853954	0.057417	None	Zm00001d052653 GN=Zm00001d052653	Zm00001d052653	0.928928	0.005781	46.656	3	NLS(0.929)QMLS(0.046)QS(0.022)LRPT(0.003)AEPNPLR	0.1982	26704	24742	22469	20173	22185	20762
	Zm00001d052653_P004	348	S	0.745589	0.110018	None	Zm00001d052653 GN=Zm00001d052653	Zm00001d052653	0.988593	1.95E-32	167.28	3	NLSQMLS(0.989)QS(0.011)LRPTAEPNPLR	1.0829	127440	99622	98341	87474	93126	62017

Source Data of Supplementary Figures

Supplementary Fig. 1a

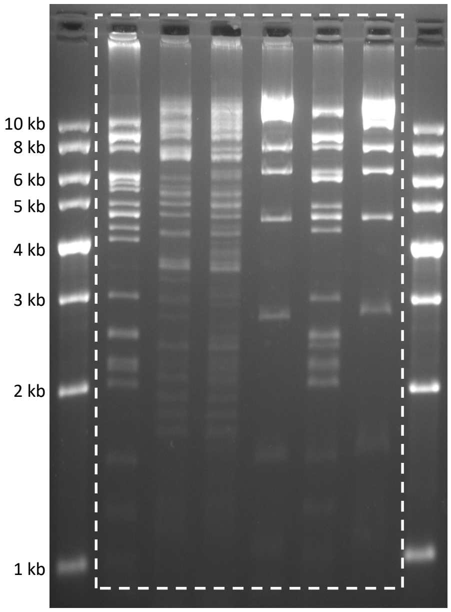

Supplementary Fig. 1d

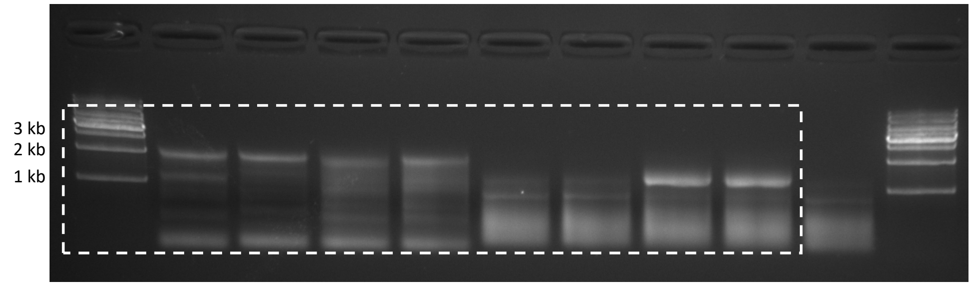

Supplementary Fig. 1e

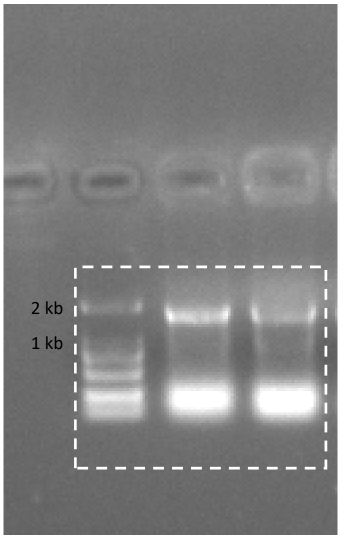

Supplementary Fig. 6b

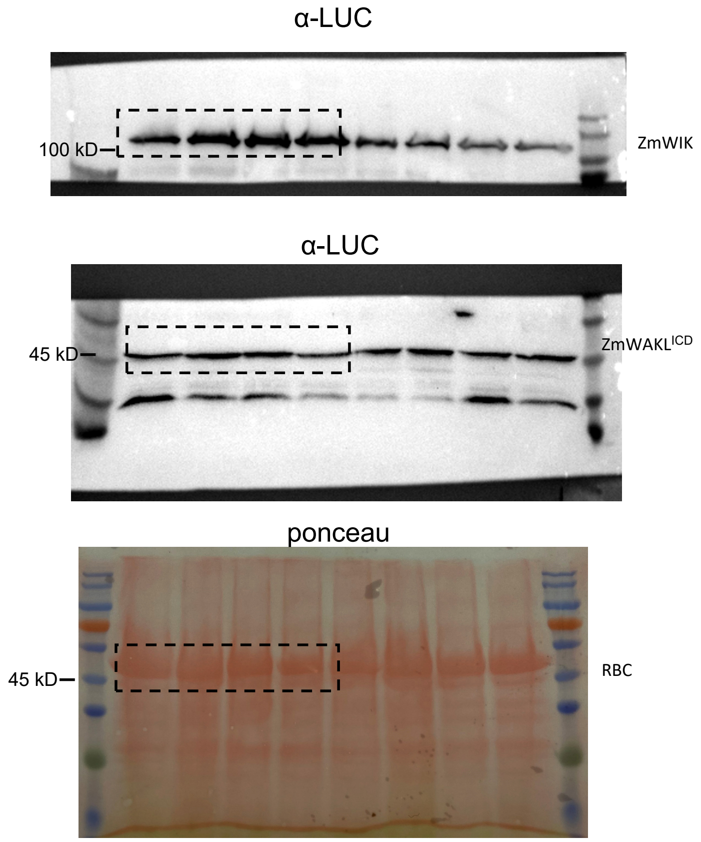

Supplement: Supplementary file 1 — Supplementary Figs. 1–8, Supplementary Methods, Supplementary Tables 1–5 and Supporting data for Supplementary Figs. 1 and 6. [file 41588_2023_1644_MOESM1_ESM.pdf]
