# Supplementary material for: The ZmWAKL–ZmWIK–ZmBLK1–ZmRBOH4 module provides quantitative resistance to gray leaf spot in maize
Source: Nat Genet. 2024 Jan 18;56(2):315–26. doi: 10.1038/s41588-023-01644-z (PMC10864183; doi:10.1038/s41588-023-01644-z)
Supplement: Supplementary file 2 — Reporting Summary [file 41588_2023_1644_MOESM2_ESM.pdf]

nature portfolio

Corresponding author(s): Mingliang Xu

Last updated by author(s): Nov 30, 2023



# Reporting Summary

Nature Portfolio wishes to improve the reproducibility of the work that we publish. This form provides structure for consistency and transparency in reporting. For further information on Nature Portfolio policies, see our Editorial Policies and the Editorial Policy Checklist.

## Statistics

For all statistical analyses, confirm that the following items are present in the figure legend, table legend, main text, or Methods section.

| n/a | Confirmed | |
|---|---|---|
| ☐ | ☒ | The exact sample size (*n*) for each experimental group/condition, given as a discrete number and unit of measurement |
| ☐ | ☒ | A statement on whether measurements were taken from distinct samples or whether the same sample was measured repeatedly |
| ☐ | ☒ | The statistical test(s) used AND whether they are one- or two-sided<br>*Only common tests should be described solely by name; describe more complex techniques in the Methods section.* |
| ☒ | ☐ | A description of all covariates tested |
| ☒ | ☐ | A description of any assumptions or corrections, such as tests of normality and adjustment for multiple comparisons |
| ☐ | ☒ | A full description of the statistical parameters including central tendency (e.g. means) or other basic estimates (e.g. regression coefficient) AND variation (e.g. standard deviation) or associated estimates of uncertainty (e.g. confidence intervals) |
| ☐ | ☒ | For null hypothesis testing, the test statistic (e.g. *F*, *t*, *r*) with confidence intervals, effect sizes, degrees of freedom and *P* value noted<br>*Give P values as exact values whenever suitable.* |
| ☒ | ☐ | For Bayesian analysis, information on the choice of priors and Markov chain Monte Carlo settings |
| ☒ | ☐ | For hierarchical and complex designs, identification of the appropriate level for tests and full reporting of outcomes |
| ☒ | ☐ | Estimates of effect sizes (e.g. Cohen's *d*, Pearson's *r*), indicating how they were calculated |

*Our web collection on statistics for biologists contains articles on many of the points above.*

## Software and code

Policy information about availability of computer code

| | |
|---|---|
| Data collection | The ImageJ 1.53c (https://imagej.net/ij/ij/download.html) was used to evaluate the gray value of western blotting and luminescence intensity. |
| Data analysis | For gene prediction, http://linux1.softberry.com/berry.phtml. For gene annotation, web based Blast2GO (https://www.blast2go.com). For predicting conserved domains, https://www.ncbi.nlm.nih.gov/Structure/cdd/wrpsb.cgi. For phylogenetic analysis, MEGA 7.0 (https://www.megasoftware.net/). For nucleotide diversity, ClustalX2 (http://www.clustal.org/) was used to produce a nucleotide alignment matrix, and DnaSP6 (http://www.ub.edu/dnasp/DnaSP_OS.html) was used for nucleotide diversity (π) analysis, Tajima's D test and haplotype analysis. For statistical analysis, performed by IBM SPSS Statistics SV26 (https://www.ibm.com/products/spss-statistics). For data visualization, GraphPad Prism 8 (https://www.graphpad.com/). |

For manuscripts utilizing custom algorithms or software that are central to the research but not yet described in published literature, software must be made available to editors and reviewers. We strongly encourage code deposition in a community repository (e.g. GitHub). See the Nature Portfolio guidelines for submitting code & software for further information.

## Data

Policy information about availability of data

All manuscripts must include a data availability statement. This statement should provide the following information, where applicable:

- Accession codes, unique identifiers, or web links for publicly available datasets
- A description of any restrictions on data availability
- For clinical datasets or third party data, please ensure that the statement adheres to our policy

The authors declare that the data supporting the findings of this study are available within the paper and its supplementary information files. The reported WAKs/WAKLs and RLCKs' protein sequences are downloaded from the National Center for Biotechnology Information database (NCBI, http://www.ncbi.nlm.nih.gov/). The protein sequences of AtRBOHs are downloaded from The Arabidopsis Information Resource database (TAIR, https://www.arabidopsis.org/), and the protein sequences of ZmRBOHs are obtained from the Gramene database (https://www.gramene.org/). The expression data of ZmRBOHs and receptor-like kinases is obtained from the Plant Public RNA-seq Database (http://ipf.sustech.edu.cn/pub/plantrna/). The B73 genomic sequences in the mapped qRgls1 region are collected from the MaizeGDB (https://www.maizegdb.org/). The sequences of two BAC clones (17-37-1-53 and 57-9-1-93) are available at GenBank accessions OQ435908 and OQ435909, respectively. The genomic sequences of ZmWAKL used for haplotype analysis are available at GenBank under accessions OQ425304-OQ425401. The coding sequences of ZmWAKLY and ZmWAKLQ are available at GenBank accessions OQ421108 and OQ421109, respectively. The genomic and coding sequences of ZmPR5Y and ZmPR5Q are available at GenBank accessions OQ421106-OQ421107 and OQ421110-OQ421111, respectively. The coding sequence of ZmWIK is available at GenBank accession OQ421112. The coding sequences of ZmBLK1 and ZmBLK1-1 are available at GenBank accessions OQ421113 and OQ421114, respectively. The N-terminal of ZmRBOH4 is available at GenBank accession OQ421115. Source data are provided with this paper.

## Human research participants

Policy information about studies involving human research participants and Sex and Gender in Research.

| | |
|---|---|
| Reporting on sex and gender | NA |
| Population characteristics | NA |
| Recruitment | NA |
| Ethics oversight | NA |

Note that full information on the approval of the study protocol must also be provided in the manuscript.

# Field-specific reporting

Please select the one below that is the best fit for your research. If you are not sure, read the appropriate sections before making your selection.

☒ Life sciences ☐ Behavioural & social sciences ☐ Ecological, evolutionary & environmental sciences

For a reference copy of the document with all sections, see nature.com/documents/nr-reporting-summary-flat.pdf

# Life sciences study design

All studies must disclose on these points even when the disclosure is negative.

| | |
|---|---|
| Sample size | Sample size are indicated in individual figures and figure legends. The sample size for homozygous lines with/without the Y32 fragment is 1151 plants, while the fine-mapping populations consist of 3290 plants. For the NIL's flowering-related traits, there are 5 repeats with 195 plants in Beijing, and 3 repeats with 96 plants in Hainan; for the transgenic materials' flowering-related traits, the complementary materials have 3 repeats with 235 plants, and the overexpression materials have 3 repeats with 353 plants. In transgenic verification experiments, the sample sizes are as follows: 790 plants for complementation assays, 1765 plants for ZmWAKLY overexpression, 852 plants for ZmWAKLQ overexpression, 918 plants for ZmWAKLC overexpression, and for ZmPR5L overexpression and CRISPR/Cas9 knockout, 365 and 148 plants respectively. ZmWIK overexpression and mutation lines have 265 and 132 plants, respectively. Lastly, the ZmRBOH4 knockout involves 183 plants. For RT-qPCR, each leaf tissue had three samples, and each sample was harvested from three plants. RNA expression for each sample was tested with three technical replicates. For oxidative burst assay, each sample had two strips with at least 6 replicates. For the protein content and interaction strength analysis, 8 group samples were used for testing. Statistical between two groups were analyzed by two-sided student's t-test or paired t-test. Statistical significance between more than two groups were analyzed based on one-way ANOVA with Tukey's test or Fisher's least significant difference (LSD) test, different lowercase letters indicate a significant difference ($P < 0.05$). |
| Data exclusions | No data were excluded from the analyses. |
| Replication | The phenotype of recombinants and NILs were identified over several years with the similar results. In the transgenic verification assays, we have identified the phenotype of transgenic materials for more than 2 years and tested the gene's function in different genetic backgrounds, and similar results were obtained across different years and generations. If we have sufficient materials, we set up at least 3 replicates every year. For the flowering-related traits experiments of NILs, we set up two locations in Beijing and Hainan, with 5 repeats in Beijing and 3 repeats in Hainan; for the transgenic flowering-related assays, 3 repeats were set up. In the split-luciferase complementation assays, more |

than three leaves (three replicates) were used for each assay. In other protein interaction assays (pull down, co-immunoprecipitation and SLC), at least three replicates were set up for each experiment. For the in vitro and in vivo phosphorylation assay, we repeated at least two times. All the replications have the similar results, and all the attempts were successful.

Randomization

Briefly, the samples were allocated into experimental groups randomly. In the fine-mapping of qRgls1, as all the recombinant-derived progeny was grown in the same plot, therefore, all individuals, with or without qRgls1, were randomly distributed in the same experimental plot. All the recombinants and replications were randomly planted in the field. For the transgenic verification assays, we adopted the same strategy. The segregating progeny of a transgenic event were randomly distributed in the same plot and the same transgenic event was randomly planted in the field. The homozygous transgenic plants and transgenic receptor lines were planted together and the replications were randomly distributed in the fields.

Blinding

The investigators were blinded to group allocation during data collection and analysis.

# Reporting for specific materials, systems and methods

We require information from authors about some types of materials, experimental systems and methods used in many studies. Here, indicate whether each material, system or method listed is relevant to your study. If you are not sure if a list item applies to your research, read the appropriate section before selecting a response.

## Materials & experimental systems

| n/a | Involved in the study |
|---|---|
| ☐ | ☒ Antibodies |
| ☒ | ☐ Eukaryotic cell lines |
| ☒ | ☐ Palaeontology and archaeology |
| ☒ | ☐ Animals and other organisms |
| ☒ | ☐ Clinical data |
| ☒ | ☐ Dual use research of concern |

## Methods

| n/a | Involved in the study |
|---|---|
| ☒ | ☐ ChIP-seq |
| ☒ | ☐ Flow cytometry |
| ☒ | ☐ MRI-based neuroimaging |

## Antibodies

Antibodies used

Antibody, supplier name, catalog number, clone name, lot number.
1. Mouse anti GFP-Tag mAb, ABclonal, #AE012, AMC0507, 9200012003;
2. Anti-Plant-actin Mouse Monoclonal Antibody, EASYBIO, #BE0028, Q30, 80870207;
3. Anti-Firefly Luciferase antibody (Goat), abcam, #ab181640, NA, GR3267949-18;
4. Mouse anti Myc-Tag mAb, ABclonal, #AE010, AMC0048, 3500014031;
5. Mouse anti MBP-Tag mAb, ABclonal, #AE016, AMC0505, 9200016002;
6. HRP-conjugated GST-tag Mouse mAb, YEASEN, #30903ES10, 3C10, H7227380;
7. HRP-conjugated His-tag Mouse mAb, YEASEN, #30404ES60, 9C11, H6227090;
8. Anti-Phospho-(Ser/Thr) antibody (Rabbit), abcam, #ab117253, NA, GR3376370-6;
9. Goat Anti-Rabbit IgG (H&L)-HRP Conjugated, EASYBIO, #BE0101, NA, 80780926;
10. Goat Anti-Mouse IgG (H&L)-HRP Conjugated, EASYBIO, #BE0102, NA, 80781014;
11. Rabbit Anti-Goat IgG (H&L)-HRP Conjugated, EASYBIO, #BE0103, NA, 80921201.

Validation

1. Mouse anti GFP-Tag mAb
WB: Nicotiana benthamiana (Manufacturer's websit: https://abclonal.com.cn/catalog/AE012), Zea mays (this study);
Co-IP: Nicotiana benthamiana (this study);
Reference: Yang Z, Huang Y, Yang J, Yao S, Zhao K, Wang D, Qin Q, Bian Z, Li Y, Lan Y, Zhou T, Wang H, Liu C, Wang W, Qi Y, Xu Z, Li Y. Jasmonate Signaling Enhances RNA Silencing and Antiviral Defense in Rice. Cell Host Microbe. 2020 Jul 8;28(1):89-103.e8. doi: 10.1016/j.chom.2020.05.001. PMID: 32504578;
Manufacturer's specification: https://abclonal.com.cn/Datasheet/Antibodies/AE012.pdf?v=1669714209.

2. Anti-Plant-actin Mouse Monoclonal Antibody
WB: Various Plant (Manufacturer's websit: http://www.bioeasytech.com/product/2363.html?goods_id=4251);
Specificity: Antibody can detects endogenous plant actin protein.

3. Anti-Firefly Luciferase antibody (Goat)
Suitable for WB: Nicotiana benthamiana (Manufacturer), Zea mays (this study)
Reference: Liu C, Cui D, Zhao J, Liu N, Wang B, Liu J, Xu E, Hu Z, Ren D, Tang D, Hu Y. Two Arabidopsis Receptor-like Cytoplasmic Kinases SZE1 and SZE2 Associate with the ZAR1-ZED1 Complex and Are Required for Effector-Triggered Immunity. Mol Plant. 2019 Jul 1;12(7):967-983. doi: 10.1016/j.molp.2019.03.012. PMID: 30947022;
The Key features and eatails are on Manufacturer's websit: https://www.abcam.cn/firefly-luciferase-antibody-ab181640.html?productWallTab=Abreviews.

4. Mouse anti Myc-Tag mAb
WB: Zea mays, Nicotiana benthamiana (Manufacturer's websit: https://abclonal.com.cn/catalog/AE010);
Co-IP: Nicotiana benthamiana (this study);
Reference: Yang Z, Huang Y, Yang J, Yao S, Zhao K, Wang D, Qin Q, Bian Z, Li Y, Lan Y, Zhou T, Wang H, Liu C, Wang W, Qi Y, Xu Z, Li Y. Jasmonate Signaling Enhances RNA Silencing and Antiviral Defense in Rice. Cell Host Microbe. 2020 Jul 8;28(1):89-103.e8. doi:

10.1016/j.chom.2020.05.001. PMID: 32504578;
Manufacturer's specification: https://abclonal.com.cn/Datasheet/Antibodies/AE010.pdf?v=1675416399.

5. Mouse anti MBP-Tag mAb
Manufacturer's websit: https://abclonal.com.cn/catalog/AE016;
Refeence: Xiong J, Yang F, Yao X, Zhao Y, Wen Y, Lin H, Guo H, Yin Y, Zhang D. The deubiquitinating enzymes UBP12 and UBP13 positively regulate recovery after carbon starvation by modulating BES1 stability in Arabidopsis thaliana. Plant Cell. 2022 Oct 27;34(11):4516-4530. doi: 10.1093/plcell/koac245. PMID: 35944221;
Manufacturer's specification: https://abclonal.com.cn/Datasheet/Antibodies/AE016.pdf?v=1666064798.

6. HRP-conjugated GST-tag Mouse mAb, YEASEN, #30903ES10, NA, NA;
Application: WB (https://www.yeasen.com/products/detail/170);
Manufacturer's specification: https://upload.yeasen.com/website/file/20180830123807687.pdf.

7. HRP-conjugated His-tag Mouse mAb
Application: WB (https://www.yeasen.com/products/detail/86);
Reference: Tan FQ, Wang W, Li J, Lu Y, Zhu B, Hu F, Li Q, Zhao Y, Zhou DX. A coiled-coil protein associates Polycomb Repressive Complex 2 with KNOX/BELL transcription factors to maintain silencing of cell differentiation-promoting genes in the shoot apex. Plant Cell. 2022 Jul 30;34(8):2969-2988. doi: 10.1093/plcell/koac133. PMID: 35512211;
Manufacturer's specification: https://upload.yeasen.com/website/file/20180827222215846.pdf.

8. Anti-Phospho-(Ser/Thr) antibody
WB application in Reference: Peng X, Wang M, Li Y, Yan W, Chang Z, Chen Z, Xu C, Yang C, Deng XW, Wu J, Tang X. Lectin receptor kinase OsLecRK-S.7 is required for pollen development and male fertility. J Integr Plant Biol. 2020 Aug;62(8):1227-1245. doi: 10.1111/jipb.12897. PMID: 31833176 [Protein phosphorylation was detected by immunoblotting with anti-phospho Ser/Thr antibodies (Abcam, Cat#ab117253)]; Nicotiana benthamiana, Zea mays (this study);
The Key features and eatails are on Manufacturer's websit: https://www.abcam.cn/phospho-serthr-antibody-ab117253.html.

9. Goat Anti-Rabbit IgG (H&L)-HRP Conjugated
Application: WB (http://www.bioeasytech.com/product/2901.html?goods_id=5786).

10. Goat Anti-Mouse IgG (H&L)-HRP Conjugated
Application: WB (http://www.bioeasytech.com/product/2907.html?goods_id=5794);
Reference: Liu Q, Deng S, Liu B, Tao Y, Ai H, Liu J, Zhang Y, Zhao Y, Xu M. A helitron-induced RabGDIα variant causes quantitative recessive resistance to maize rough dwarf disease. Nat Commun. 2020 Jan 24;11(1):495. doi: 10.1038/s41467-020-14372-3. PMID: 31980630.

11. Rabbit Anti-Goat IgG (H&L)-HRP Conjugated
Application: WB (http://www.bioeasytech.com/product/2914.html?goods_id=5803).
