# Supplementary material for: The ZmWAKL–ZmWIK–ZmBLK1–ZmRBOH4 module provides quantitative resistance to gray leaf spot in maize
Source: Nat Genet. 2024 Jan 18;56(2):315–26. doi: 10.1038/s41588-023-01644-z (PMC10864183; doi:10.1038/s41588-023-01644-z)

Figs. 1-6 Uncropped images.

Figure 1c

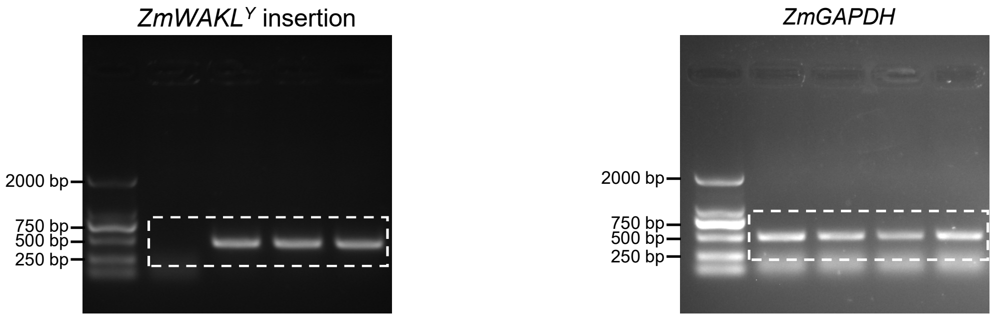

Figure 1f

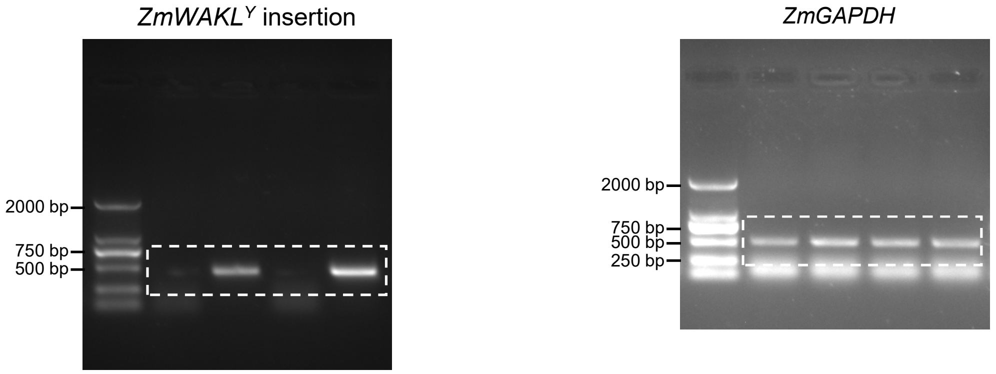

Figure 1j

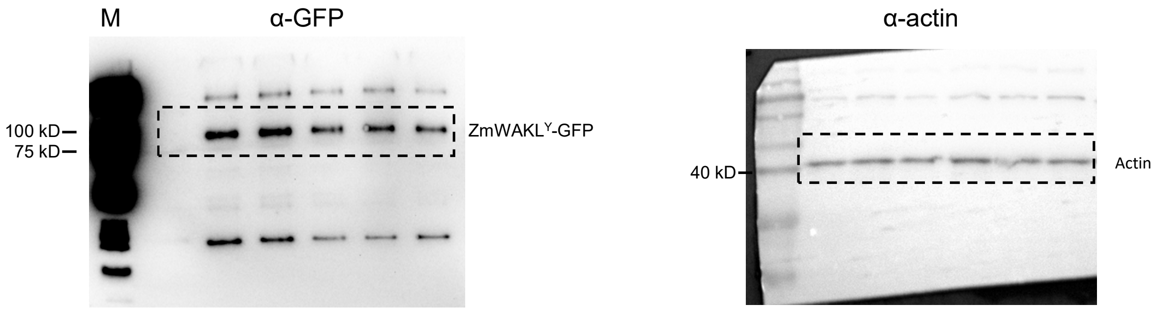

Figure 1m

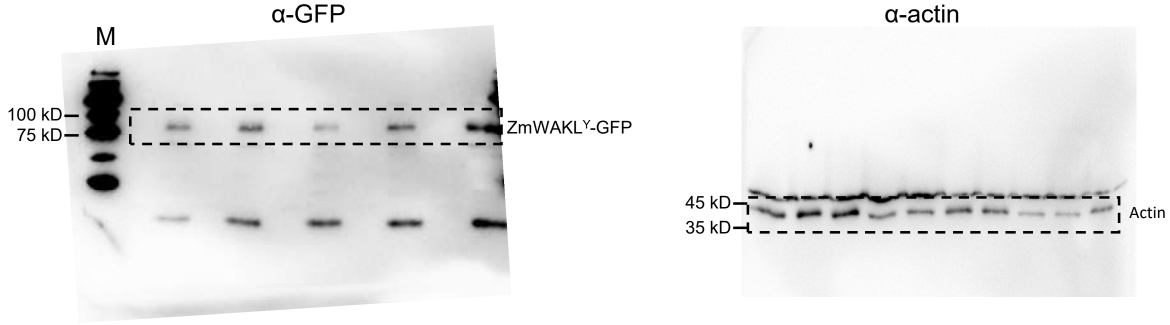

Figure 2d

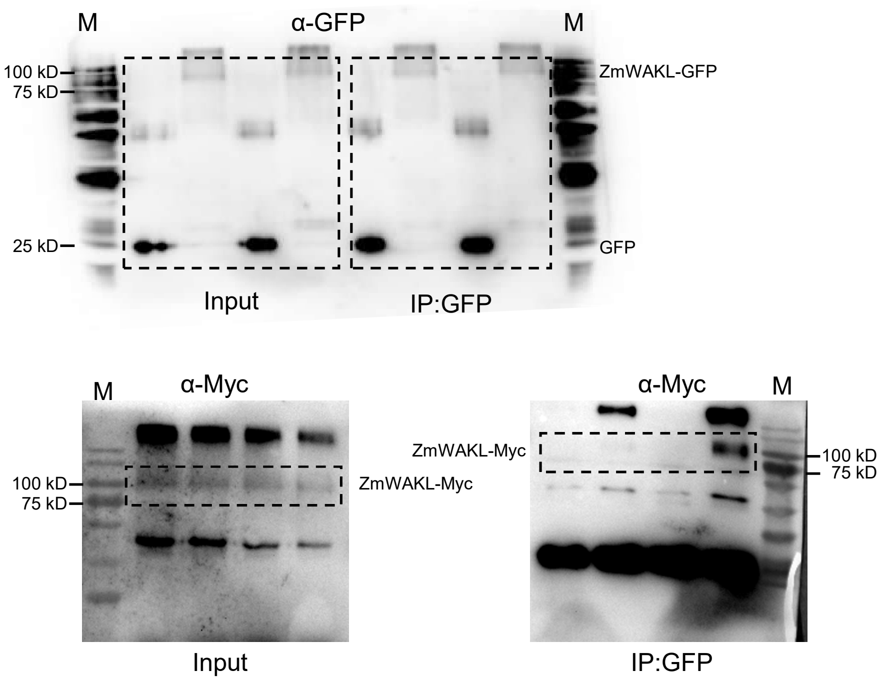

Figure 2g

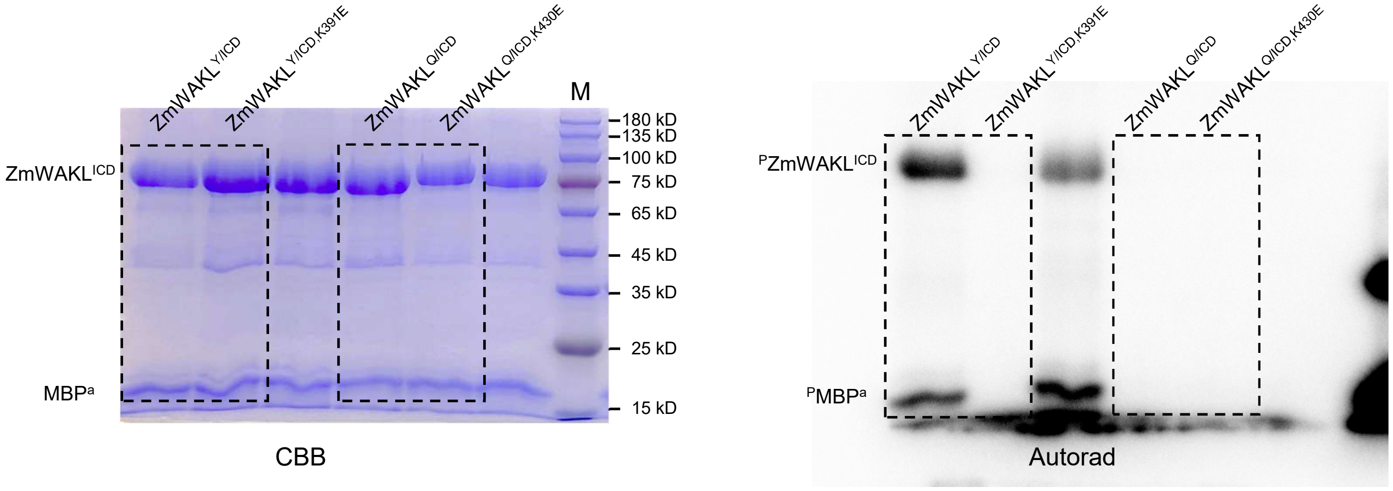

Figure 2h

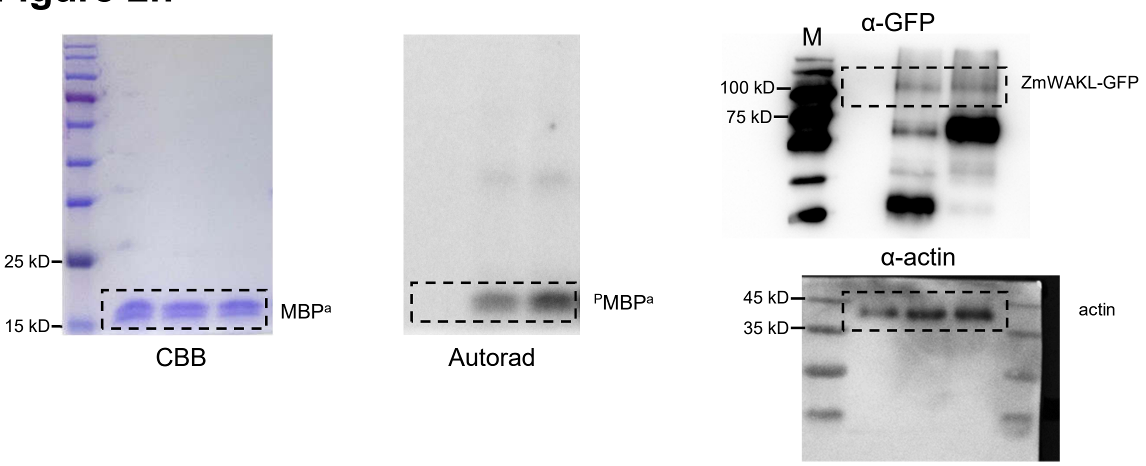

Figure 3c

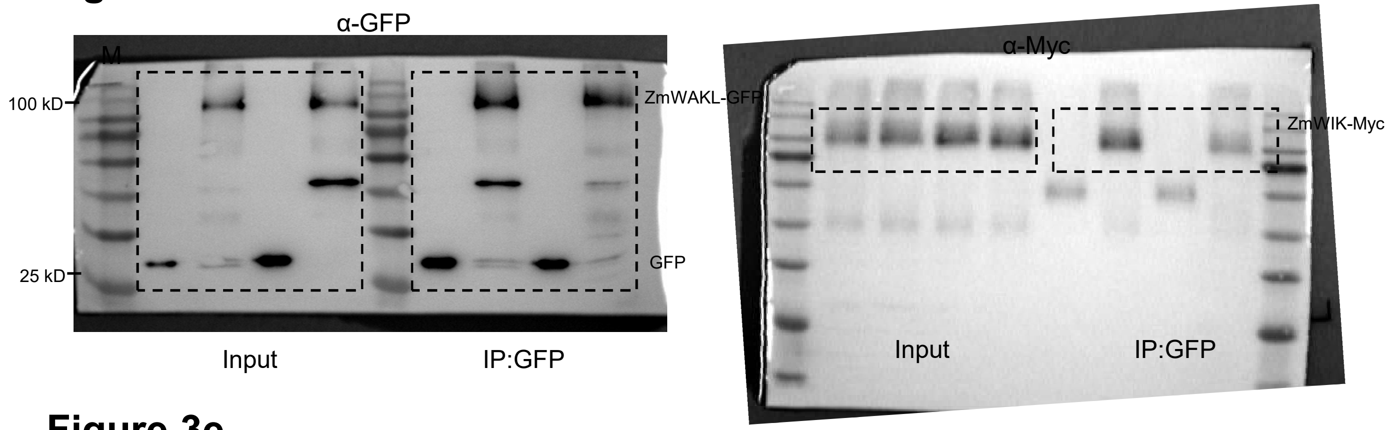

Figure 3e

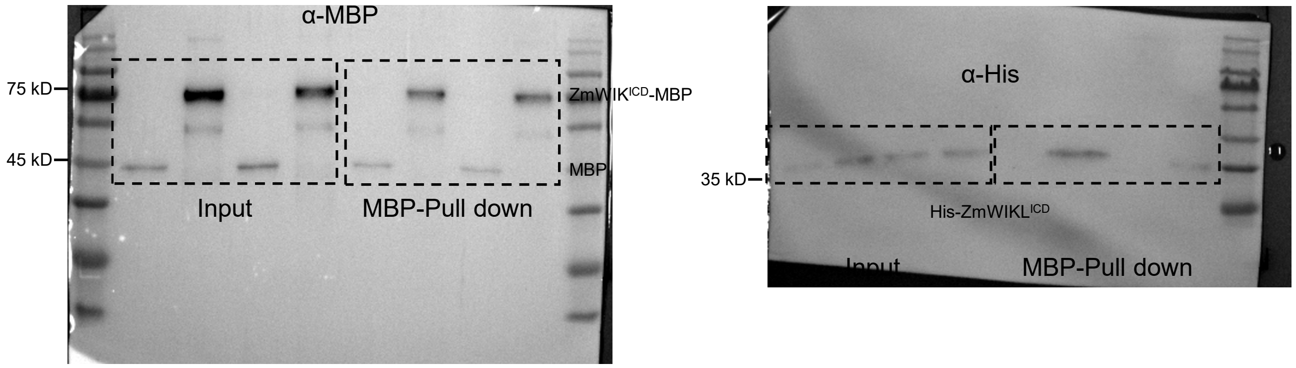

Figure 3f

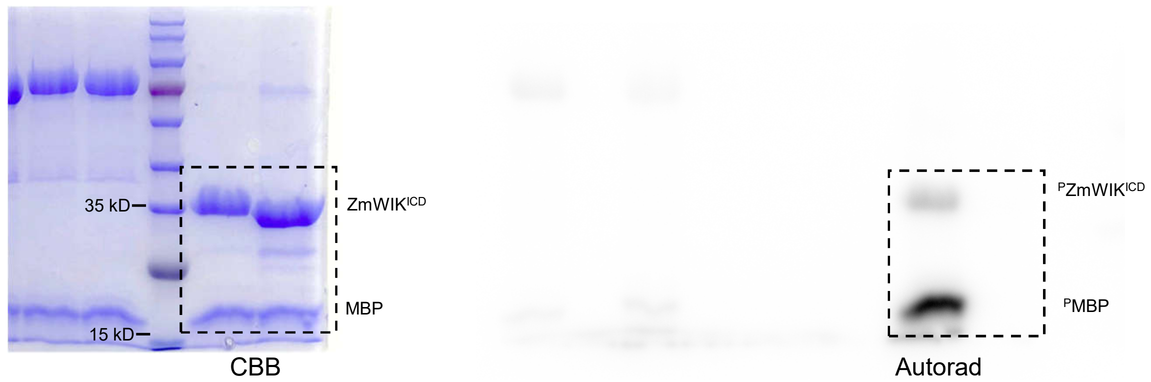

Figure 3g

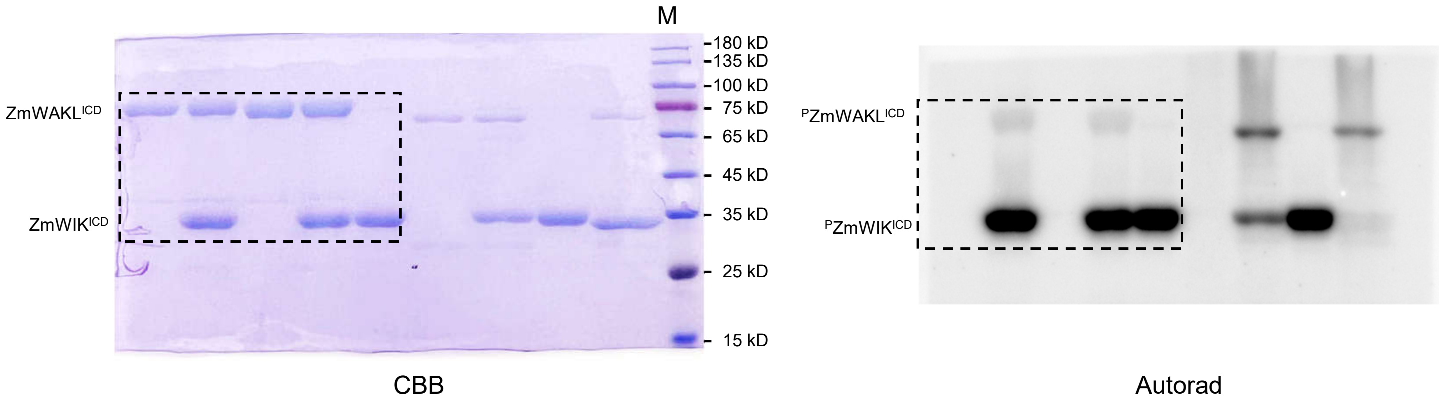

Figure 3h

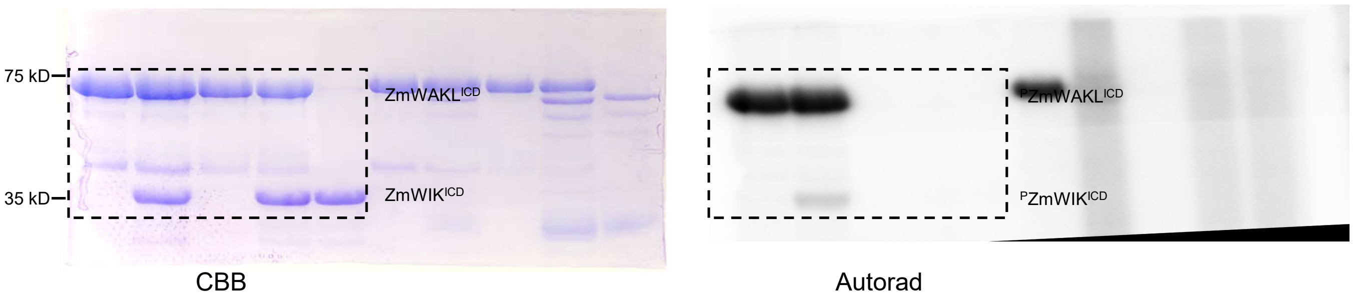

Figure 3i

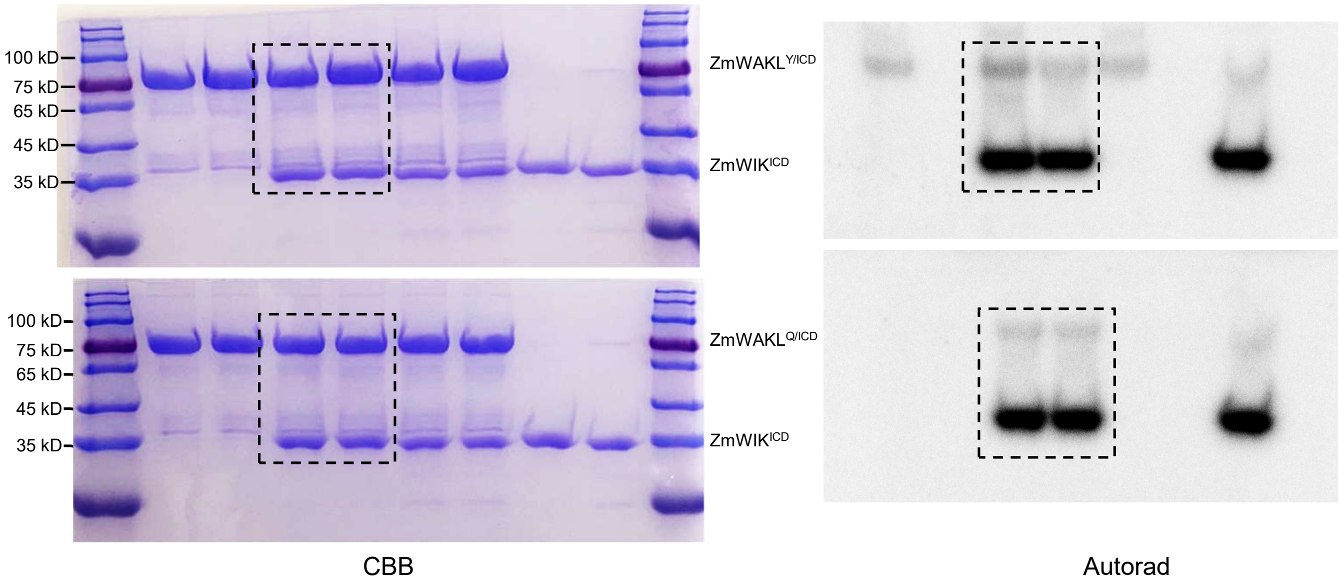

Figure 3j

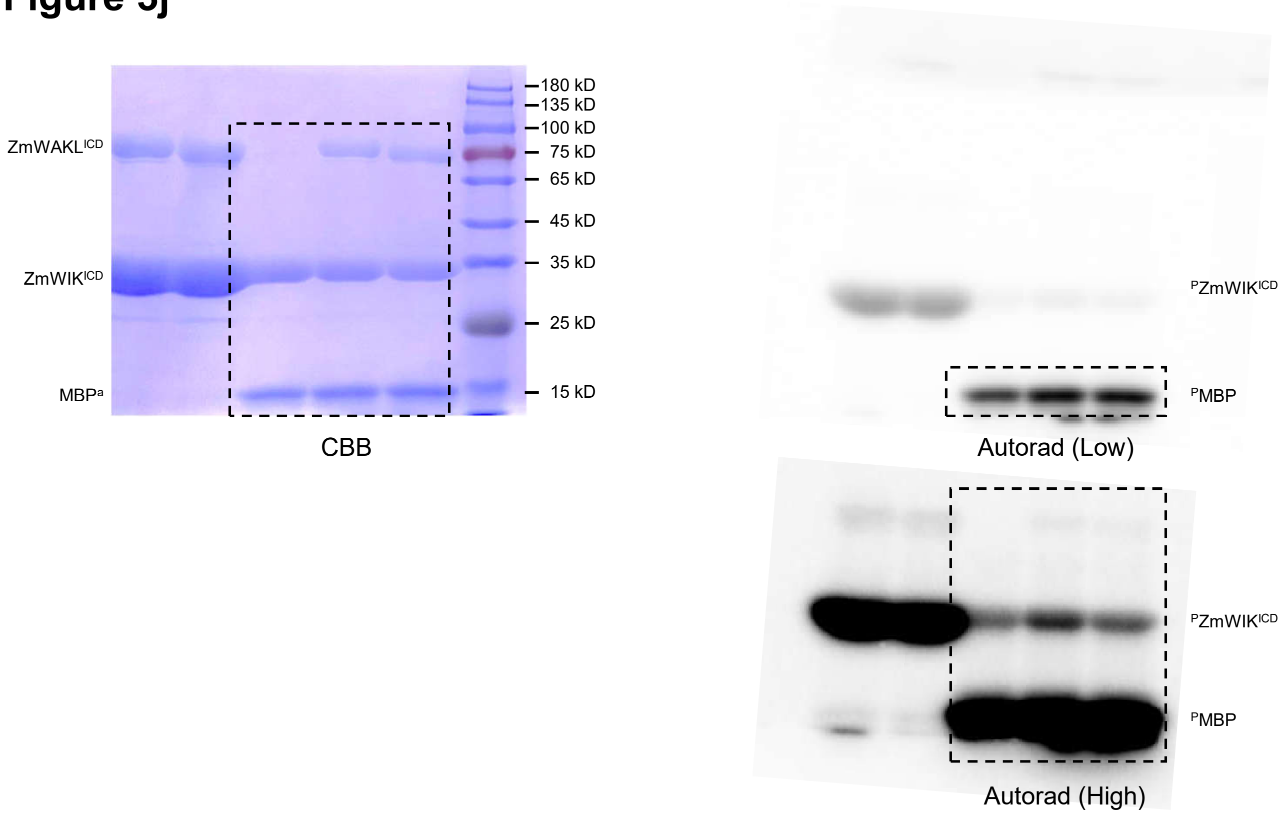

Figure 4b

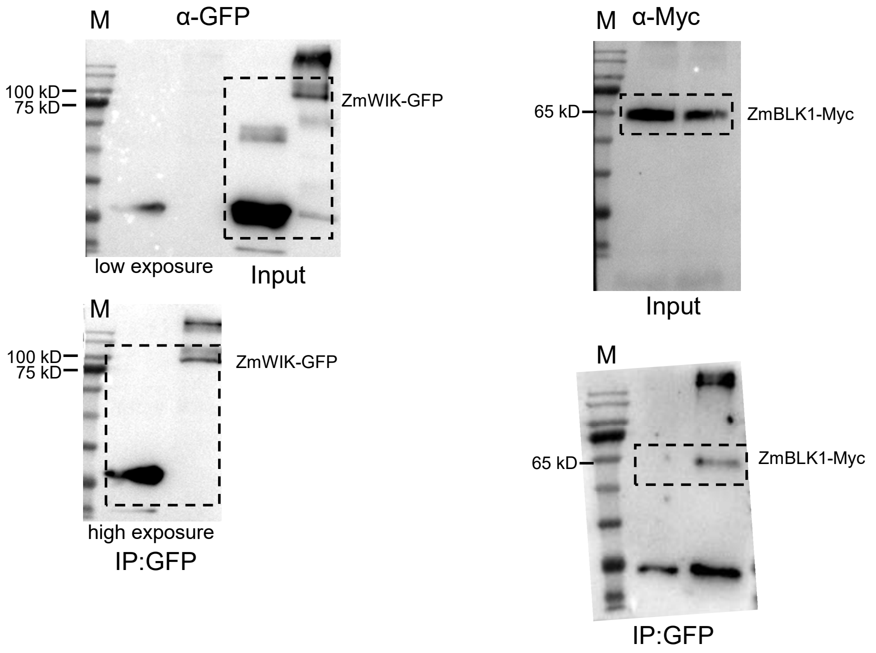

Figure 4c

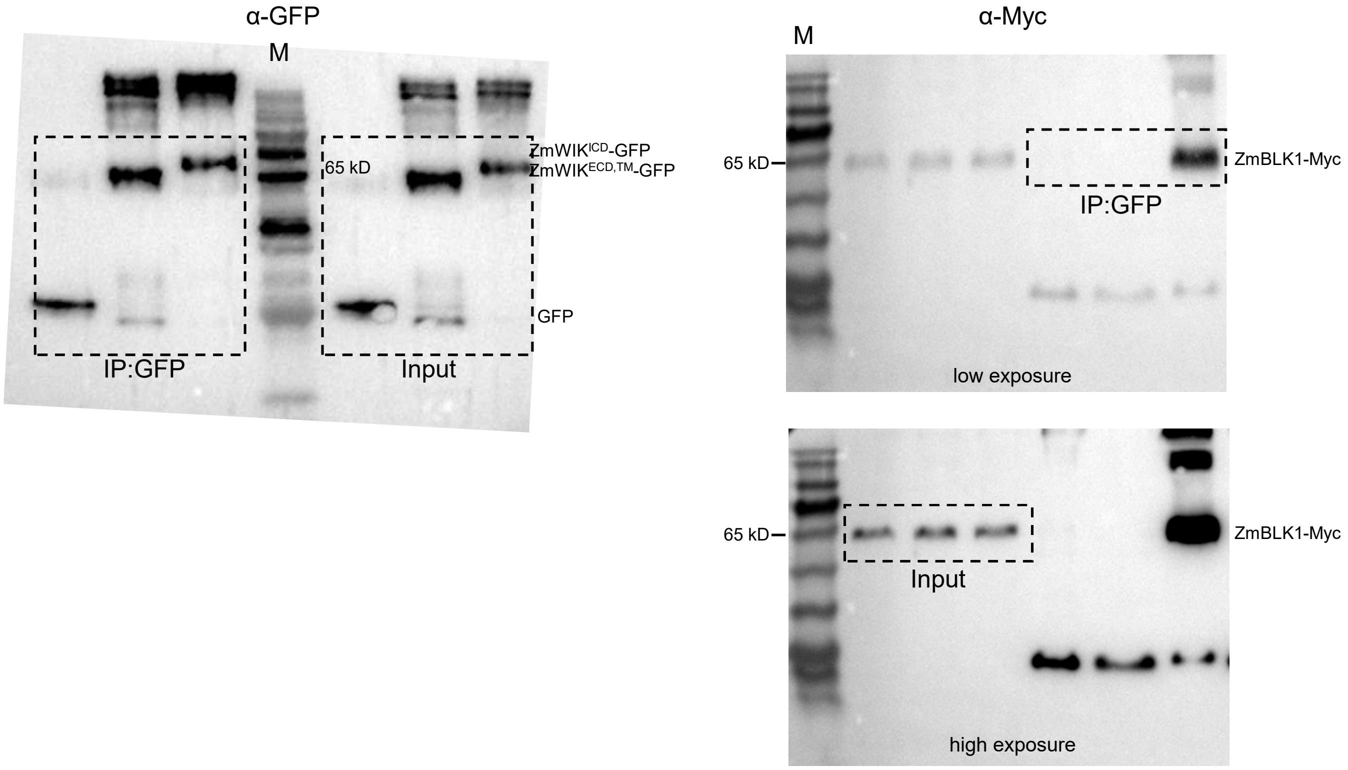

Figure 4d

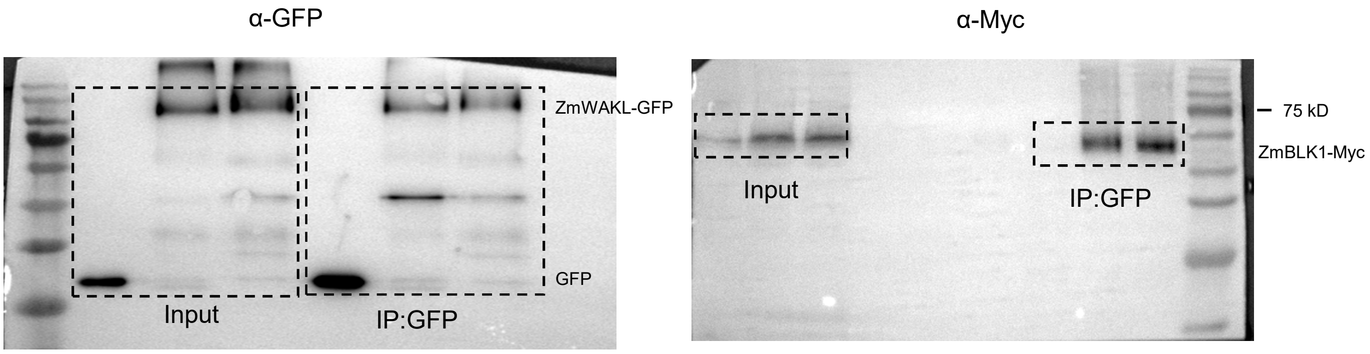

Figure 4e

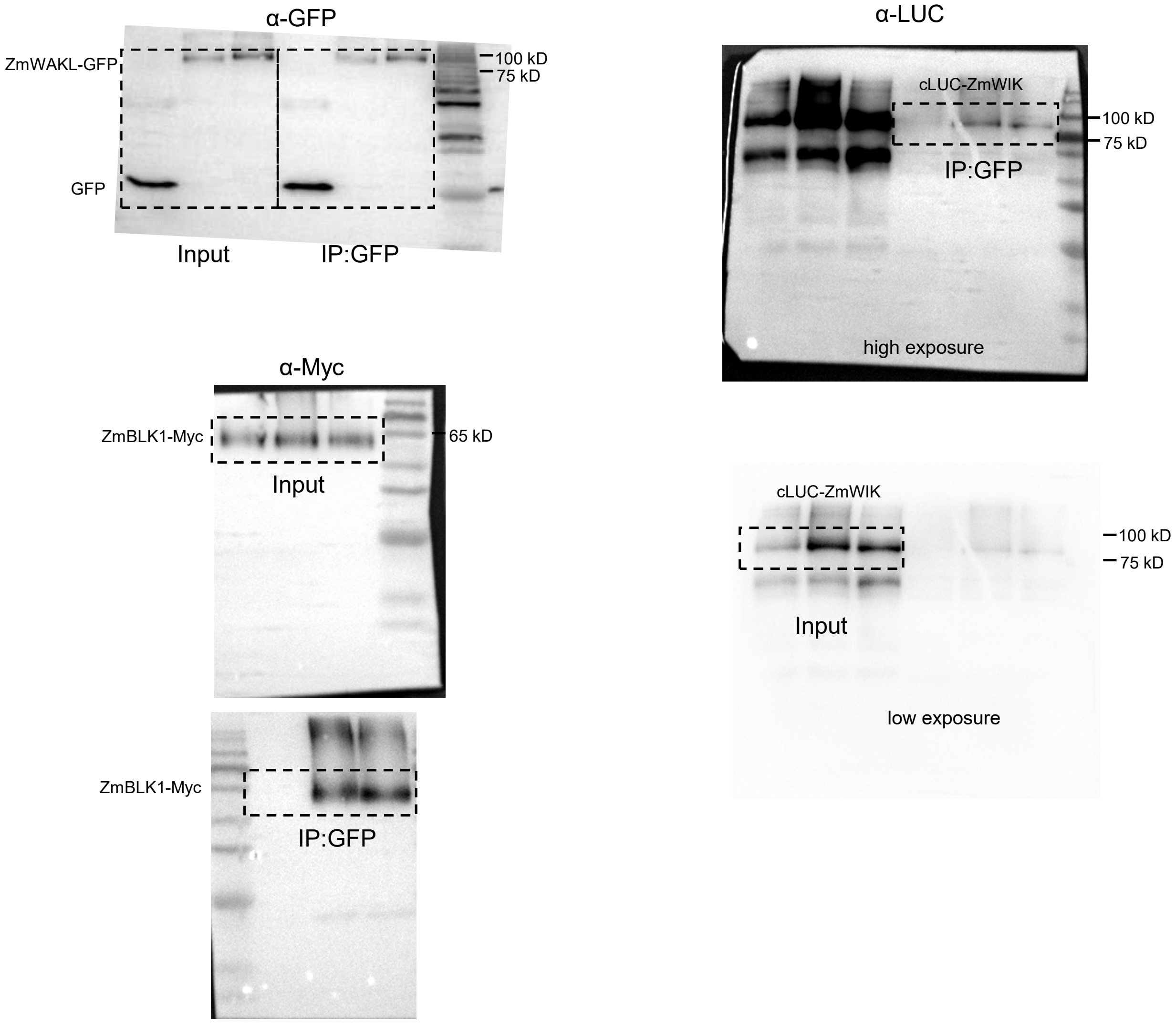

Figure 4f

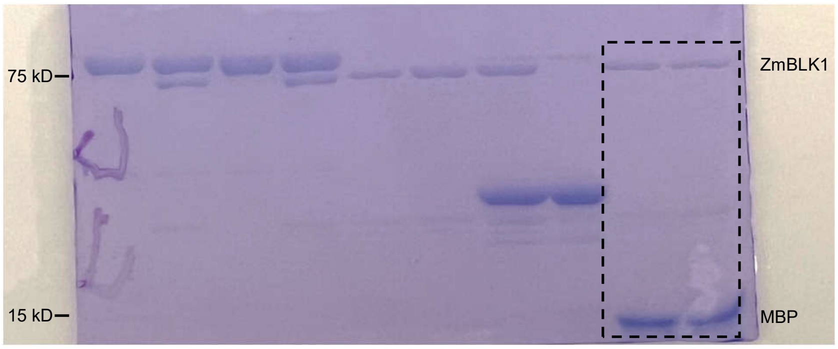

CBB

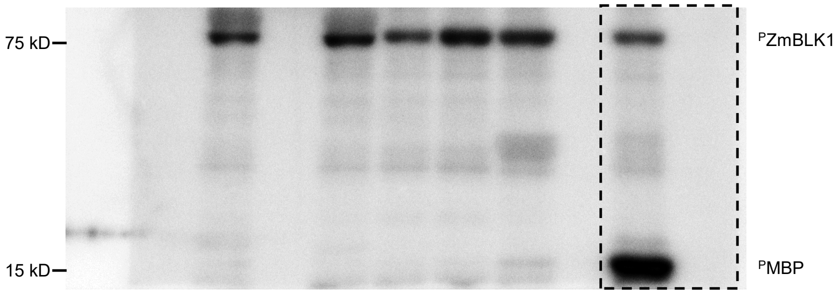

Autorad

Figure 4g

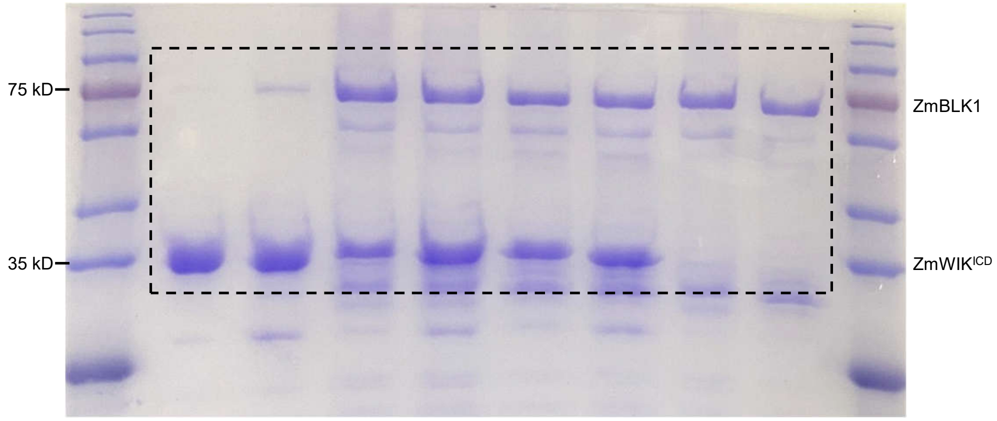

CBB

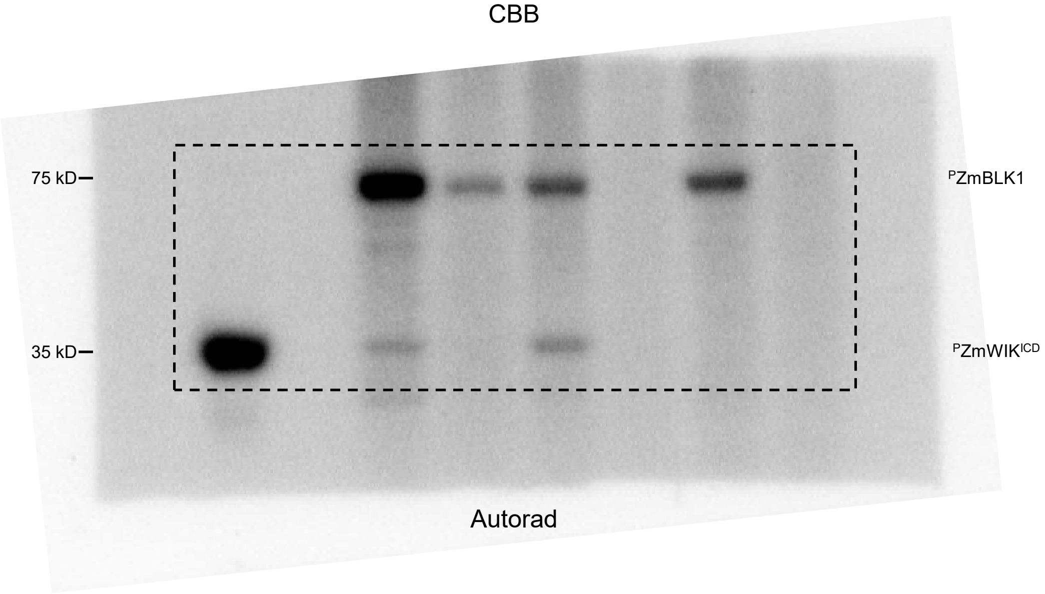

Autorad

Figure 4h

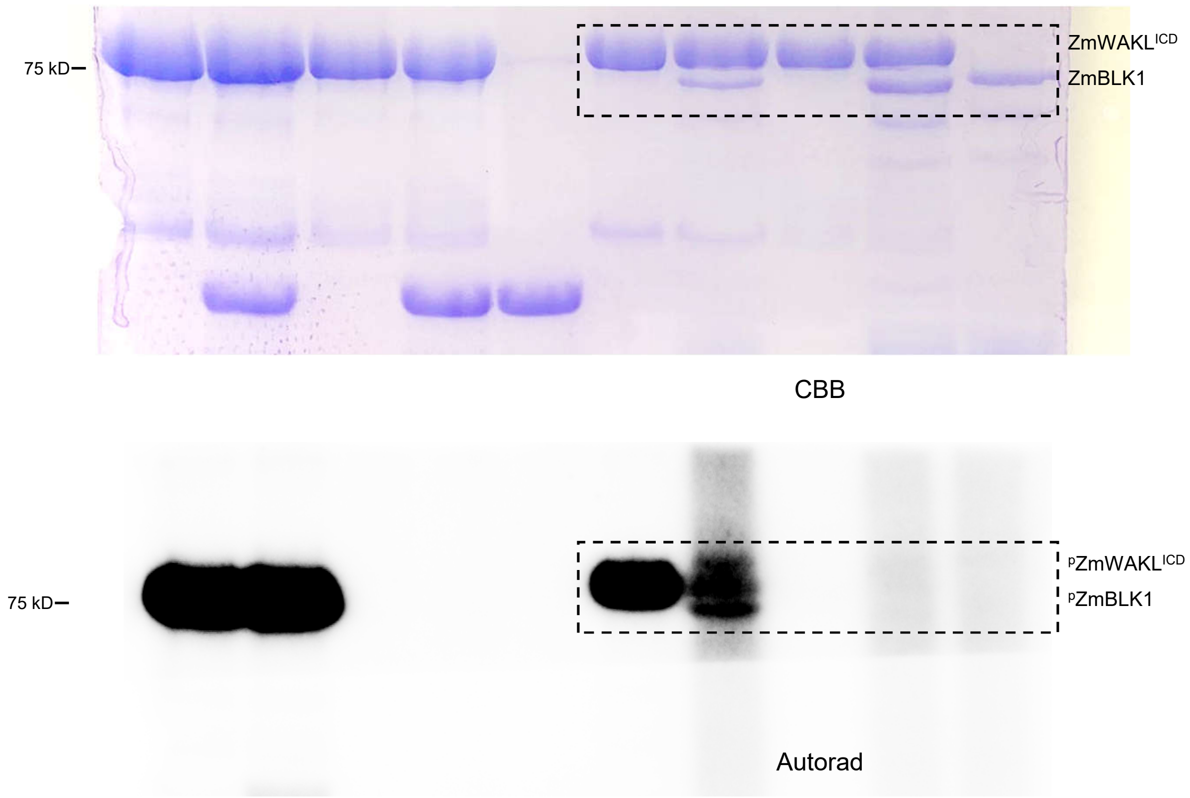

Figure 4i

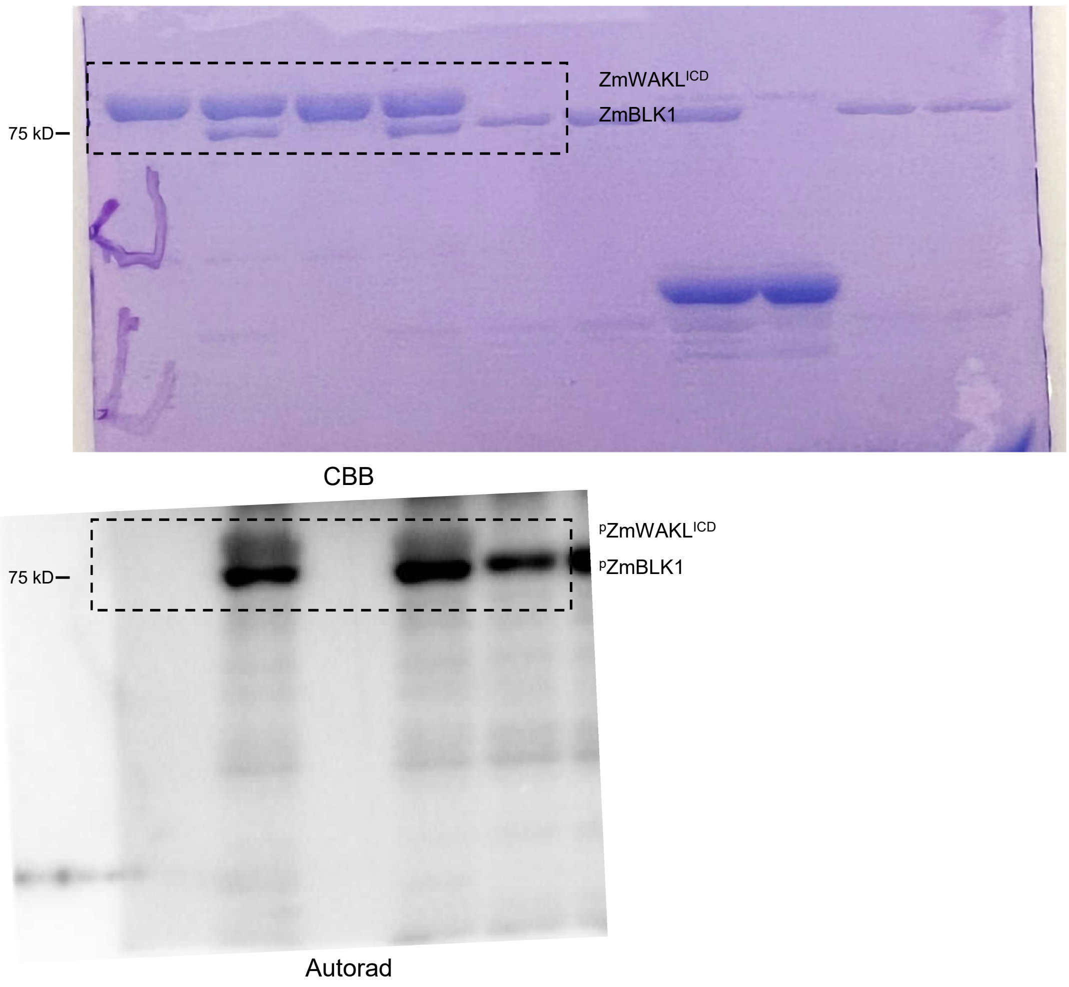

Figure 4j

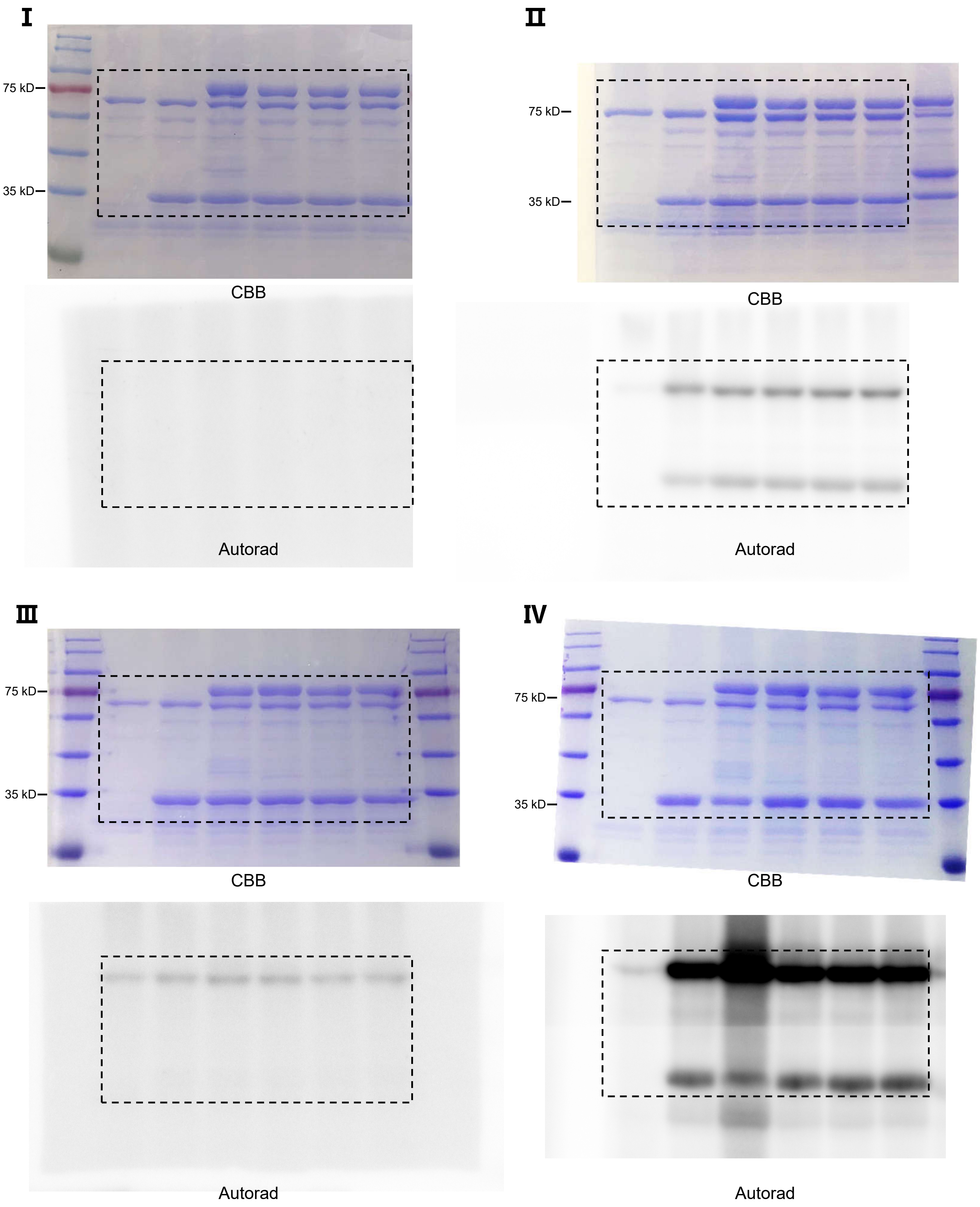

I
75 kD
35 kD
CBB
Autorad
II
75 kD
35 kD
CBB
Autorad
III
75 kD
35 kD
CBB
Autorad
IV
75 kD
35 kD
CBB
Autorad

Figure 5b

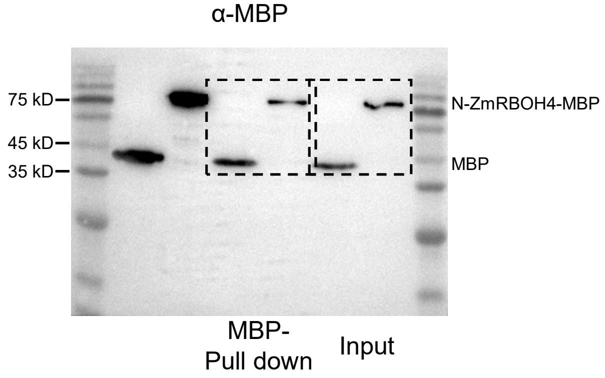

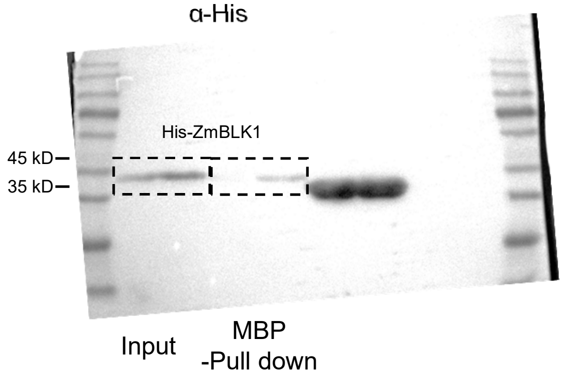

Figure 5c

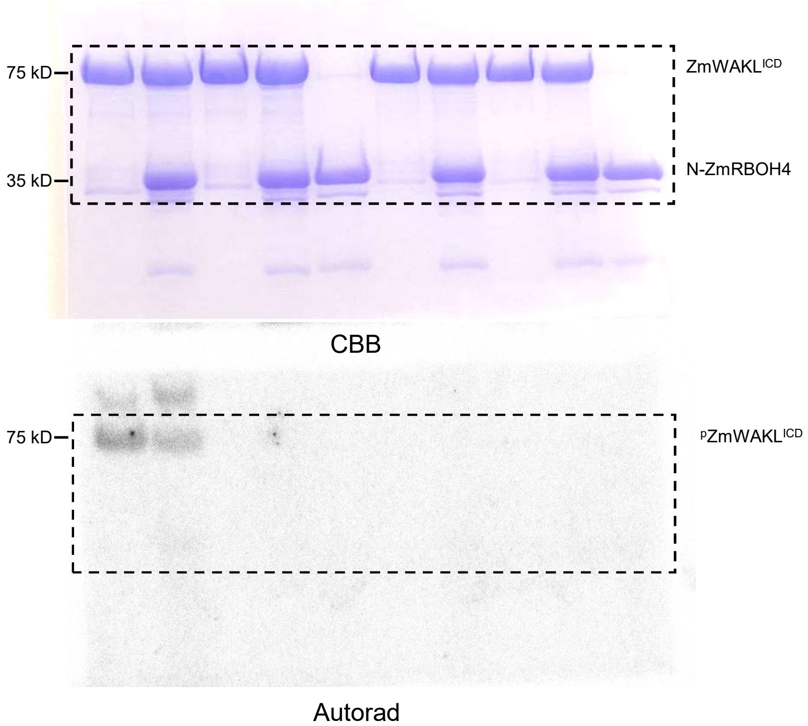

Figure 5d Figure 5e

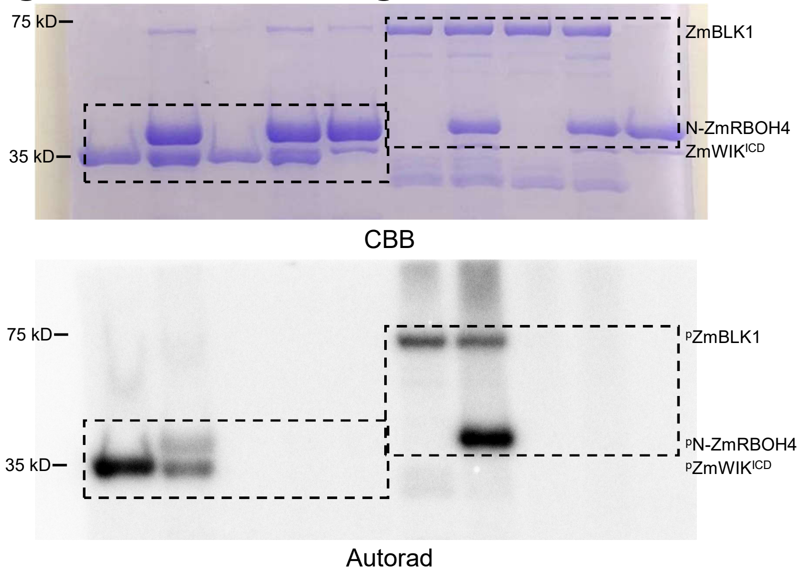

Figure 5f

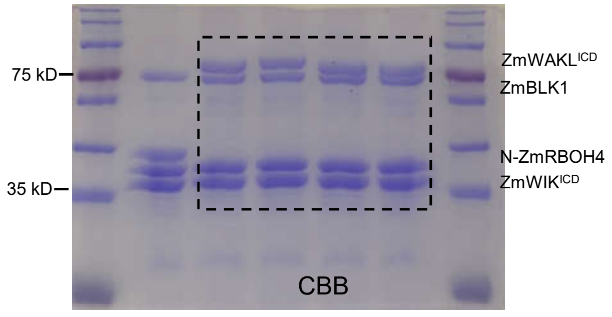

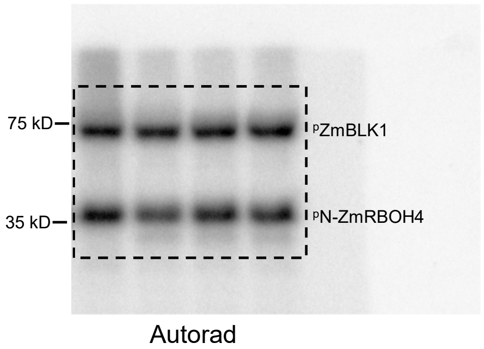

Figure 5g

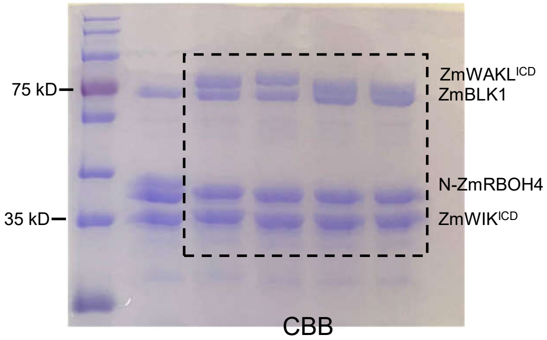

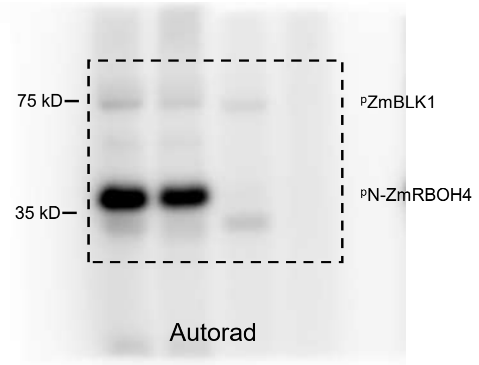

Figure 6a

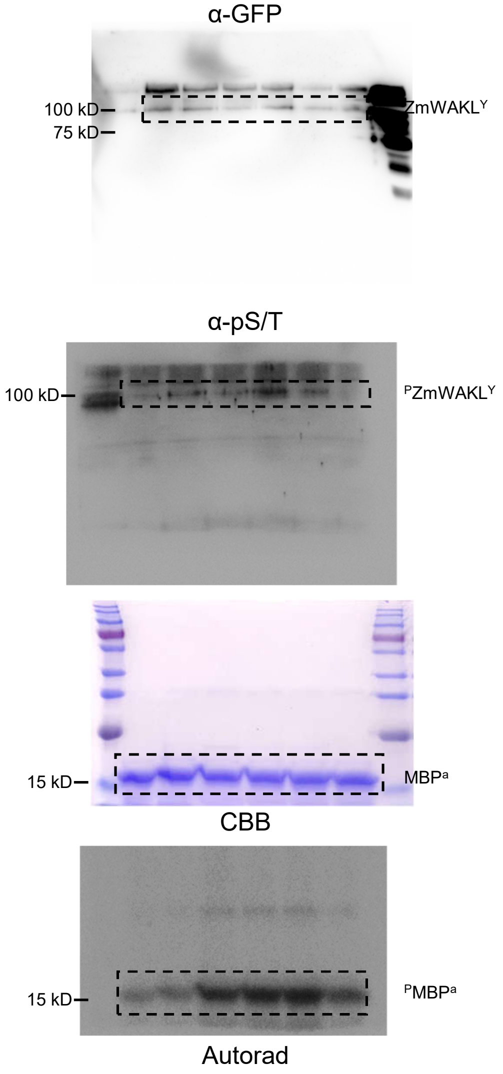

Figure 6b

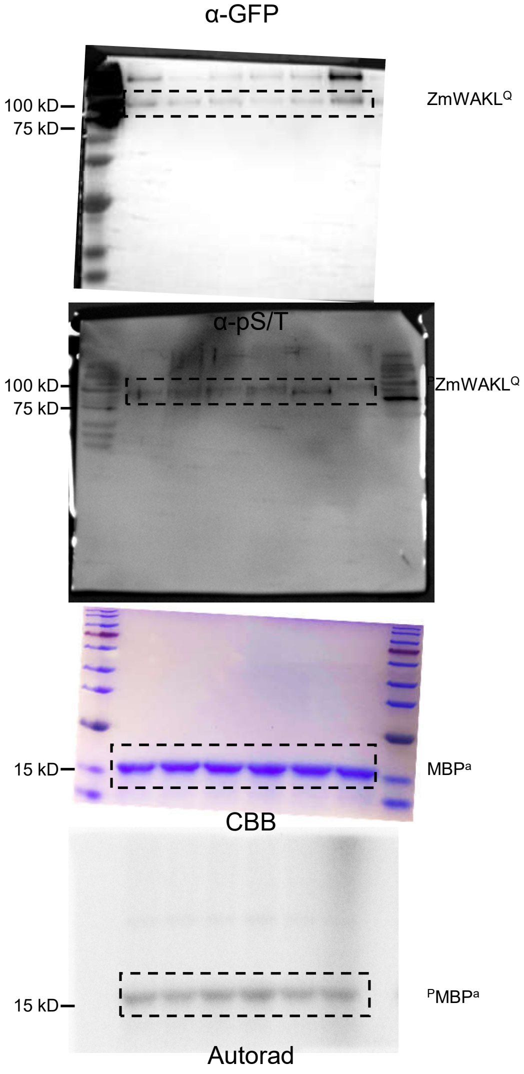

Uncropped images of extended data Figs. 4, 8, 9.

Extended Data Fig. 4b

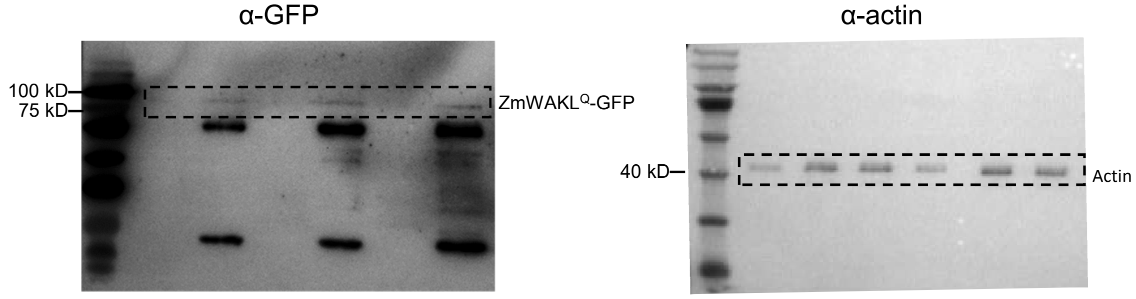

Extended Data Fig. 8b

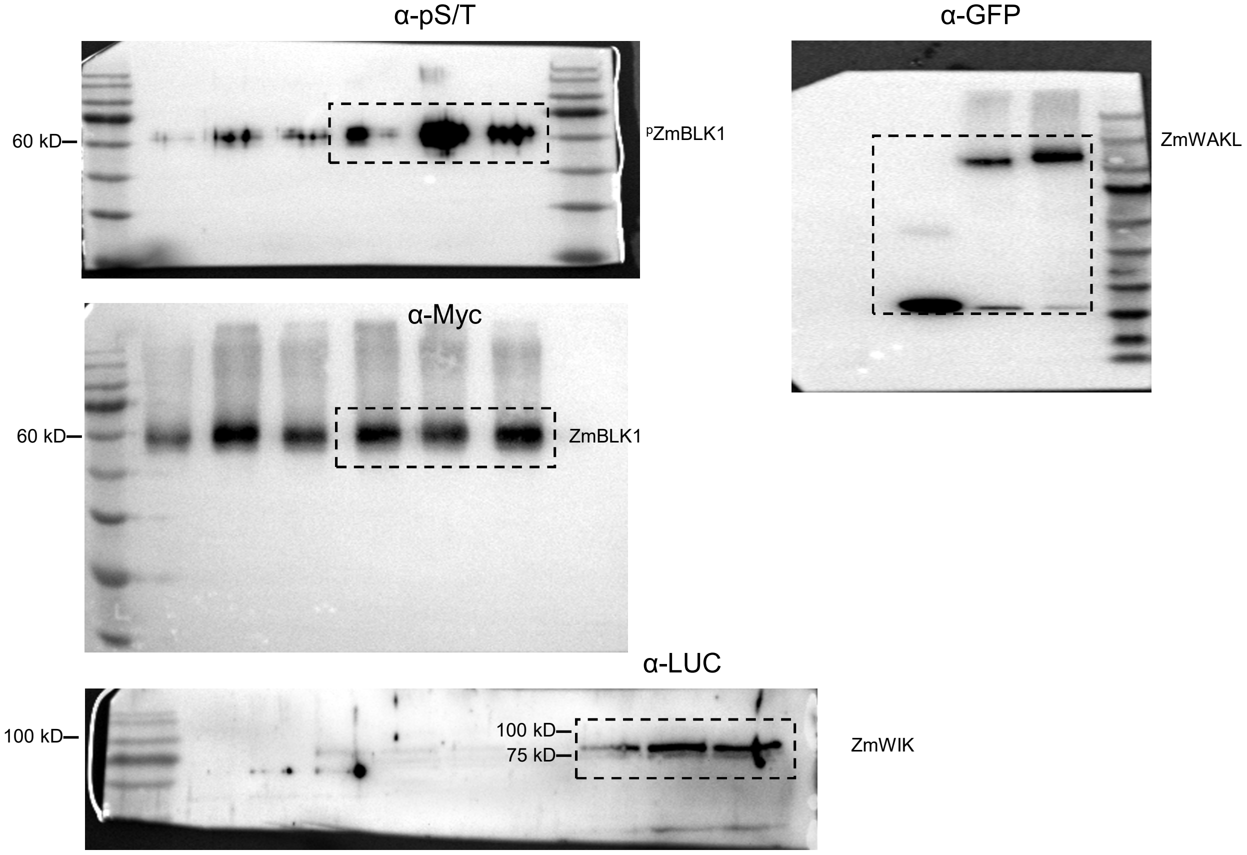

Extended Data Fig. 8c

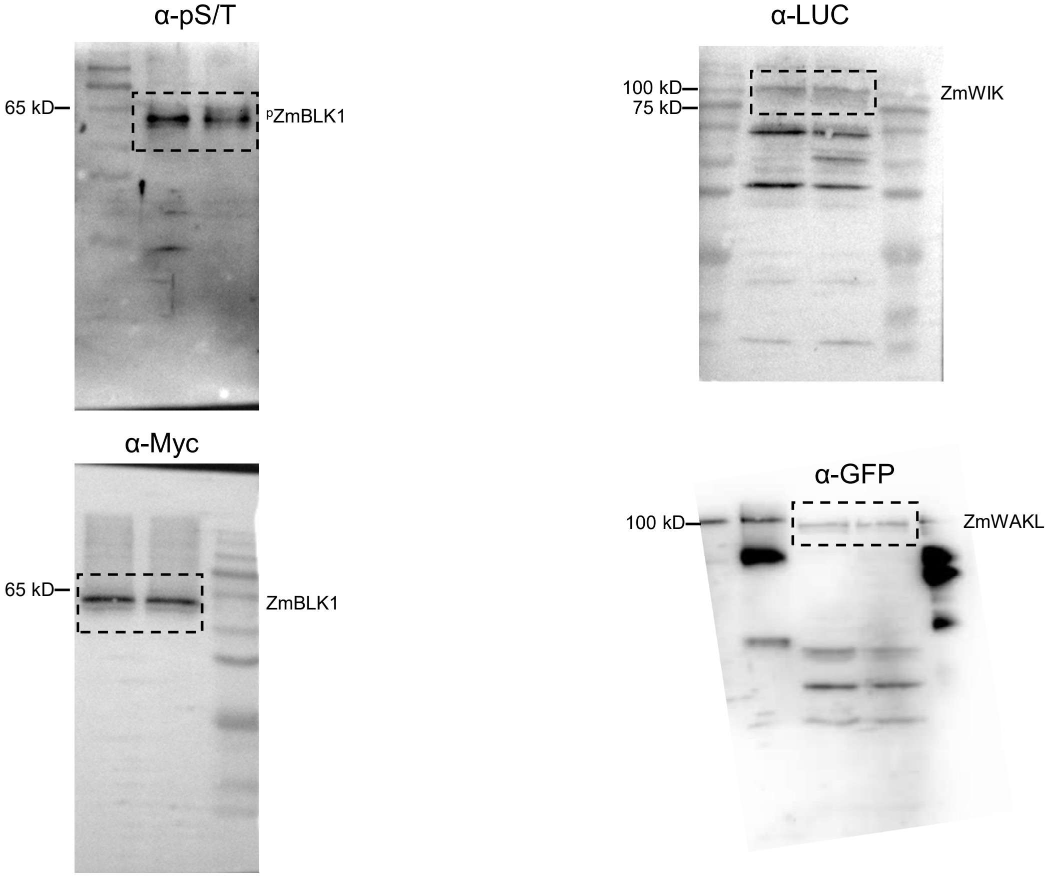

Extended Data Fig. 8d

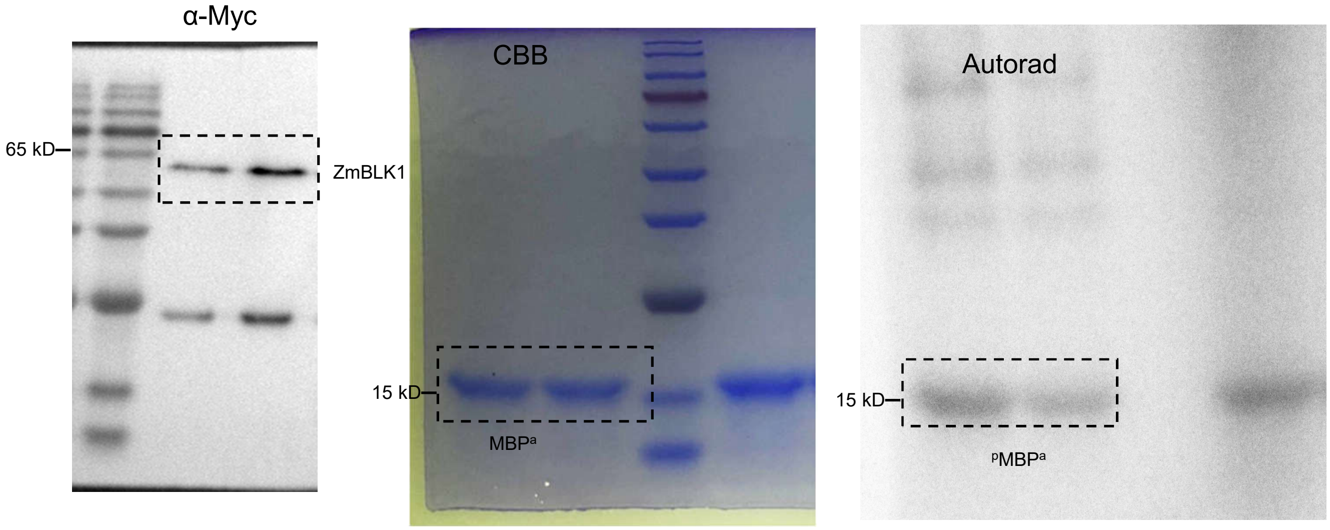

Extended Data Fig. 9d

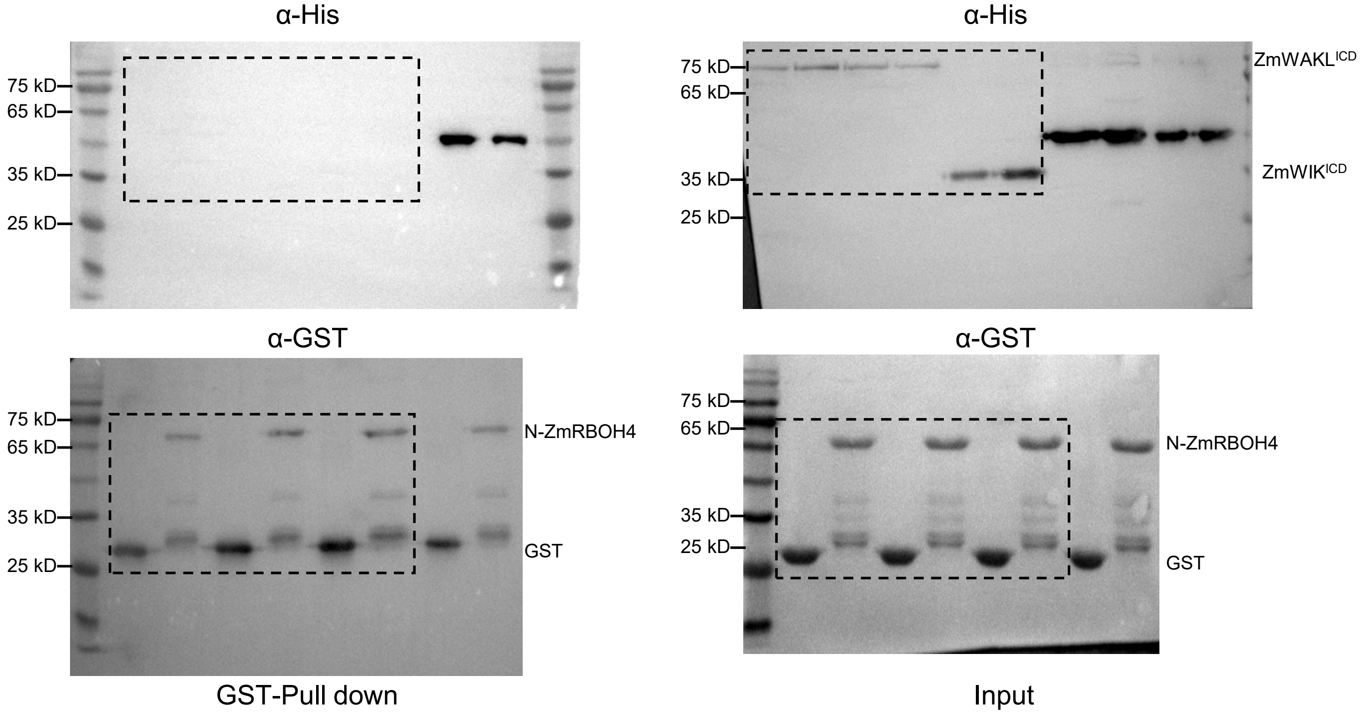

Supplement: Supplementary file 18 — Unprocessed western blots and gels. [file 41588_2023_1644_MOESM18_ESM.pdf]
